# ﻿Flora of Mongolia: annotated checklist of native vascular plants

**DOI:** 10.3897/phytokeys.192.79702

**Published:** 2022-03-14

**Authors:** Shukherdorj Baasanmunkh, Magsar Urgamal, Batlai Oyuntsetseg, Alexander P. Sukhorukov, Zagarjav Tsegmed, Dong Chan Son, Andrey Erst, Khurelpurev Oyundelger, Alexey A. Kechaykin, Joscelyn Norris, Petr Kosachev, Jin-Shuang Ma, Kae Sun Chang, Hyeok Jae Choi

**Affiliations:** 1 Department of Biology and Chemistry, Changwon National University, Changwon 51140, Republic of Korea Changwon National University Changwon Republic of Korea; 2 Laboratory of Plant Systematics and Phylogeny, Botanic Garden and Research Institute, Mongolian Academy of Sciences, Ulaanbaatar 13330, Mongolia Botanic Garden and Research Institute, Mongolian Academy of Sciences Ulaanbaatar Mongolia; 3 Department of Biology, School of Arts and Science, National University of Mongolia, Ulaanbaatar 14201, Mongolia National University of Mongolia Ulaanbaatar Mongolia; 4 Moscow State University, Leninskiye Gory, 1/12, Moscow, 119234, Russia Moscow State University Moscow Russia; 5 Tomsk State University, Lenina Pr., 36, Tomsk, 634050, Russia Tomsk State University Tomsk Russia; 6 Division of Forest Biodiversity, Korea National Arboretum, Pocheon 11186, Republic of Korea Division of Forest Biodiversity, Korea National Arboretum Pocheon Republic of Korea; 7 Central Siberian Botanical Garden SB RAS, Zolotodolinskaya St. 101, Novosibirsk, 630090, Russia Central Siberian Botanical Garden SB RAS Novosibirsk Russia; 8 Technical University Dresden, International Institute (IHI) Zittau, Chair of Biodiversity of Higher Plants, 02763, Zittau, Germany Technical University Dresden Zittau Germany; 9 Department of Botany, Senckenberg Museum of Natural History Görlitz, 02826, Görlitz, Germany Department of Botany, Senckenberg Museum of Natural History Görlitz Görlitz Germany; 10 South Siberian Botanical Garden, Altai State University, Lenina 61, Barnaul, 656049, Russia Altai State University Barnaul Russia; 11 Rubenstein School of Environment and Natural Resources, University of Vermont, Vermont 05405-0088, USA University of Vermont Vermont United States of America; 12 Institute of Botany, Beijing Botanical Garden, Beijing 100093, China Institute of Botany, Beijing Botanical Garden Beijing China; 13 DMZ Forest and Biological Resources Conservation Division, Korea National Arboretum, Yanggu 24564, Republic of Korea DMZ Forest and Biological Resources Conservation Division, Korea National Arboretum Yanggu Republic of Korea

**Keywords:** Checklist, flora of Mongolia, native taxa, vascular plants

## Abstract

In this study, we critically revised and updated the checklist of native vascular plants of Mongolia. The checklist comprises 3,041 native vascular plant taxa (2,835 species and 206 infraspecific species) from 653 genera and 111 families, including 7 lycophytes, 41 ferns, 21 gymnosperms, and 2,972 angiosperms. In the angiosperms, we identified the 14 families with the greatest species richness, ranging from 50 to 456 taxa. Species endemism is also noted here; 102 taxa are endemic to Mongolia, and 275 taxa are sub-endemic that co-occur in adjacent countries. Since 2014, a total of 14 taxa have been described new to science based on morphological evidences. Moreover, five genera and 74 taxa were newly added to the flora of Mongolia. Based on our critical revisions, names of three families, 21 genera, and 230 species have been changed in comparison to the previous checklist, “Conspectus of the vascular plants of Mongolia” (2014).

## ﻿Introduction

Mongolia is located in the mid-latitude (between 41°35'N–52°09'N and 87°44'E–119°56'E), between Russia and China, covering approximately 1.6 million km^2^, roughly equivalent to the size of western and central Europe. The flora of Mongolia is comprised of native species of different origins including boreal, steppe, desert, and mountainous elements of vegetation (Hilbig 1995; Gunin et al. 1999). The country is divided into sixteen phytogeographical regions which have various vegetation types (Grubov and Yunatov 1952), namely, alpine steppe, forest, meadow steppe, typical steppe, desert steppe, and desert (Gunin et al. 1999; Baasanmunkh et al. 2021a). Mongolia has a significant amount of temperate grasslands and semi-arid desert (Gunin et al. 1999; Wesche et al. 2016), which cover about 80% of the country’s area (Pfeiffer et al. 2020). Overall, the species richness of vascular plants in Mongolia is not particularly high compared to other countries in Asia (Du et al. 2020; Li et al. 2020; Wang et al. 2020). However, Mongolia has the world’s largest intact grassland with respect to its biodiversity (Batsaikhan et al. 2014; Hurka et al. 2019), which has great importance for the preservation of native vascular plants.

### ﻿A brief history of listing of the flora of Mongolia and recent taxonomic revisions

Historically, floristic studies have been very thoroughly conducted in this country, although recent updates are continuously being made. The first checklist of vascular plants included 1,897 species belonging to 555 genera and 97 families (Grubov 1955). Then, Grubov (1982) updated the checklist of vascular plants, which included 2,239 taxa from 599 genera and 103 families, with an identification key and information on their regional distribution and representative habitats. Later, Gubanov (1996) published a checklist with 2,823 higher plant species from 662 genera and 128 families, including notes on their regional distribution. More recently, Urgamal et al. (2014) updated the families of vascular flora according to APG III, with a total of 3,127 taxa that belong to 683 genera and 112 families. Since 2009, nine volumes with selected families have been published by Mongolian botanists, including Cyperaceae (Nyambayar 2009a), Apiaceae to Cornaceae (Urgamal 2009), Huperziaceae to Ephedraceae (Ulziikhutag et al. 2015), Asteraceae (Dariimaa 2014, 2021; Dariimaa and Saruul 2017), Ceratophyllaceae to Zygophyllaceae (Urgamal et al. 2020), Amaranthaceae s.l. (incl. Chenopodiacceae) (Tungalag 2020), Nymphaeaceae to Asphodelaceae (Urgamal et al. 2021).

Additionally, several families and genera have been revised in recent years. For example, a new checklist of the Brassicaceae family, the fifth-largest family in the country, was provided by German (2015). Taxonomic notes and checklists of *Aquilegia* L., *Stipa* L., and *Primula* L. were compiled by Erst et al. (2016), Zhao et al. (2019) and Baasanmunkh et al. (2020a), respectively. Recently, Troshkina (2021) revised and updated Geraniaceae in Mongolia. Baasanmunkh et al. (2021b) compiled a checklist of Orchidaceae, which included notes on their species richness and conservation status. The families Menyanthaceae and Nymphaeaceae were also revised by Baasanmunkh et al. (2022a). Additionally, some thorough regional floristic works were published: for the Khangai (Biazrov et al. 1989) and the Dzungarian Gobi regions (Baasanmunkh et al. 2021a). Moreover, an updated checklist of endemic plant species was recently provided by Baasanmunkh et al. (2021c), which comprises 102 taxa (95 species, 5 subspecies, and 2 varieties) from 43 genera and 19 families in the flora of Mongolia.

### ﻿New additions to the flora of Mongolia

Since Urgamal et al. (2014), 13 new species and one infraspecific taxon from Mongolia have been described as new to science (Nobis 2014; Erst et al. 2015, 2016; Kechaykin and Kutsev 2015; Yurtseva et al. 2016; Alexeeva 2018; Gundegmaa and Kechaykin 2018; Ovczinnikova 2019a, 2020; Pyak and Pyak 2019; Zhao et al. 2019; He et al. 2020; Pyak et al. 2020). Many new records of vascular plants have also been reported (Nobis et al. 2014, 2019a; Doronkin et al. 2015; Urgamal et al. 2016, 2019; Baasanmunkh et al. 2019a, b, c, 2020a, b, 2021b, d; Bazarragchaa et al. 2019; Erst et al. 2019; Ovczinnikova 2019b; Knyazev 2020; Shiga et al. 2020; Yano et al. 2021), including five genera new to the country, i.e. *Matthiola* W.T.Aiton, Brassicaceae (German 2015), *Onoclea* L., Onocleaceae (Doronkin et al. 2015), *Aldrovanda* L., Droseraceae (Shiga et al. 2020), *Hydrilla* L., Hydrocharitaceae (Shiga et al. 2020), and *Arctium* L., Asteraceae (Javzandolgor et al. 2021). Additionally, some genera previously listed by Urgamal et al. (2014) were omitted from Mongolian flora based on recent studies. In particular, the genus *Epipactis* Zinn. (Orchidaceae), for example, had two species that have been proven absent in the country due to the inaccurate location written on the herbarium specimens (Baasanmunkh et al. 2021b). On the other hand, some genera were not listed in Urgamal et al. (2014); for example, the genus *Phyllodoce* Salisb. (Ericaceae) was found in northern Mongolia by Oyunmaa and de Priest (2011). Furthermore, representatives of some genera, which are listed in the flora of Mongolia (Gubanov 1996; Urgamal et al. 2014), have been revised in recent studies (Podlech et al. 2013; Sukhorukov et al. 2013, 2019; Wang et al. 2014; Global Carex Group 2015; Duan et al. 2016; Drew et al. 2017; Kosachev 2017; Moore and Dillenberger 2017; Nosov et al. 2017; Pimenov 2017; Wiegleb et al. 2017; Boltenkov 2018; Gillespie et al. 2018; Madhani et al. 2018; Sinitsyna et al. 2018; Zhang et al. 2018; Barberá et al. 2019, 2020; Nobis et al. 2019b; Sramkó et al. 2019; Akan et al. 2020; Esput 2020; Friesen et al. 2020; Murakami et al. 2020; Nesom 2020; Ren et al. 2020; Zaika et al. 2020; Al-Shehbaz 2021; Al-Shehbaz et al. 2021; Liu et al. 2021).

### ﻿Necessity to update the list of flora of Mongolia

In 2016, the orders and families of flowering plants were updated by the APG IV (2016). Similarly, a new classification of ferns and lycophytes was provided for the first time (PPG I 2016). Given the recent updates to the international plant classification system, as well as a number of recent publications that identify new species and records and their distribution in Mongolia, there is a pressing need to revise and provide an updated list of the floristic diversity. Therefore, our study aims to present a thoroughly revised checklist of Mongolian native vascular flora that comprises the up-to-date names of all species, genera, and families, by conducting comparisons to the latest checklist, Conspectus of Vascular Plants of Mongolia by Urgamal et al. (2014) and earlier studies by Grubov (1982) and Gubanov (1996).

## ﻿Materials and methods

The systematic order and taxonomic circumscription of the families is based on the following classifications: Ferns and Fern Allies by PPG I (2016), Gymnosperms by Christenhusz et al. (2011), and Angiosperms by APG IV (2016). The names of accepted genera and species mostly follow Govaerts et al. (2021), which is currently maintained by Plants of the World Online (POWO 2021). Additionally, we reference recently published taxonomic revisions of certain families and genera. The authorship of species, genera and families is given after the International Plant Names Index (IPNI 2021); each species is provided with the author and respective publication as a reference. The name changes and most common synonyms, compared to the previous checklist, are also provided. Species endemism is given after Baasanmunkh et al. (2021c). Sub-endemic species are those that have also been found in at least one other country outside Mongolia, such as China, Kazakhstan, or Russia. For each species, we examined representative occurrence records based on the Global Biodiversity Information Facility (GBIF 2021, https://www.gbif.org/). We also compiled the phytogeographical regional distribution of all species, because species distribution is important information for species identification. The main herbaria for Mongolian flora (ALTB, LE, MW, MHA, NS, NSK, OSBU, TK, UBA, and UBU; acronyms follow Thiers 2021), Virtual Guide to the Flora of Mongolia (Rilke et al. 2012; Zemmrich et al. 2013; https://floragreif.uni-greifswald.de/floragreif/), and all literature data for the species’ regional distribution, have been checked and studied. The regional distribution of the taxa mostly follows Gubanov (1996), Urgamal et al. (2014), German (2015), and Baasanmunkh et al. (2021a). In addition to these sources, we used a revision of Baasanmunkh et al. (2022b), where numerous species were added or excluded from some phytogeographical regions. Lastly, the checklist comprises only native species, thus non-native taxa are not included and should be adressed in a future publication.

## ﻿Results

**Figure 1.**
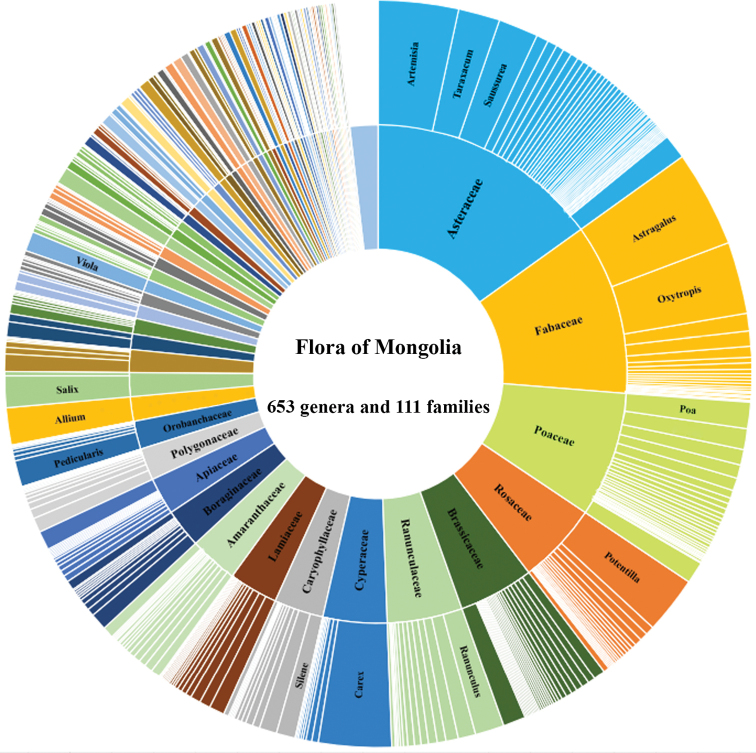
Species richness of genera (≥ 24 taxa) and families (≥ 57 taxa) of vascular flora of Mongolia. The names of only the most species-rich genera and families are shown.

The current checklist comprises 3,042 native vascular plant taxa (including 2,891 species, 116 subspecies, 29 varieties, and 12 nothospecies), belonging to 653 genera and 111 families (Table 1, Fig. 1). The updated checklist is divided into four major taxonomic groups: lycophytes (2 families and 4 genera), ferns and fern allies (12 families and 17 genera), gymnosperms (3 families and 6 genera), and angiosperms (94 families and 626 genera) (see Table 1 for detailed numbers of taxa). Among these, angiosperms comprise 2,979 taxa, which constitute 97% of Mongolian flora (Table 1). We cross-checked the occurrence of each taxon using GBIF (2021), which includes occurrence data for 2,249 taxa (73% of Mongolian flora).

**Table 1.** Number of native Mongolia vascular plant taxa in each taxonomic group.

Major taxonomic groups	Family	Genus	Taxon
Lycophytes	2	4	7
Ferns and fern allies	12	17	41
Gymnosperms	3	6	21
Angiosperms	94	627	2,972
**Total**	**111**	**653**	**3,041**

There are 14 families with a high species richness (≥ 9 genera and ≥ 57 taxa): Asteraceae (85 genera and 456 taxa), Fabaceae (24 and 328), Poaceae (58 and 229), Rosaceae (28 and 168), Ranunculaceae (20 and 156), Brassicaceae (51 and 138), Cyperaceae (10 and 130), Lamiaceae (22 and 103), Amaranthaceae (30 and 94), Caryophyllaceae (20 and 97), Boraginaceae (24 and 78), Apiaceae (36 and 66), Polygonaceae (11 and 63), and Orobanchaceae (9 and 57) (Fig. 1). The remaining 97 families comprise a smaller set of taxa. At the genus level, 14 genera represent a high species richness (≥ 24 taxa): *Astragalus* L. (127 taxa), *Artemisia* L. (103), *Carex* L. (99) *Oxytropis* DC. (97), *Potentilla* L. (75), *Saussurea* DC. (55), *Taraxacum* F.H.Wigg. (53), *Allium* L. (50), *Salix* L. (42), *Ranunculus* L. (41), *Pedicularis* L. (36), *Poa* L. (28), *Viola* L. (27), and *Silene* L. (24) which is shown in Fig. 1.

In this study, a total of 275 sub-endemic taxa are provided which account for 9% of the total species of flora of the country. Among these, Fabaceae (74 taxa) show the highest number of sub-endemic taxa along with Asteraceae (60 taxa), Brassicaceae (23 taxa), Poaceae (18 taxa), and Amaranthaceae (9 taxa). The highest number of sub-endemic taxa were found in the Mongolian Altai (114 taxa) followed by Khangai (87 taxa), Gobi-Altai (76 taxa), Khovd (68 taxa), Khuvsgul (63 taxa), and the Depression of Great Lakes (60 taxa). The remaining ten regions have between 24 and 58 sub-endemic taxa (Table 2).

**Table 2.** The species richness of the total, endemic, and sub-endemic vascular plants of each phytogeographical region of Mongolia.

Region number	Name of the phytogeographical regions	Taxon	Endemic	Sub-endemic
1	Khuvsgul	1,054	7	63
2	Khentei	1,236	6	48
3	Khangai	1,514	27	87
4	Mongolian Dauria	1,198	6	54
5	Foothills of Great Khyangan	793	3	24
6	Khovd	1,011	10	68
7	Mongolian Altai	over 1400	47	114
8	Middle Khalkh	777	4	49
9	East Mongolia	952	3	50
10	Depression of Great Lakes	882	16	60
11	Valley of Lakes	466	5	39
12	East Gobi	462	2	57
13	Gobi Altai	865	16	76
14	Dzungarian Gobi	913	20	58
15	Transaltai Gobi	356	8	40
16	Alashan Gobi	262	2	43

**Figure 2.**
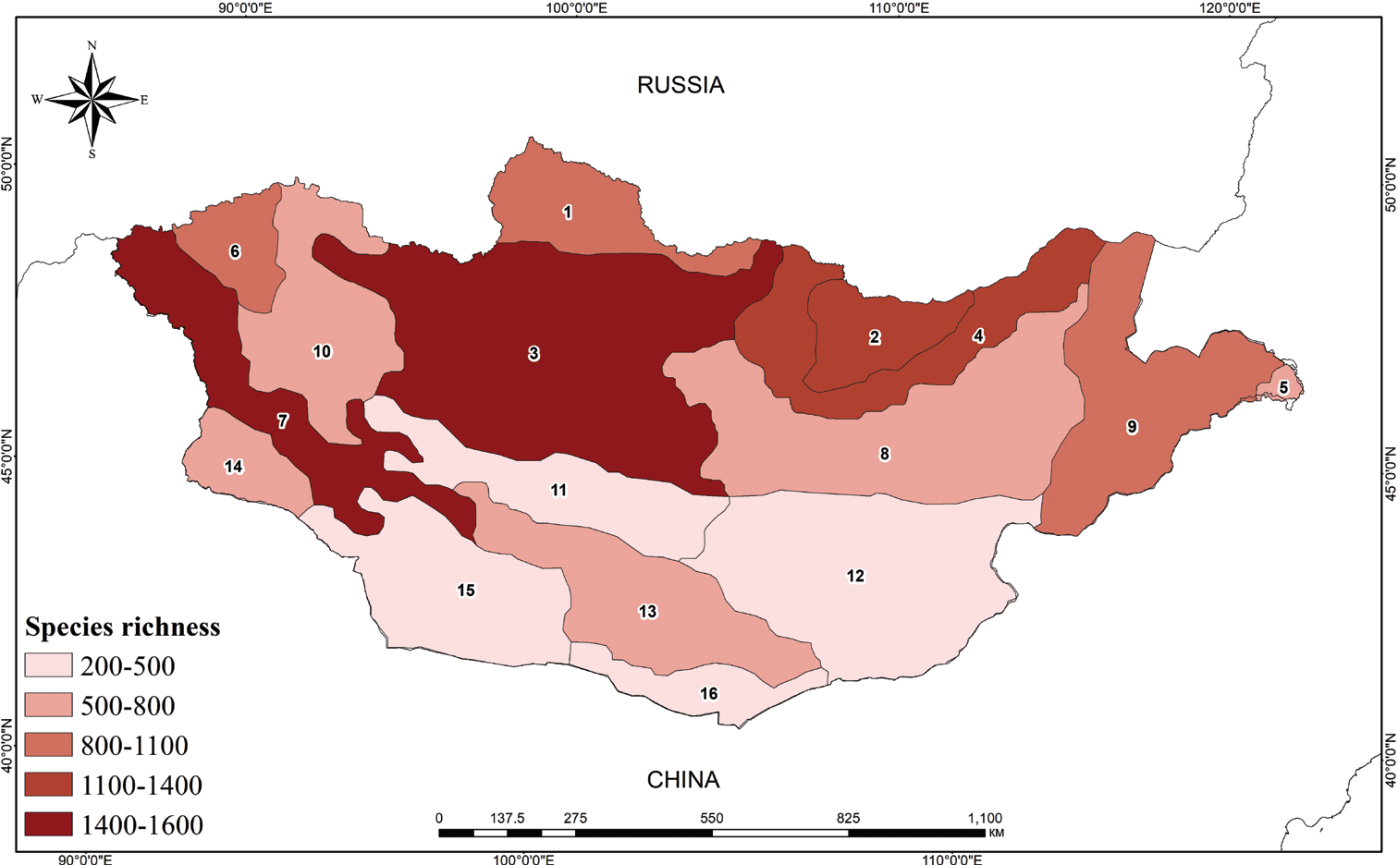
The species richness of native vascular plants of each phytogeographical region in Mongolia (**1** Khuvsgul **2** Khentei **3** Khangai **4** Mongolian Dauria **5** Foothills of Great Khyangan **6** Khovd **7** Mongolian Altai **8** Middle Khalkh **9** East Mongolia **10** Depression of Great Lakes **11** Valley of Lakes **12** East Gobi **13** Gobi Altai **14** Dzungarian Gobi **15** Transaltai Gobi **16** Alashan Gobi).

Based on the species distribution across the 16 phytogeographical regions, six regions with high species richness (over 1,000 taxa) were identified: the Mongolian Altai (more than 1,400 taxa), Khangai (1,514 taxa), Khentei (1,236 taxa), Mongolian Dauria (1,118 taxa), Khuvsgul (1,054 taxa), and Khovd (1,011 taxa). The remaining ten regions have between 262 and 952 taxa (Table 2, Fig. 2).

In this study, we primarily excluded non-native species, including archeophytes that were listed in the previous checklist of Mongolia by Urgamal et al. (2014). As a result, 59 plant taxa found in Mongolia are considered non-native, including the families Cannabaceae (*Cannabissativa* L.) and Portulacaceae (*Portulacaoleracea* L.). Several taxa were found to be archeophytes, which were introduced in “ancient” times and became naturalized as part of the native flora (a full list is given in Table 3).

**Table 3.** List of non-native taxa that were included in the previous checklist of Mongolian flora but are excluded from this checklist.

No	Taxon name	No	Taxon name
	Amaranthaceae		Fabaceae
1	*Amaranthusalbus* L.	30	*Lathyrusoleraceus* Lam.
2	*Amaranthusblitoides* S.Watson	31	*Lathyrussativus* L.
3	*Amaranthuscruentus* L.	32	*Lotuscorniculatus* L.
4	*Amaranthusretroflexus* L.	33	*Medicagosativa* L.
	Apiaceae	34	*Melilotusalbus* Medik.
5	*Anethumgraveolens* L.	35	*Ornithopusperpusillus* L.
6	*Eryngiumplanum* L.	36	*Trigonellacaerulea* (L.) Ser.
7	*Pastinacasativa* L.	37	*Viciaangustifolia* L.
	Asteraceae	38	*Viciafaba* L.
8	*Cichoriumintybus* L.	39	*Viciasativa* L.
9	*Sonchusoleraceus* L.	40	*Viciasepium* L.
10	*Xanthiumorientale* L.		Malvaceae
11	*Xanthiumstrumarium* L.	41	*Hibiscustrionum* L.
	Brassicaceae		Plantaginaceae
12	*Berteroaincana* (L.) DC.	42	*Veronicaperegrina* L.
13	*Brassicacampestris* L.		Poaceae
14	*Brassicajuncea* (L.) Czern.	43	*Avenafatua* L.
15	*Buniasorientalis* L.	44	*Avenasativa* L.
16	*Camelinacaucasica* (Sinskaya) Vassilcz.	45	*Cenchrusamericanus* (L.) Morrone
17	*Camelinasativa* (L.) Crantz	46	*Chlorisvirgata* Sw.
18	*Erucasativa* Mill.	47	*Dactylisglomerata* L.
19	*Guentherapersica* (Boiss. & Hohen.) D.A.German	48	*Hordeumaegiceras* Royle ex Walp.
20	*Nesliapaniculata* (L.) Desv.	49	*Hordeumdistichon* L.
21	*Raphanusraphanistrum* L.	50	*Hordeumvulgare* L.
22	*Sinapisarvensis* L.	51	*Panicummiliaceum* L.
23	*Sisymbriumaltissimum* L.	52	Panicummiliaceumsubsp.ruderale (Kitag.) Tzvelev
24	*Sisymbriumvolgense* M.Bieb.	53	*Secalecereale* L.
	Cannabaceae	54	*Setariapumila* Roem. & Schult.
25	*Cannabissativa* L.	55	*Setariaviridis* (L.) P.Beauv.
	Caryophyllaceae	56	*Triticumaestivum* L.
26	*Agrostemmagithago* L.		Polygonaceae
27	*Gypsophilavaccaria* (L.) Sm.	57	*Fagopyrumesculentum* Moench
28	*Silenebanksia* (Meerb.) Mabb.	58	*Fagopyrumtataricum* (L.) Gaertn.
	Convolvulaceae		Portulacaceae
29	*Calystegiasilvatica* (Kit.) Griseb.	59	*Portulacaoleracea* L.

## ﻿Discussion

We revised the checklist of Mongolian vascular plants provided by Urgamal et al. (2014), which included 3,127 taxa, belonging to 683 genera and 112 families. Because the list comprised both native and non-native taxa, we first sorted non-native taxa out to make our study comparable. There were 3,069 native taxa from 682 genera and 110 families listed by Urgamal et al (2014) and the current checklist has been shortened and comprises 3,041 native vascular plant taxa from 653 genera and 111 families. Since Grubov (1955) provided the first checklist, more than 1,150 species have been added to the flora of Mongolia. In this study, the family Brassicaceae is based upon the work of German (2015), who recognized 141 species from 59 genera which significantly differs from the 160 species and 61 genera provided by Urgamal et al. (2014); this is because of misidentification of species and records from outside Mongolia being included in the latter publication. Since Urgamal et al. (2014), 14 taxa have been described as new to science based on morphological evidence, the majority of which are from Boraginaceae (Suppl. material 1: Appendix 1). Furthermore, 79 taxa have recently been added to the flora of Mongolia (Suppl. material 1: Appendix 1). On the other hand, many taxa are synonymized and/or the names and status of numerous taxa were changed based on our critical revisions (list of the synonymized taxa is provided Suppl. material 2: Appendix 2). In particular, accepted names of three families, 21 genera, and 232 taxa have been changed/synonymized. Moreover, 21 taxa listed in the Urgamal et al (2014) were absent from Mongolia based on our extensive research which is given in Suppl. material 2: Appendix 2 (Pimenov 2017; Ovczinnikova 2019a; Nesom 2020; Baasanmunkh et al. 2021b, 2022a). Due to this high number of synonymization, even after dozens of new species have been discovered in Mongolian flora, change in absolute species numbers appeared to be minor.

Previous checklists of vascular plants in Mongolia (Gubanov 1996; Urgamal et al. 2014), listed both native and non-native taxa; however, there were no specific remarks for non-native taxa. Recently, many researchers have published checklists of non-native plants, mainly in Europe and East Asia, for example, concerning China (Xu et al. 2012), Italy (Galasso et al. 2018), South Korea (Korea National Arboretum 2021) as well as the Russian Far East and Siberia (Ebel et al. 2014; Vinogradova et al. 2020). These works aim to increas awareness about invasive species and strengthen their biosecurity regulations. This level of detail is important because invasions have complex and often immense long-term direct and indirect effects on native natural communities (Pagad et al. 2018; van Kleunen et al. 2019; Pyšek et al. 2020). In many Central Asian countries, there is no separate checklist for non-native plants species (Sennikov and Lazkov 2021). The first checklist of alien species was recently published for Kyrgyzstan by Sennikov et al. (2021). In Mongolia, approximately 35 non-native plants taxa are recognized in GBIF (Munkhnast et al. 2020), but this list has not critically revised all non-native plants species of the country. In this study, we primarily excluded non-native species that were listed in the previous checklist of Mongolia, including two families Cannabaceae (*Cannabissativa* L.) and Portulacaceae (*Portulacaoleracea* L.) and 57 other taxa (Table 3).

Mongolia has a relative small number of endemic vascular plants, with 102 taxa belonging to 43 genera and 19 families, accounting for only about 3% of the country’s total flora (Baasanmunkh et al. 2021c). Notably, the Mongolian Altai and Khangai regions harbor over 70% of the total endemics and sub-endemics of Mongolia, which reflects their high species richness (Fig. 2; Table 2). This could be due to the diverse habitats along the Altai mountains and the large expanse of forest and mountain-steppe in both regions. Regional distribution of new species, new records, and recently revised genera and families, were provided based on literature. It is important to highlight that the study does not entirely revise regional distribution of each taxon. Nonetheless, we have been working on the grid distribution map of vascular plants (see Baasanmunkh et al. 2021b, 2022a) since 2020 based on critical revision of herbarium specimens, literature, and our own field observation data.

Both of the online databases are allowing researchers to collaborate and revise Mongolian taxa more readily and will continue to improve the documentation of Mongolia’s flora. To date, a total of 2,249 taxa (ca. 73% of the flora) have been deposited in the database of GBIF (2021). Furthermore, the data for 1,249 species (including herbarium specimens and/or images of living plants) are at least partially available in the database of the Virtual Guide to the Flora of Mongolia (Rilke et al. 2012; Zemmrich et al. 2013; https://floragreif.uni-greifswald.de/floragreif/). Moreover, approximately 19,300 images of 1,780 taxa have been observed as part of citizen science contributions to the “Flora of Mongolia” project on the iNaturalist platform (https://www.inaturalist.org/projects/flora-of-mongolia?tab=observations), which was established on January 2019.

In this study, we checked more than 70 works published since 2013 that have revised the flora of Mongolia, and provided respective references for each species in our checklist. We reviewed the species status of all vascular flora of Mongolia and made critical changes by adding, synonymizing, and excluding taxa; this work resulted in 265 fewer taxa compared to Urgamal et al. (2014). We believe that our revised checklist serves as an essential background and reference not only for scientists and students, but also for local government administrations and protected areas, for the conservation of Mongolian flora. Having an updated checklist allows researchers and communities to monitor plants as climate and land use changes, and population size and herding pressures increase. We recommend more research be conducted on regional flora, as well as comprehensive revisions of species distribution based on herbarium collections for phytogeographical regions and taxonomic revision of doubtful taxa.

## ﻿Annotated checklist of native vascular flora in Mongolia.

The families in the checklist are alphabetically ordered and, within them, the genera, species, and subspecies are alphabetically listed. The currently accepted names are highlighted in bold italics. The most common synonyms (previously used in Urgamal et al. (2014)) and the species’ distribution in phytogeographical regions are provided here. Symbols used in the checklist include endemic [E] and sub-endemic [SE].

**I Lycophytes**

1. Lycopodiaceae P.Beauv. (3 genera and 5 species)

*Diphasiastrumalpinum* (L.) Holub [1, 2]

*Diphasiastrumcomplanatum* (L.) Holub [1]

*Huperziaselago* (L.) Bernh. [1, 2]

*Lycopodiumannotinum* L. [1, 2]

*Lycopodiumclavatum* L. [2]

**2. Selaginellaceae** Willk. (1 genus and 2 species)

*Selaginellaborealis* (Kaulf.) Spring [1, 3, 4, 8]

*Selaginellasanguinolenta* (L.) Spring [1, 2, 3, 4, 8]

**II Ferns and fern allies**

**3. Aspleniaceae** Newman (1 genus and 5 species)

*Aspleniumaltajense* (Kom.) Grubov [1, 3, 4, 7, 10, 13]

*Aspleniumruprechtii* Sa.Kurata [2, 3, 4]

*Aspleniumruta-muraria* L. [7, 14]

*Aspleniumseptentrionale* (L.) Hoffm. [7, 8, 10, 14]

*Aspleniumyunnanense* Franch. [7, 13]

**4. Athyriaceae** Alston (2 genera and 4 species)

*Athyriumfilix-femina* (L.) Roth [1–5]

*Athyriummonomachi* Kom. [2, 3, 4, 5]

*Athyriumsinense* Rupr. [3, 5, 9]

*Diplaziumsibiricum* (Turcz.) Sa.Kurata [1–5]

**5. Cystopteridaceae** Shmakov (2 genera and 4 species)

*Cystopterisfragilis* (L.) Bernh. [1–10, 13, 14, 15]

*Cystopterissudetica* A.Braun & Milde [2]

*Gymnocarpiumdryopteris* Newman [2, 4]

*Gymnocarpiumjessoense* (Koidz.) Koidz. [1, 2, 3, 4, 5, 8]

**6. Dennstaedtiaceae** Losty (1 genus and 1 species)

*Pteridiumaquilinum* (L.) Kuhn [2, 3, 4, 5]

**7. Dryopteridaceae** Herter (1 genus and 3 species)

*Dryopterisdilatata* (Hoffm.) A.Gray [2, 5]

*Dryopterisexpansa* (C.Presl) Fraser-Jenk. & Jermy [2, 5]

*Dryopterisfragrans* (L.) Schott [2, 3, 4, 5, 7, 10]

**8. Equisetaceae** Michx. (1 genus and 9 species)

*Equisetumarvense* L. [1–10, 14]

*Equisetumfluviatile* L. [1–10, 14]

*Equisetumhyemale* L. [3, 4, 5]

*Equisetumpalustre* L. [1–10, 14]

*Equisetumpratense* Ehrh. [1–7, 9, 10]

*Equisetumramosissimum* Desf. [14]

*Equisetumscirpoides* Michx. [1, 2, 3, 4, 6]

*Equisetumsylvaticum* L. [1–5, 8, 9]

*Equisetumvariegatum* Schleich. [1, 4]

**9. Onocleaceae** Pic.Serm. (2 genera and 2 species)

*Matteucciastruthiopteris* (L.) Tod. [2, 4]

*Onocleasensibilis* L. [2]

**10. Ophioglossaceae** Martinov (1 genus and 2 species)

*Botrychiumlanceolatum* (Gmel.) Ångstr. [3]

*Botrychiumlunaria* (L.) Sw. [1–7, 9]

**11. Polypodiaceae** J.Presl. & C.Presl (2 genera and 2 species)

*Lepisorusclathratus* Ching [13]

*Polypodiumvirginianum* L. [1–5, 8]

**12. Pteridaceae** E.D.M.Kirchn. (2 genera and 2 species)

*Cheilanthesargentea* (S.G.Gmel.) Kunze [1–9, 12, 13]

*Cryptogrammastelleri* (S.G.Gmel.) Prantl [1]

**13. Thelypteridaceae** Ching (1 genus and 1 species)

*Phegopterisconnectilis* (Michx.) Watt [2, 4]

**14. Woodsiaceae** Herter (1 genus and 6 species)

*Woodsiacalcarea* (Fomin) Shmakov [1, 3, 4]

*Woodsiaglabella* R.Br. [1, 7, 10]

*Woodsiaheterophylla* (Turcz.) Shmakov [1]

*Woodsiailvensis* (L.) R.Br. [= *Woodsiaacuminata* (Fomin) Sipliv.] [1–9, 13]

*Woodsiapseudopolystichoides* (Fomin) Kiselev & Shmakov [5]

*Woodsiasubcordata* Turcz. [4, 5, 9]

**III Gymnosperms**

**15. Cupressaceae** Gray (1 genus and 4 taxa)

*Juniperuscommunis* L. [1, 2, 3, 4, 6, 7]

*Juniperuspseudosabina* Fisch. & C.A.Mey. [1–4, 7, 8, 13]

Juniperussabinavar.davurica (Pall.) Farjon [= *Juniperusdavurica* Pall.] [2]

JuniperussabinaL.var.sabina [2–4, 6–8, 10, 11, 13, 14]

**16. Ephedraceae** Dumort. (1 genus and 9 species)

*Ephedradahurica* Turcz. [= Ephedrasinicasubsp.dahurica (Turcz.) Galanin] [2, 3, 8, 9, 12]

*Ephedraequisetina* Bunge [3, 6–9, 12–16]

*Ephedrafedtschenkoi* Paulsen [2, 3, 4, 7]

*Ephedraglauca* Regel [12, 14, 15]

*Ephedraintermedia* Schrenk & C.A.Mey. [7, 14, 15]

*Ephedralomatolepis* Schrenk [12, 14, 15]

*Ephedramonosperma* J.G.Gmel. [1–8, 10, 12–14]

*Ephedraprzewalskii* Stapf [6, 7, 10–16]

*Ephedrasinica* Stapf [2–5, 7–15]

**17. Pinaceae** Spreng. (4 genera and 8 species)

*Abiessibirica* Ledeb. [1, 2]

*Larixczekanowskii* Szafer [4]

*Larixgmelinii* (Rupr.) Kuzen. [≡ *Abiesgmelinii* Rupr.] [2, 4]

*Larixsibirica* Ledeb. [1–4, 6–8, 10, 14]

*Piceaobovata* Ledeb. [1, 2, 3, 6, 7]

*Pinuspumila* (Pall.) Regel [2]

*Pinussibirica* Du Tour [1, 2, 3, 6, 7]

*Pinussylvestris* L. [1–5, 8, 9]

**IV Angiosperms**

**18. Acoraceae** Martinov (1 genus and 1 species)

*Acoruscalamus* L. [1, 3, 4, 5, 8, 9]

**19. Adoxaceae** E.Mey. [including Viburnaceae Raf.] (3 genera and 6 species)

*Adoxamoschatellina* L. [1–3, 5–7, 13]

*Sambucussibirica* Nakai [3, 6]

*Sambucuswilliamsii* Hance [= *Sambucusmanshurica* Kitag.] [1, 2, 3, 4, 5, 9]

*Viburnumburejaeticum* Regel & Herder [5]

*Viburnummongolicum* Rehder [= *Loniceramongolica* Pall.] [4, 5, 8, 9]

*Viburnumsargentii* Koehne [5]

**20. Alismataceae** Vent. (2 genera and 4 species)

*Alismagramineum* Lej. [5, 7, 8, 10, 11, 14]

*Alismaplantago-aquatica* L. [1–5, 8–10, 14]

*Sagittarianatans* Pall. [4, 9, 10, 14]

*Sagittariatrifolia* L. [1, 4, 5, 9, 14]

**21. Amaranthaceae** Juss. [including Chenopodiaceae Vent.] (34 genera and 94 taxa)

*Agriophyllumpungens* (Vahl) Link [= *Agriophyllumsquarrosum* Moq.] [6–16]

*Anabasisaphylla* L. [7, 14]

*Anabasisbrevifolia* C.A.Mey. [3, 6–8, 10–16]

*Anabasiselatior* (C.A.Mey.) Schischk. [14]

*Anabasiseriopoda* Paulsen [14]

*Anabasispelliotii* Danguy [14]

*Anabasissalsa* Paulsen [14]

*Anabasistruncata* Bunge [7, 14]

*Atriplexaltaica* Sukhor. [7]

*Atriplexcana* C.A.Mey. [14]

*Atriplexfera* (L.) Bunge [1, 3, 4, 8–10, 12, 13]

*Atriplexlaevis* C.A.Mey. [3, 4, 8–11, 13–15]

*Atriplexsibirica* L. [2, 3, 4, 6–16]

*Atriplextatarica* L. [7, 10, 14]

*Axyrisamaranthoides* L. [2–5, 8, 9, 13]

*Axyrishybrida* L. [2–5, 7–10, 12–14]

*Axyrisprostrata* L. [1–4, 6–10, 13, 14]

*Bassiahyssopifolia* (Pall.) Kuntze [6, 7, 10–14]

*Bassiaprostrata* (L.) Beck [≡ *Kochiaprostrata* (L.) Schrad.] [1–15]

*Bassiascoparia* (L.) A.J.Scott [≡ *Kochiascoparia* (L.) Schrad.] [2, 3, 4, 6–14]

*Blitumvirgatum* L. [≡ *Chenopodiumfoliosum* Asch.] [3, 4, 6, 7, 12–15]

Camphorosmamonspeliacasubsp.lessingii (Litv.) Aellen [6, 10, 14]

*Caroxylongemmascens* (Pall.) Tzvelev [≡ *Salsolagemmascens* Pall.] [10]

*Caroxylonpasserinum* (Bunge) Akhani & Roalson [≡ *Salsolapasserina* Bunge] [8, 10–16]

*Ceratocarpusarenarius* L. [6, 7, 10, 14]

*Chenopodiastrumhybridum* (L.) S.Fuentes, Uotila & Borsch [≡ *Chenopodiumhybridum* L.] [2, 3, 4, 5, 7–16]

*Chenopodiumacuminatum* Willd. [3–14, 16]

*Chenopodiumalbum* L. [1–16]

*Chenopodiumficifolium* Sm. [1, 9, 10, 11, 14]

*Chenopodiumfrutescens* C.A.Mey. [6, 7, 10]

*Chenopodiumiljinii* Golosk. [7, 10]

*Chenopodiumkaroi* Aellen [1–15]

*Chenopodiumnovopokrovskyanum* (Aellen) Uotila [≡ Chenopodiumalbumsubsp.novopokrovskyanum (Aellen) Uotila] [7]

*Chenopodiumstrictum* Roth [2, 4, 9, 14]

*Chenopodiumvulvaria* L. [3, 6–10, 13, 14]

*Climacopteraaffinis* (C.A.Mey.) Botsch. [≡ *Pyankoviaaffinis* (C.A.Mey.) Mosyakin & Roalson] [14]

*Climacopterasubcrassa* (Popov) Botsch. [14]

*Corispermumchinganicum* Iljin [1–12]

*Corispermumdeclinatum* Steph. ex Iljin [3, 4, 8]

*Corispermumelongatum* Bunge [≡ Corispermumstauntoniisubsp.elongatum (Bunge) Vorosch.] [10, 13]

*Corispermummongolicum* Iljin [3, 4, 7–16]

SE*Corispermumpatelliforme* Iljin [10, 13, 16]

SE*Corispermumtylocarpum* Hance [= *Corispermumgmelinii* Bunge] [12]

*Dysphaniabotrys* (L.) Mosyakin & Clemants [7, 14, 15]

*Gruboviadasyphylla* (Fisch. & C.A.Mey.) Freitag & G.Kadereit [≡ *Bassiadasyphylla* (Fisch. & C.A.Mey.) Kuntze, *Kochiadasyphylla* Fisch. & C.A.Mey.] [3–16]

*Gruboviakrylovii* (Litv.) Freitag & G.Kadereit [≡ *Kochiakrylovii* Litv.] [6, 7, 10–16]

*Gruboviamelanoptera* (Bunge) Freitag & G.Kadereit [≡ *Kochiamelanoptera* Bunge] [3, 6, 7, 10–16]

*Halocnemumstrobilaceum* (Pall.) M.Bieb. [14]

*Halogetonglomeratus* (M.Bieb.) C.A.Mey. [7, 10, 11, 14, 15]

*Halostachyscaspica* C.A.Mey. [14, 15]

*Haloxylonammodendron* (C.A.Mey.) Bunge [7, 10–16]

*Iljiniaregelii* (Bunge) Korovin [14, 15, 16]

*Kalidiumcaspicum* (L.) Ung.-Sternb. [10, 14]

*Kalidiumcuspidatum* (Ung.-Sternb.) Grubov [3, 8, 9, 12–16]

*Kalidiumfoliatum* Moq. [3, 6–16]

SE*Kalidiumgracile* Fenzl [3, 4, 8–16]

*Krascheninnikoviaceratoides* (L.) Gueldenst. [= *Krascheninnikoviaewersmanniana* (Stschegl.) Grubov] [1, 3, 4, 6–16]

*Micropeplisarachnoidea* (Moq.) Bunge [≡ *Halogetonarachnoides* Moq.] [4, 6–16]

SE*Nanophytongrubovii* U.P.Pratov [10]

SE*Nanophytonmongolicum* U.P.Pratov [7, 14]

*Oreosalsolaabrotanoides* (Bunge) Akhani [≡ *Salsolaabrotanoides* Bunge] [6–13]

*Oxybasischenopodioides* (L.) S.Fuentes, Uotila & Borsch [≡ *Chenopodiumchenopodioides* (L.) Aellen] [7, 10, 14]

*Oxybasisglauca* (L.) S.Fuentes, Uotila & Borsch [≡ *Chenopodiumglaucum* L.] [2–16]

*Oxybasisgubanovii* (Sukhor.) Sukhor. & Uotila [≡ *Chenopodiumgubanovii* Sukhor.] [10, 14]

*Oxybasisrubra* (L.) S.Fuentes, Uotila & Borsch [≡ *Chenopodiumrubrum* L.] [3, 7, 10, 14, 15]

*Oxybasisurbica* (L.) S.Fuentes, Uotila & Borsch [≡ *Chenopodiumurbicum* L.] [4, 10]

*Petrosimonialitvinowii* Korsh. [10]

*Petrosimoniasibirica* Bunge [14]

SE*Salicorniaaltaica* Lomon. [≡ Salicorniaperennanssubsp.altaica (Lomon.) G.Kadereit & Piirainen] [7]

*Salsolacollina* Pall. [≡ *Kalicollinum* (Pall.) Akhani & Roalson] [2–15]

SE*Salsolaikonnikovii* Iljin [≡ *Kaliikonnikovii* (Iljin) Akhani & Roalson] [7, 11, 12, 13]

*Salsolajacquemontii* Moq [≡ *Kalijacquemontii* (Moq.) Akhani & Roalson] [8, 13]

*Salsolalaricifolia* Litv. [12, 13, 16]

*Salsolamonoptera* Bunge [≡ *Kalimonopterum* (Bunge) Lomon.] [3, 6–13]

*Salsolapaulsenii* Litv. [≡ *Kalipaulsenii* (Litv.) Akhani & Roalson] [3, 7, 9–11, 14]

*Salsolarosacea* L. [7, 14]

*Salsolatragus* L. [2, 3, 4, 6–16]

*Sodafoliosa* (L.) Akhani [≡ *Salsolafoliosa* L. ≡ *Neocaspiafoliosa* (L.) Tzvelev] [14]

*Suaedaacuminata* (C.A.Mey). Moq. [6, 10, 14]

Suaedacorniculata(C.A.Mey.)Bungesubsp.corniculata [1, 3–16]

Suaedacorniculatasubsp.mongolica Lomon. & Freitag [3, 4, 7–11]

*Suaedaglauca* (C.A.Mey.) Bunge [4, 9, 16]

*Suaedaheterophylla* (Kar. & Kir.) Bunge [10–15]

*Suaedakossinskyi* Iljin [≡ *Bienertiakossinskyi* (Iljin) Tzvelev] [6, 7, 10, 13, 16]

*Suaedalinifolia* Pall. [10, 14]

*Suaedaprostrata* Pall. [= *Suaedamaritima* auct. non L.] [6, 9–13, 16]

*Suaedaprzewalskii* Bunge [≡ *Bienertiaprzewalskii* (Bunge) G.L.Chu] [10–13]

*Suaedasalsa* (L.) Pall. [8–13, 15, 16]

*Suaedasibirica* Lomon. & Freitag [3, 4, 8, 9, 10]

SE*Suaedatschujensis* Lomon. & Freitag [6, 7]

SE*Suaedatuvinica* Lomon. & Freitag [3, 6, 10]

*Sympegmaregelii* Bunge [7, 10–16]

*Teloxysaristata* (L.) Moq. [≡ *Chenopodiumaristatum* L. ≡ *Dysphaniaaristata* (L.) Mosyakin & Clemants] [3–13, 15, 16]

*Xylosalsolaarbuscula* (Pall.) Tzvelev [≡ *Salsolaarbuscula* Pall.] [7, 13–16]

**22. Amaryllidaceae** J.St.-Hil. (1 genus and 50 taxa)

Note: According to Sinitsyna et al. (2018), *Alliumspirale* is absent in Mongolia and *A.subangulatum* was found in southern Gobi by Friesen et al. (2020).

*Alliumaltaicum* Pall. [1–3, 6–8, 10, 13, 14]

*Alliumamphibolum* Ledeb. [1–4, 6, 7, 10, 13, 14]

*Alliumanisopodium* Ledeb. [2–13]

SE*Alliumaustrosibiricum* N.Friesen [3, 6, 7, 10, 14]

*Alliumbaicalense* Willd. [= Alliumsenescenssubsp.glaucum (Schrader) Dostál] [1, 3–5, 9, 10]

*Alliumbidentatum* Fisch. [1–6, 8–12, 14]

*Alliumburjaticum* N.Friesen [3, 4, 8]

*Alliumcarolinianum* Redouté [14]

*Alliumchamarense* M.M.Ivanova [1, 2, 3]

*Alliumclathratum* Ledeb. [3, 6, 7, 10, 11]

*Alliumcondensatum* Turcz. [5, 9]

*Alliumeduardi* Stearn [2, 3, 4, 6–16]

*Alliumflavidum* Ledeb. [1–4, 6, 7, 13, 14]

*Alliumgalanthum* Kar. & Kir. [7, 14]

*Alliumhymenorrhizum* Ledeb. [7, 14]

*Alliumkarelinii* Poljakov [7, 14]

*Alliumledebourianum* Schult. & Schult.f. [7]

*Alliumleucocephalum* Turcz. [1–4, 7–14, 16]

*Alliummacrostemon* Bunge [8, 9]

*Alliummalyschevii* N.Friesen [1, 2, 3]

*Alliummaximowiczii* Regel [2, 4, 5, 9]

*Alliummicrodictyon* Prokh. [1, 2, 3, 4]

*Alliummonadelphum* Turcz. [1, 2, 3, 6, 7]

*Alliummongolicum* Regel [3, 4, 6–16]

*Alliumneriniflorum* G.Don [4, 5, 9]

*Alliumobliquum* L. [7]

*Alliumoliganthum* Kar. & Kir. [6, 7, 10, 14]

*Alliumpallasii* Murray [14]

AlliumplatyspathumSchrenksubsp.platyspathum [3, 6, 7, 13, 14]

Alliumplatyspathumsubsp.amblyophyllum (Kar. & Kir.) N.Friesen [7, 13, 14]

*Alliumpolyrhizum* Turcz. [1, 2, 3, 4, 7–16]

*Alliumprostratum* Trev. [1–13]

SE*Alliumpumilum* Vved. [6, 7, 14]

*Alliumramosum* L. [1–13]

*Alliumrubens* Schrad. [6, 7, 14]

*Alliumschischkinii* Sobolevsk. [3, 6, 7, 10, 11, 13]

*Alliumschoenoprasum* L. [1–7, 10]

*Alliumschrenkii* Regel [= *Alliumbogdoicola* Regel] [3, 6, 7, 10, 13, 14]

*Alliumsenescens* L. [1–10, 13]

*Alliumsplendens* Willd. [1–5, 8, 9]

*Alliumspurium* G.Don [1, 2, 4, 5, 9]

*Alliumstellerianum* Willd. [1, 2, 3, 4]

*Alliumstrictum* Schrad. [1–10, 13, 14]

SE*Alliumsubangulatum* Regel [16]

*Alliumsubtilissimum* Ledeb. [3, 14]

*Alliumtenuissimum* L. [1–5, 7–9, 11–15]

*Alliumtuvinicum* (N.Friesen) N.Friesen [≡ Alliumstellerianumsubsp.tuvinicum N.Friesen] [3, 6, 7, 10]

SE*Alliumtytthocephalum* Schult.f. [4, 6, 7, 13]

SE*Alliumubsicola* Regel [6, 10, 14]

*Alliumvodopjanovae* N.Friesen [3, 4, 6–8, 10–15]

**23. Apiaceae** Lindl. (36 genera and 66 taxa)

*Aegopodiumalpestre* Ledeb. [1–5, 13]

*Angelicaczernaevia* (Fisch. & C.A.Mey.) Kitag. [5, 9]

*Angelicadahurica* (Hoffm.) Benth. & Hook.f. [≡ *dahurica* Hoffm.] [2, 3, 4, 5, 9]

*Angelicasaxatilis* Turcz. [≡ *Physolophiumsaxatile* (Turcz.) Turcz.] [2]

*Angelicasylvestris* L. [6, 7]

*Anthriscussylvestris* (L.) Hoffm. [1–10]

*Archangelicadecurrens* Ledeb. [≡ Angelicaarchangelicasubsp.decurrens (Ledeb.) Kuvaev] [1–4, 6, 7, 14]

*Aulacospermumanomalum* Ledeb. [6, 7]

*Bupleurumaureum* Fisch. [7]

*Bupleurumbicaule* Helm [= *Bupleurumpusillum* Krylov] [1–4, 6–13]

*Bupleurumdensiflorum* Rupr. [= *Bupleurummongolicum* V.M.Vinogr.] [7, 13, 14]

*Bupleurumkrylovianum* Schischk. [3, 7]

*Bupleurummultinerve* DC. [= *Bupleurumlongeinvolucratum* Krylov] [1–5, 7, 9, 11]

*Bupleurumscorzonerifolium* Willd. [1–6, 8, 9, 12, 13]

*Bupleurumsibiricum* Vest [2, 4, 8, 9]

*Carumburiaticum* Turcz. [1–6, 8, 9]

*Carumcarvi* L. [1–5, 7–10, 14]

*Cenolophiumdenudatum* (Hornem.) Tutin [3, 7, 10, 14]

*Cicutavirosa* L. [1–15]

*Cnidiumdauricum* (Jacq.) Turcz. [≡ *Laserpitiumdauricum* Jacq.] [2–10]

*Cnidiummonnieri* Cusson [4, 9]

SE*Conioselinumlongifolium* Turcz. [1, 2, 4, 7, 9, 10]

*Conioselinumtataricum* Hoffm. [= *Conioselinumvaginatum* (Spreng.) Thell.] [1, 2, 3, 4]

*Elwendiasetacea* (Schrenk) Pimenov & Kljuykov [≡ *Buniumsetaceum* (Schrenk) H.Wolff, ≡ *Carumsetaceum* Schrenk] [6, 7]

SE*Ferulabungeana* Kitag. [5, 8–16]

*Ferulacaspica* M.Bieb. [7, 14]

*Feruladissecta* Ledeb. [3, 6, 7, 10, 14]

*Feruladshaudshamyr* Korovin [= *Feruladubjanskyi* Korovin] [7, 14]

*Ferulaferulioides* (Steud.) Korovin [7]

*Ferulapotaninii* Korovin [14]

*Ferulasoongarica* Pall. [= *Ferulamongolica* (V.M.Vinogr. & Kamelin) V.M.Vinogr. & Kamelin] [3, 7, 10, 14, 15]

SE*Ferulopsishystrix* (Bunge) Pimenov [≡ *Peucedanumhystrix* Bunge] [2–4, 6–11, 13, 15]

SE*Haloselinumfalcaria* (Turcz.) Pimenov [≡ *Peucedanumfalcaria* Turcz.] [1, 3, 4, 6–8, 10, 11, 13–16]

*Hanseniamongholica* Turcz. [≡ *Ligusticummongholicum* (Turcz.) Krylov] [1, 2]

*Heracleumdissectum* Ledeb. [1–7, 9, 10, 11, 13]

*Heracleumsibiricum* L. [1, 2, 3, 9, 13]

*Kadeniasalina* (Turcz.) Lavrova & V.N.Tikhom. [≡ *Cnidiumsalinum* Liou] [2, 3, 4, 8–11, 13]

*Kitagawiabaicalensis* (Redow.) Pimenov [≡ *Peucedanumbaicalense* (Redow.) Koch] [1–8, 10]

*Kitagawiaterebinthacea* (Fisch.) Pimenov [≡ *Peucedanumterebinthaceum* (Fisch.) Ledeb.] [2, 4, 5, 9]

SE*Lithosciadiumkamelinii* (V.M.Vinogr.) Pimenov [≡ *Cnidiumkamelinii* V.M.Vinogr.] [7]

SE*Lithosciadiummulticaule* Turcz. [1, 3, 4, 6, 7, 13]

*Neogayasimplex* Meisn. [= *Pachypleurumalpinum* Ledeb.] [10]

*Oenantheaquatica* (L.) Poir. [= *Peucedanumsalinum* Pall.] [1–4, 6–10, 13]

*Ostericumtenuifolium* (Pall.) Y.C.Chu [= *Pachypleurumalpinum* Ledeb.] [1–4, 6, 7, 13, 14]

*Paraligusticumdiscolor* (Ledeb.) V.N.Tikhom. [7]

SE*Peucedanumpuberulum* Turcz. [2, 3, 6, 8, 13]

*Peucedanumvaginatum* Ledeb. [1–4, 6, 7, 8, 11, 13]

*Phlojodicarpussibiricus* Koso-Pol. [1–4, 7, 8, 9, 13]

*Phlojodicarpusvillosus* Turcz. [1, 2, 3, 6]

*Pimpinellathellungiana* H.Wolff [4, 5, 9]

*Pleurospermumuralense* Hoffm. [1–6, 8, 9]

*Prangosledebourii* Herrnst. & Heyn [7, 14]

*Sajanellamonstrosa* (Willd.) Soják [1, 2]

*Saposhnikoviadivaricata* (Turcz.) Schischk. [2–6, 8, 9]

*Schulziacrinita* (Pall.) Spreng. [1, 2, 3, 4, 6, 7]

*Seseliabolinii* (Korovin) Schischk. [≡ *Libanotisabolinii* (Korovin) Korovin] [7, 10, 11, 13]

*Seselibuchtormense* W.D.J.Koch [≡ *Libanotisbuchtormensis* (Fisch.) DC.] [7, 14]

*Seselicondensatum* Rchb.f. [≡ *Libanotiscondensata* (L.) Fisch.] [1–3, 6–8, 10, 14]

*Seselieriocarpum* B.Fedtsch. [≡ *Libanotiseriocarpa* Schrenk] [7, 10, 14]

*Seseliglabratum* Willd. [= *Libanotistenuifolia* DC.] [7]

SE*Seseligrubovii* V.M.Vinogr. & Sanchir [≡ *Libanotisgrubovii* (V.M.Vinogr. & Sanchir) M.L.Sheh & M.F.Watson] [7, 13, 14, 15]

*Seselimucronatum* (Schrenk) Pimenov & Sdobnina [14]

*Seseliseseloides* (Fisch. & C.A.Mey.) M.Hiroe [≡ *Libanotisseseloides* (Fisch. & C.A.Mey.) Turcz.] [1–7, 9]

*Siumsuave* Walter [1–10, 14]

*Sphallerocarpusgracilis* Koso-Pol. [1–4, 6–13]

*Stenocoeliumathamantoides* Ledeb. [≡ *Seseliathamantoides* (M.Bieb.) Beck] [6, 7]

**24. Apocynaceae** Juss. (3 genera and 10 taxa)

*Apocynumpictum* Schrenk [= *Apocynumhendersonii* Hook.f.] [7, 11, 14, 15]

*Apocynumvenetum* L. [≡ *Poacynumvenetum* (L.) Mavrodiev] [14]

Cynanchumacutumsubsp.sibiricum (Willd.) Rech.f. [10–16]

*Cynanchumbungei* Decne. [9]

*Cynanchumchinense* R.Br. [9, 12, 15, 16]

SE*Cynanchumgobicum* Grubov [= *Vincetoxicumlanceolatum* (Grubov) Grubov] [12–16]

*Cynanchummongolicum* Hemsl. [16]

*Cynanchumpurpureum* K.Schum. [1, 2, 4, 5, 8, 9, 12, 16]

*Vincetoxicummukdenense* Kitag. [= *Cynanchumpaniculatum* (Bunge) Kitag.] [4, 5, 9]

*Vincetoxicumsibiricum* (L.) Decne. [= *Cynanchumthesioides* K.Schum.] [2–16]

**25. Araceae** Juss. (2 genera and 4 species)

*Lemnaminor* L. [1, 3, 4, 7–11, 13]

*Lemnatrisulca* L. [1–5, 8–11]

*Lemnaturionifera* Landolt [9, 11]

*Spirodelapolyrhiza* (L.) Schleid. [5, 9]

**26. Asparagaceae** Juss. (5 genera and 19 species)

*Anemarrhenaasphodeloides* Bunge [5, 9]

*Asparagusbrachyphyllus* Turcz. [9]

SE*Asparagusburjaticus* Peschkova [4]

*Asparagusdauricus* Fisch. [2–6, 8, 9, 11, 12]

SE*Asparagusgobicus* Ivanova [7–16]

*Asparagusneglectus* Kar. & Kir. [14]

*Asparagusoligoclonos* Maxim. [5]

*Asparaguspallasii* Miscz. [7, 10, 11]

*Asparagusschoberioides* Kunth [5]

*Asparagustamariscinus* Ivanova [10, 14, 15, 16]

*Asparagustrichophyllus* Bunge [10, 12, 14–16]

*Convallariakeiskei* Miq. [2, 5]

*Maianthemumbifolium* (L.) F.W.Schmidt [1, 2, 3, 4, 5]

*Maianthemumdilatatum* (Alph.Wood) A.Nelson & J.F.Macbr. [3, 4, 5, 9]

Maianthemum×intermedium Vorosch. [5]

*Maianthemumtrifolium* (L.) Sloboda [2]

*Polygonatumhumile* Fisch. [4, 5]

*Polygonatumodoratum* (Mill.) Druce [1–5, 8, 9]

*Polygonatumsibiricum* Redouté [1–5, 8, 9, 12]

**27. Asphodelaceae** Juss. [including Xanthorrhoeaceae Dumort.] (1 genus and 2 taxa)

HemerocallislilioasphodelusL.var.lillioasphodelus [4, 5, 9]

Hemerocallislilioasphodelusvar.minor (Mill.) M.N.Tamura [≡ *Hemerocallisminor* Mill.] [1–5, 8, 9]

**28. Asteraceae** Bercht. & J.Presl (85 genera and 456 taxa)

Note: Some classifications of some genera of Asteraceae have changed after extnesive molecular investigations. For example, species of *Scorzonera* L. were split into several genera, and three of them are present in Mongolia: *Lipschitzia* Zaika, Sukhor. & N.Kilian, *Takhtajaniantha* Nazarova, and *Scorzonera* L. s.str. by Zaika et al. (2020). The taxonomic status of *Scorzoneracurvata*, *S.grubovii*, and *S.sinensis* is not resolved yet.

*Achilleaacuminata* Sch.Bip. [2, 4, 5, 9]

*Achilleaalpina* L. [1–6, 8–10]

*Achilleaasiatica* Serg. [1–10, 14]

*Achilleaimpatiens* L. [2, 3, 4]

*Achillealedebourii* Heimerl [3, 7, 8]

*Achilleamillefolium* L. [1, 2, 3, 4, 7]

*Achilleaptarmicoides* Maxim. [2, 4, 8, 9, 10]

*Achilleasergievskiana* Shaulo & Shmakov [7]

SE*Ajaniaachilleoides* Poljakov [3, 6–8, 10–13, 15, 16]

*Ajaniafruticulosa* (Ledeb.) Poljakov [3, 4, 6–16]

E *Ajaniagrubovii* Muldashev [≡ *Chrysanthemumgrubovii* (Muldashev) H.Ohashi & Yonek.] [7, 14]

*Ajaniatrifida* (Turcz.) Muldashev [≡ *Hippolytiatrifida* (Turcz.) Poljakov] [3, 6–9, 11–13, 16]

*Allardiatridactylites* Sch.Bip. [≡ *Waldheimiatridactylites* Kar. & Kir.] [1, 3, 6, 7, 13]

*Ancathiaigniaria* DC. [3, 7, 10, 14, 15]

*Antennariadioica* (L.) Gaertn. [1, 2, 3, 4, 7]

*Arctiumtomentosum* Mill. [4]

*Arctogerongramineum* (L.) DC. [1–5, 7, 8, 9]

Arnicaangustifoliasubsp.iljinii (Maguire) I.K.Ferguson [7]

*Artemisiaadamsii* Besser [2, 3, 4, 6–13]

SE*Artemisiaaksaiensis* Y.R.Ling [2, 6, 8, 12–14, 16]

*Artemisiaamoena* Poljakov [7, 12]

*Artemisiaanethifolia* Weber [2, 3, 4, 7–16]

*Artemisiaanethoides* Mattf. [8–16]

*Artemisiaannua* L. [2–4, 7–10, 12–16]

*Artemisiaargyi* H.Lév. & Vaniot [2, 4, 5, 7–9, 12, 13]

*Artemisiaargyrophylla* Ledeb. [1, 3, 6, 7, 13, 15]

E *Artemisiaassurgens* Filatova [≡ *Seriphidiumassurgens* (Filatova) K.Bremer & Humphries] [7, 11, 13–15]

*Artemisiaaurata* Kom. [2–5, 8, 9, 13]

*Artemisiabargusinensis* Spreng. [1, 2, 3, 4, 5]

SE*Artemisiablepharolepis* Bunge [11, 12, 13, 16]

*Artemisiaborealis* Pall. [1–4, 6, 7, 10, 13]

*Artemisiaborotalensis* Poljakov [≡ *Seriphidiumborotalense* (Poljakov) Ling & Y.R.Ling] [7, 14]

SE*Artemisiabrachyloba* Franch. [4, 8, 9]

*Artemisiabrachyphylla* Kitam. [5]

*Artemisiacaespitosa* Ledeb. [3, 4, 6–16]

*Artemisiacapillaris* Thunb. [2, 3, 4, 5, 8, 9]

*Artemisiacompacta* Fisch. [3, 6–8, 10–12, 14]

*Artemisiadahurica* (Turcz.) Poljakov [4]

E *Artemisiadavazamczii* Darijma & Kamelin [7, 10, 13, 15]

*Artemisiademissa* Krasch. [3, 7–16]

*Artemisiadepauperata* Krasch. [1–4, 6–8, 10, 11, 13, 14]

ArtemisiadesertorumSpreng.subsp.desertorum [2, 4, 5, 9, 13]

E Artemisiadesertorumsubsp.pseudojaponica Darijma & Kamelin [5]

SE*Artemisiadisjuncta* Krasch. [7, 13]

SE*Artemisiadolosa* Krasch. [1–9, 11, 13]

SEArtemisiadracunculusvar.changaica (Krasch.) Y.R.Ling [≡ *Artemisiachangaica* Krasch.] [1, 3, 7, 8, 10, 11, 13]

ArtemisiadracunculusL.var.dracunculus [1–15]

*Artemisiaeriopoda* Bunge [16]

Artemisiafeddeisubsp.arschantinica (Darijma) Gubanov & Kamelin [≡ *Artemisiaarschantinica* Darijma] [16]

ArtemisiafeddeiH.Lév. & Vaniotsubsp.feddei [5, 9]

*Artemisiafreyniana* (Pamp.) Krasch. [4–6, 8–10, 12, 13]

*Artemisiafrigida* Willd. [1–16]

SE*Artemisiagiraldii* Pamp. [4]

*Artemisiaglauca* Pall. [1–8, 10, 14]

SE*Artemisiaglobosa* Krasch. [6,–8, 10, 12–14]

SE*Artemisiaglobosoides* Ling & Y.R.Ling [9, 12]

ArtemisiagmeliniiWeb.var.gmelinii [2–13]

Artemisiagmeliniivar.messerschmidiana (Besser) Poljakov [2–5, 8, 9, 12, 13]

*Artemisiagracilescens* Krasch. & Iljin [7, 14, 15]

*Artemisiahalodendron* Turcz. [4, 5, 8, 9, 12, 16]

*Artemisiaheptapotamica* Poljakov [≡ *Seriphidiumheptapotamicum* (Poljakov) Ling & Y.R.Ling] [7, 14]

*Artemisiaimplicata* T.G.Leonova [16]

*Artemisiaintegrifolia* L. [1–5, 8, 9, 13]

*Artemisiaklementzae* Krasch. [= *Artemisiaxylorhiza* Krasch.] [3, 4, 7–13, 16]

*Artemisialaciniata* Willd. [1–5, 7–10, 12, 14]

SEArtemisialagocephalaFisch.var.lithophila (Turcz.) Y.R.Ling [1]

*Artemisialatifolia* Ledeb. [2, 4, 5, 9]

*Artemisiamacilenta* (Maxim.) Krasch. [2, 3, 4, 5, 9]

*Artemisiamacrantha* Ledeb. [1, 2, 3, 7]

*Artemisiamacrocephala* Jacquem. [1–16]

*Artemisiamanshurica* (Kom.) Kom. [2, 3, 4, 5, 8, 9]

*Artemisiamarschalliana* Spreng. [7]

*Artemisiamaximovicziana* Krasch. [4, 5, 9]

*Artemisiamedioxima* Krasch. [1, 2, 3, 4, 9]

*Artemisiamongolica* (Fisch.) Nakai [≡ Artemisiavulgarisvar.mongolica Fisch.] [1–15]

Artemisiamongolorumsubsp.gobicum Krasch. [≡ *Artemisiagobica* (Krasch.) Grubov] [3, 4, 6–16]

ArtemisiamongolorumKrasch.subsp.mongolorum [≡ *Seriphidiummongolorum* (Krasch.) Ling & Y.R.Ling] [3, 4, 6–16]

*Artemisianitrosa* Weber [3, 4, 8, 9]

Artemisiaobtusilobasubsp.altaiensis (Krasch.) Krasnob. [≡ *Artemisiaaltaiensis* Krasch.] [3, 6, 7]

ArtemisiaobtusilobaLedeb.subsp.obtusiloba [3, 6, 7, 10, 13, 14]

Artemisiaobtusilobavar.glabra Ledeb. [= *Artemisiaglabella* Kar. & Kir.] [3, 6, 10]

SE*Artemisiaordosica* Krasch. [7, 9, 10, 12–16]

SE*Artemisiaoxycephala* Kitag. [4, 5, 8, 9]

*Artemisiapalustris* L. [1–13]

*Artemisiapamirica* C.Winkl. [3, 6, 7, 10–13]

*Artemisiaphaeolepis* Krasch. [1–4, 6–9, 13, 14]

*Artemisiapubescens* Ledeb. [= *Artemisiacommutata* Besser] [1–4, 6–11, 13, 14]

*Artemisiapycnorrhiza* Ledeb. [1–4, 6–8, 10–14]

*Artemisiarubripes* Nakai [2, 3, 4, 5, 8, 9]

*Artemisiarupestris* L. [1–4, 6–8, 10, 14]

Artemisiarutifoliavar.altaica (Krylov) Krasch. [7]

Artemisiasacrorumvar.messerschmidtiana (Besser) Y.R.Ling [2–5, 8, 9, 12, 13]

*Artemisiasaissanica* (Krasch.) Filatova [7, 10, 14]

*Artemisiasantolinifolia* Turcz. [= Artemisiasantolinifoliasubsp.stepposa Darijma] [2, 3, 6–15]

*Artemisiaschischkinii* Krasch. [6, 7, 10, 14]

*Artemisiaschrenkiana* Ledeb. [3, 6, 10, 14]

*Artemisiascoparia* Waldst. & Kit. [2–12]

*Artemisiaselengensis* Turcz. [3, 4, 5, 9]

*Artemisiasericea* Weber [1, 2, 3, 4, 5, 8]

*Artemisiasieversiana* Ehrh. [1–16]

SE*Artemisiasphaerocephala* Krasch. [3, 10–16]

*Artemisiastolonifera* (Maxim.) Kom. [3, 7, 10, 13–16]

SE*Artemisiasubchrysolepis* Filatova [≡ *Seriphidiumsubchrysolepis* (Filatova) K.Bremer & Humphries] [7, 14]

*Artemisiasubdigitata* Mattf. [≡ Artemisiadubiavar.subdigitata (Mattf.) Y.R.Ling] [3, 4, 7, 10, 12–16]

*Artemisiasublessingiana* Krasch. [= *Seriphidiumgorjaevii* (Poljak.) Y.R.Ling] [14]

*Artemisiasubulata* Nakai [1, 5, 9]

*Artemisiasucculenta* Ledeb. [7]

*Artemisiasylvatica* Maxim. [4, 5, 9, 10, 15]

*Artemisiatanacetifolia* L. [1–10, 14]

*Artemisiaterrae-albae* Krasch. [7, 14, 15]

*Artemisiatomentella* Trautv. [1, 3, 6, 10–12, 14]

*Artemisiatournefortiana* Rchb. [7, 9, 12, 14]

SE*Artemisiatransbaicalensis* T.G.Leonova [1, 3]

*Artemisiaumbrosa* (Besser) Turcz. [4, 5, 9]

*Artemisiavestita* Wall. [13]

*Artemisiaviridis* Willd. [6, 7, 14]

Artemisiavulgarissubsp.vulgaris L. [2, 3]

E Artemisiavulgarissubsp.inundata Darijma [= *Artemisiasuperba* Pamp.] [1–4, 7, 9, 10, 13, 14]

*Artemisiawudanica* Liou & W.Wang [8, 9, 12]

SE*Artemisiaxanthochloa* Krasch. [3–16]

*Artemisiaxerophytica* Krasch. [6, 7, 8, 10–16]

*Askelliaflexuosa* (Ledeb.) W.A.Weber [1, 3, 5–11, 13–16]

*Askelliapygmaea* (Ledeb.) Sennikov [1, 3, 6, 7]

*Asteralpinus* L. [1–10, 13]

*Asterhispidus* Thunb. [2–6, 8–11, 13, 15]

*Asterlingii* G.J.Zhang & T.G.Gao [= *Rhinactinidialimoniifolia* Novopokr.] [7]

*Astermaackii* Regel [5]

E *Astersanczirii* Kamelin & Gubanov [5]

*Astertataricus* L.f. [2, 3, 4, 5, 9]

SE*Asterothamnusalyssoides* (Turcz.) Novopokr. [= *Asteralyssoides* Turcz.] [8, 12]

SEAsterothamnuscentraliasiaticusvar.potaninii (Novopokr.) Y.Ling & Y.L.Chen [≡ *Asterothamnuspotaninii* Novopokr.] [7, 8, 9, 11–16]

SE*Asterothamnusheteropappoides* Novopokr. [6, 7, 10, 14]

*Asterothamnusmolliusculus* Novopokr. [12, 15]

*Asterothamnuspoliifolius* Novopokr. [3, 6, 7, 10, 11, 13–15]

*Bidenscernua* L. [3, 4, 6, 7, 9–11]

*Bidensparviflora* Willd. [3, 4, 6, 8–10, 13]

*Bidensradiata* Thuill. [3, 4, 6–10]

*Bidenstripartita* L. [1–4, 7, 8–10, 14]

SE*Brachanthemumgobicum* Krasch. [12, 13, 16]

SE*Brachanthemummongolicum* Krasch. [12, 14]

E *Brachanthemummongolorum* Grubov [9]

*Cancriniadiscoidea* (Ledeb.) Poljakov [7, 10–16]

SE*Cancriniakrasnoborovii* Khanm. [10]

*Carduuscrispus* L. [2–7, 9]

*Carduusnutans* L. [1, 7]

*Centaureaadpressa* Ledeb. [6]

Centaureaglastifoliasubsp.intermedia (Boiss.) L.Martins [= *Centaureachartolepis* Greuter] [6, 7]

*Centaureapulchella* Ledeb. [= *Hyaleapulchella* (Ledeb.) K.Koch] [7, 14]

*Chondrillalejosperma* Kar. & Kir. [6, 7, 10, 14]

E *Chrysanthemumchalchingolicum* Grubov [5, 9]

*Chrysanthemummongolicum* Ling [≡ Chrysanthemumzawadzkiivar.mongolicum (Ling) Gubanov] [1, 2, 3]

*Chrysanthemumnaktongense* Nakai [9]

SE*Chrysanthemumsinuatum* Ledeb. [*Tanacetumsinuatum* Sch.Bip.] [6, 7]

*Chrysanthemumtrilobatum* (Poljakov) H.Ohashi & Yonek. [≡ *Ajaniatrilobata* Poljakov] [12, 13]

*Chrysanthemumzawadzkii* Herbich [1–5, 8, 9]

*Cicerbitaazurea* (Ledeb.) Beaverd [3, 7, 10]

*Cirsiumarvense* (L.) Scop. [≡ *Serratulaarvensis* L.] [2–4, 7, 9–11, 13–15]

*Cirsiumesculentum* C.A.Mey. [1–4, 6–11, 14]

*Cirsiumglabrifolium* O.Fedtsch. & B.Fedtsch. [7]

*Cirsiumhelenioides* (L.) Hill [= *Carduushelenioides* L.] [2]

*Cirsiumpendulum* Fisch. [2, 4, 5, 9]

*Cirsiumserratuloides* Hill [1, 2, 3]

*Cirsiumsetosum* (Willd.) M.Bieb. [≡ *Serratulasetosa* Willd.] [3, 4, 7–11, 14, 15]

*Cirsiumsieversii* (Fisch. & C.A.Mey.) Petr. [= *Cirsiumpolyacanthum* Kar. & Kir.] [7]

*Cirsiumvlassovianum* Fisch. [2, 5, 9]

*Cousiniaaffinis* Schrenk [14]

*Crepidiastrumakagii* (Kitag.) J.W.Zhang & N.Kilian [= *Youngiatenuicaulis* (Babc. & Stebbins) Czerep.] [2, 3, 6–8, 10–15]

*Crepidiastrumsonchifolium* (Bunge) Pak & Kawano [5]

*Crepidiastrumtenuifolium* (Willd.) Sennikov [≡ *Crepistenuifolia* Willd. ≡ *Youngiatenuifolia* (Willd.) Babc. & Stebbins] [1–11, 13, 14]

*Crepisbungei* Ledeb. [1–4, 6–9, 11]

*Crepischrysantha* Froel. [1, 2, 3, 6, 7, 10]

Crepiscrocea(Lam.)Babc.var.crocea [2, 3, 4, 6–13]

SECrepiscroceavar.czuensis (Serg.) Tzvelev [≡ *Crepisczuensis* Serg.] [6, 7]

E *Crepislomonosovae* Tzvelev [3, 13]

*Crepislyrata* (L.) Froel. [1, 7]

*Crepismulticaulis* Ledeb. [3, 7, 10, 13, 14]

*Crepispolytricha* Turcz. [1, 3, 4, 6, 7]

*Crepispraemorsa* (L.) Tausch [≡ *Hieraciumpraemorsum* L.] [4, 10]

*Crepissibirica* L. [2–5, 7, 8, 11]

*Crepistectorum* L. [2, 4, 7, 10, 14]

*Doronicumaltaicum* Pall. [1]

*Doronicumoblongifolium* DC. [7, 14]

*Doronicumturkestanicum* Cavill. [3, 7, 14]

*Echinopsdavuricus* Fisch. [= *Echinopslatifolius* Tausch] [1–5, 8, 9]

*Echinopsgmelinii* Turcz. [3, 7–16]

*Echinopshumilis* M.Bieb. [3, 7, 13–15]

*Echinopsintegrifolius* Kar. & Kir. [6, 7, 14]

*Echinopsnanus* Bunge [7, 14]

*Echinopsritro* L. [7, 14]

*Erigeronacris* L. [1–7, 9, 10, 13]

*Erigeronaltaicus* Popov [7, 14]

SE*Erigeronbaicalensis* Botsch. [1]

*Erigeroneriocalyx* (Ledeb.) Vierh. [1–3, 6, 7, 13]

*Erigeronkrylovii* Serg. [3, 7]

*Erigeronlonchophyllus* Hook. [1–7, 9, 10, 13]

*Erigeronoreades* Fisch. & C.A.Mey. [1, 3, 7, 13]

*Erigeronpetiolaris* Vierh. [3, 7]

*Erigeronpolitus* Fr. [1–4, 6, 7, 13]

*Erigeronpseudoeriocephalus* Popov [3]

*Filagoarvensis* L. [7, 8, 10, 14]

*Filifoliumsibiricum* (L.) Kitam. [= *Tanacetumsibiricum* L.] [1–5, 8, 9]

*Galatellaaltaica* Tzvelev [7, 14]

*Galatellaangustissima* (Tausch) Novopokr. [1]

*Galatelladahurica* DC. [= *Galatellamacrosciadia* Gand. = *Galatellasongorica* Novopokr.] [1–7, 9, 10]

*Galatellahauptii* Lindl. [7]

*Gnaphaliumuliginosum* L. [= *Gnaphaliumbaicalense* Kirp. & Kuprian.] [2–4, 7, 9, 10, 14]

*Helichrysumarenarium* Moench [7]

*Heteropappusaltaicus* Novopokrov. [≡ *Asteraltaicus* Willd.] [1–4, 6–8, 10, 12–16]

*Heteropappusbiennis* (Ledeb.) Tamamsch. [1–5, 8, 9]

SE*Heteropappusmedius* (Krylov) Tamamsch. [3, 4, 5, 8, 9]

SE*Hieraciumczadanense* Tupitz. [1, 10]

*Hieraciumkorshinskyi* Zahn [2, 4]

*Hieraciumnarymense* Schischk. & Serg. [2, 4]

*Hieraciumrobustum* Fr. [8, 9]

*Hieraciumsershukense* Üksip [7]

*Hieraciumsubramosum* Lonnr. [2, 4]

*Hieraciumumbellatum* L. [1–5, 7, 8, 9, 10]

*Hieraciumvirosum* Pall. [2, 3, 4, 5, 7, 9]

*Hololeionmaximowiczii* Kitam. [9]

*Hypochaerismaculata* L. [≡ *Trommsdorffiamaculata* (L.) Bernh.] [4, 8]

*Inulajaponica* Thunb. [2]

*Inulalinariifolia* Turcz. [5, 8, 10, 11]

*Inulasalsoloides* Ostenf. [≡ *Limbardasalsoloides* Ikonn.] [8, 11, 12, 13, 15, 16]

Ixerischinensis(Thunb.)Kitagawasubsp.chinensis s.l. [= *Ixeridiumgraminifolium* (Ledeb.) Tzvelev, = *Ixeridiumgramineum* (Fisch.) Tzvelev] [2–5, 7–9, 12, 14]

*Jacobaeaambracea* (Turcz.) B.Nord. [= *Senecioambraceus* Turcz.] [2, 3, 4, 6–10, 14]

*Jacobaeacannabifolia* (Less.) E.Wiebe [≡ *Seneciocannabifolius* Less.] [2, 3, 5, 9]

Jacobaeaerucifoliasubsp.argunensis (Turcz.) Veldkamp [≡ *Senecioargunensis* Turcz.] [5, 9]

*Jacobaeaerucifolia* (L.) G.Gaertn., B.Mey. & Scherb. subsp.erucifolia [= *Senecioerucifolius* L.] [2–4, 6, 7, 9, 10]

*Jacobaeavulgaris* Gaertn. [≡ *Seneciojacobaea* L.] [3, 4, 7–10, 14]

*Jurineachaetocarpa* (Ledeb.) Ledeb. [7, 14]

*Jurineamargalensis* Iljin [7, 14]

SE*Jurineamongolica* Maxim. [= *Jurineapotaninii* Ilijn] [10–14]

*Jurineamultiflora* B.Fedtsch. [7, 14]

*Kareliniacaspia* Less. [14, 15, 16]

*Kaschgariakomarovii* (Krasch. & Rubtzov) Poljakov [≡ *Tanacetumkomarovii* Krasch. & Rubtzov] [7, 14, 15]

*Klaseacardunculus* (Pall.) Holub [≡ *Serratulacardunculus* (Pall.) Schischk.] [2, 3, 4, 5, 7]

*Klaseacentauroides* (L.) Cass. [≡ *Serratulacentauroides* L.] [1–5, 7–13]

*Klaseamarginata* (Tausch) Kitag. [≡ *Serratulamarginata* Tausch] [1–4, 7–10, 13, 14]

*Klaseasogdiana* (Bunge) L.Martins [≡ *Serratulasogdiana* Bunge, *Serratulaalatavica* C.A.Mey.] [6]

*Lactucaserriola* L. [= Lactucasativasubsp.serriola (L.) Frietema] [7, 10, 14, 15]

*Lactucasibirica* Benth. [2–6, 8, 9, 11]

*Lactucatatarica* C.A.Mey. [3, 4, 6–16]

*Lactucaundulata* Ledeb. [7, 14]

*Leibnitziaanandria* (L.) Turcz. [2, 3, 4, 5, 9]

*Leontopodiumcampestre* Hand.-Mazz. [1–3, 6–9, 11, 13, 14]

*Leontopodiumconglobatum* Hand.-Mazz. [1– 9, 13]

*Leontopodiumleontopodioides* (Willd.) Beauverd [1–5, 8, 9, 16]

*Leontopodiumnanum* (Hook.f. & Thomson) Hand.-Mazz. [16]

*Leontopodiumochroleucum* Beauverd [1–3, 6, 7, 13]

*Leontopodiumpalibinianum* Beauverd [2, 4, 5]

*Leuzeacarthamoides* DC. [= *Rhaponticumcarthamoides* (Willd.) Iljin] [7]

*Leuzearepens* (L.) D.J.N.Hind, [≡ *Rhaponticumrepens* (L.) Hidalgo ≡ *Acroptilonrepens* (L.) DC.] [6, 7, 10–16]

*Leuzeauniflora* (L.) Holub [= *Rhaponticumuniflorum* (L.) DC.] [1–5, 8, 9]

*Ligulariaaltaica* DC. [6, 7]

*Ligulariafischerii* (Ledeb.) Turcz. [2, 3, 4, 5, 9]

*Ligulariaglauca* (L.) O.Hoffm. [7]

*Ligulariahodgsonii* Hook.f. [5, 9]

*Ligulariamongolica* DC. [5, 9]

*Ligulariaprzewalskii* Diels [9, 12]

*Ligulariasagitta* (Maxim.) Mattf. [≡ *Seneciosagitta* Maxim.] [4, 5, 9]

*Ligulariasibirica* Cass. [1, 2, 3, 4, 5, 9]

*Lipschitziadivaricata* (Turcz.) Zaika, Sukhor. & N.Kilian [≡ *Scorzoneradivaricata* Turcz.] [6–13, 15, 16]

*Matricariachamomilla* L. [= *Matricariarecutita* L.] [2]

*Neopallasiapectinata* (Pall.) Poljakov [1–4, 6–16]

SE*Olgaealeucophylla* (Turcz.) Iljin [8, 9, 11–13]

SE*Olgaealomonossowii* (Trautv.) Iljin [9]

*Omalothecasupina* (L.) DC. [= *Gnaphaliumsupinum* L.] [2, 7]

*Packeracymbalaria* (Pursh) W.A.Weber & Á.Löve [≡ *Seneciocymbalaria* Pursh] [1, 3, 7]

*Paraseneciohastatus* (L.) H.Koyama [≡ *Cacaliahastata* L.] [1, 2, 3, 4, 5, 9]

*Pentanemaasperum* (Poir.) G.V.Boiko & Korniy. [≡ *Inulaaspera* Poir.] [2, 3, 9]

*Pentanemabritannica* (L.) D.Gut.Larr. [≡ *Inulabritannica* L.] [1– 11, 13, 14]

*Pentanemasalicinum* (L.) D.Gut.Larr. [≡ *Inulasalicina* L.] [2, 3, 4, 5, 9]

*Petasitesfrigidus* (L.) Fr. [1]

*Petasitesradiatus* (J.F.Gmel.) Toman [1]

*Petasitesrubellus* (J.F.Gmel.) Toman [1, 3]

*Phalacrachenacalva* (Ledeb.) Iljin [10]

*Picrisdavurica* Fisch. [1, 3, 4, 8, 9]

*Picrishieracioides* L. [2, 3, 4, 5]

*Picrisjaponica* Thunb. [2, 3, 4, 5, 9]

*Piloselladublitzkii* (B.Fedtsch. & Nevski) Sennikov [≡ *Hieraciumdublitzkii* B.Fedtsch. & Nevski] [7]

*Pilosellaechioides* (L.) F.W.Schultz & Sch.Bip. [≡ *Hieraciumechioides* L.] [2, 4]

*Pulicariavulgaris* Gaertn. [10]

*Rhinactinidiaeremophila* (Bunge) Novopokr. [= Rhinactinidiaeremophilasubsp.grubovii Botsch.] [3, 6, 7, 10, 11, 13, 14]

*Richteriapyrethroides* Kar. & Kir. [≡ *Pyrethrumpyrethroides* (Kar. & Kir.) B.Fedtsch.] [7]

*Saussureaacuminata* Turcz. [2, 4, 5]

SE*Saussureaalaschanica* Maxim. [6, 10]

*Saussureaalata* DC. [4, 6, 10]

*Saussureaalpina* (L.) DC. [1–3, 6, 7, 13]

*Saussureaamara* (L.) DC. [1–5, 7–12, 14]

SE*Saussureaarctecapitulata* Lipsch. [1, 3]

*Saussureabaicalensis* B.L.Rob. [1, 2, 3, 7]

SE*Saussureabogedaensis* Yu J.Wang & J.Chen [14]

SE*Saussureacatharinae* Lipsch. [15]

SE*Saussureaceterachifolia* Lipsch. [3, 6, 7]

*Saussureacongesta* Turcz. [1]

*Saussureacontroversa* DC. [1, 2, 3, 5]

*Saussureacoronata* Schrenk [= *Saussureadshungarica* Iljin] [7]

*Saussureadaurica* Adams [3, 6–16]

SE*Saussureadorogostaiskii* Palib. [1, 2]

*Saussureaelata* Ledeb. [7]

*Saussureaelegans* Ledeb. [= *Saussureaamoena* Kar. & Kir.] [3, 6, 7]

SE*Saussureaelongata* DC. [1, 2, 4]

*Saussureafoliosa* Ledeb. [6, 7]

*Saussureaglacialis* Herder [1, 3, 6, 7, 13, 14]

SE*Saussureagrubovii* Lipsch. [7, 14, 15]

E *Saussureagubanovii* Kamelin [15]

*Saussureainvolucrata* (Kar. & Kit.) Sch.Bip. [1–3, 6, 7, 13, 14]

*Saussureajaponica* (Thunb.) DC. [9]

*Saussureaklementzii* Lipsch. [7]

SE*Saussureakrasnoborovii* S.V.Smirn. [1]

*Saussureakrylovii* Schischk. & Serg. [7]

*Saussurealaciniata* Ledeb. [3, 4, 6–8, 10, 11, 13–16]

*Saussurealatifolia* Ledeb. [3, 7]

*Saussurealeucophylla* Schrenk [1, 3, 6, 7, 13]

SE*Saussurealipschitzii* Filatova [7, 13]

*Saussureamongolica* (Franch.) Franch. [5]

*Saussureaneoserrata* Nakai [2, 5]

*Saussureaodontolepis* Sch.Bip. [5]

E *Saussureaodorata* E.Pjak [7]

*Saussureaorgaadayi* Khanm. & Krasnob. [3, 7]

*Saussureaparviflora* (Poir.) DC. [1–7, 9]

SE*Saussureapopovii* Lipsch. [14]

*Saussureapricei* N.D.Simpson [3, 6–8, 10, 11, 13, 14]

*Saussureapseudoalpina* N.D.Simpson [1–3, 6, 7, 13, 14]

*Saussureapseudosalsa* Lipsch. [15, 16]

*Saussureapulchella* Fisch. [5, 7, 8, 9]

SE*Saussureapurpurata* (Fisch.) Lipsch. [2, 4]

E *Saussurearamosa* Lipsch. [3, 10, 11, 15]

*Saussurearecurvata* (Maxim.) Lipsch. [2, 5]

*Saussurearuncinata* DC. [2–4, 7, 8, 10]

E *Saussureasaichanensis* Kom. [1–3, 6, 7, 13, 14]

*Saussureasalicifolia* DC. [2–9]

*Saussureasalsa* Spreng. [3, 5–11, 14, 16]

*Saussureaschanginiana* (Wydler) Fisch. [1–3, 6, 7, 13]

SE*Saussureasquarrosa* Turcz. [1]

*Saussureastubendorffii* Herder [1, 3]

*Saussureasubacaulis* (Ledeb.) Serg. [1, 3, 6, 7, 13]

SE*Saussureasukaczevii* Lipsch. [1, 2, 3]

*Saussureaussuriensis* Maxim. [5]

*Scorzoneraalbicaulis* Bunge [1, 2, 4, 5, 9]

*Scorzoneracurvata* (Popl.) Lipsch. [3, 7, 8, 9, 13]

E *Scorzoneragrubovii* Lipsch. [7, 14]

*Scorzoneraparviflora* Jacq. [14]

*Scorzoneraradiata* Fisch. [1–10, 13, 14]

*Scorzonerasinensis* (Lipsch. & Krasch.) Nakai [9]

*Seneciodubitabilis* C.Jeffrey & Y.L.Chen [≡ *Seneciodubius* Ledeb. nom. illegit. non Beck] [2, 3, 7, 8, 10–15]

E *Seneciokenteicus* Grubov [2]

*Senecionemorensis* L. [1, 2, 3]

*Seneciosubdentatus* Ledeb. [7, 10, 14, 15]

*Seneciovulgaris* L. [1–4, 7, 8, 10]

*Serratulacoronata* L. [5, 9]

*Serratulakirghisorum* Iljin [7]

*Solidagodahurica* (Kitag.) Kitag. [1–5, 7, 9]

*Solidagovirgaurea* L. [7]

*Sonchelladentata* (Ledeb.) Sennikov [≡ *Sonchusdentatus* Ledeb.] [10, 14, 15]

*Sonchellastenoma* (Turcz.) Sennikov [≡ *Crepisstenoma* Turcz.] [8–15]

*Sonchusarvensis* L. [2–5, 7–11, 13, 14]

*Sonchusbrachyotus* DC. [8–10, 13, 14]

*Sonchusuliginosus* M.Bieb. [4, 5, 8, 9, 10]

*Stilpnolepisintricata* (Franch.) C.Shih [3, 4, 7, 9–15]

*Symphyotrichumciliatum* (Ledeb.) G.L.Nesom [3, 4, 9, 10]

*Synurusdeltoides* (Aiton) Nakai [4, 5]

*Takhtajanianthaaustriaca* (Willd.) Zaika, Sukhor. & N.Kilian [≡ *Scorzoneraaustriaca* Willd.] [2–10, 12–14]

*Takhtajanianthacapito* (Maxim.) Zaika, Sukhor. & N.Kilian [≡ *Scorzoneracapito* Maxim.] [8, 11–16]

*Takhtajanianthaikonnikovii* (Krasch. & Lipsch.) Zaika, Sukhor. & N.Kilian [≡ *Scorzoneraikonnikovii* Lipsch. & Krasch.] [3, 6–15]

*Takhtajanianthamongolica* (Maxim.) Zaika, Sukhor. & N.Kilian [≡ *Scorzoneramongolica* Maxim.] [10–16]

*Takhtajanianthapseudodivaricata* (Lipsch.) Zaika, Sukhor. & N.Kilian [≡ *Scorzonerapseudodivaricata* Lipsch] [3, 6, 7, 9, 10–16]

*Takhtajanianthapusilla* (Pall.) Nazarova [≡ *Scorzonerapusilla* Pall.] [8, 14]

*Takhtajanianthasubacaulis* (Regel) Zaika, Sukhor. & N.Kilian [= *Scorzonerasubacaulis* (Regel) Lipsch.] [6]

*Tanacetumalatavicum* Herder [≡ *Pyrethrumalatavicum* O.Fedtsch. & B.Fedtsch.] [7]

E *Tanacetumchangaicum* (Krasch.) K.Bremer & Humphries [≡ *Pyrethrumchangaicum* Krasch.] [3, 4, 7, 10]

*Tanacetumcrassipes* (Stschegl.) Tzvelev [7]

*Tanacetumkrylovianum* (Krasch.) K.Bremer & Humphries [≡ *Pyrethrumkrylovianum* Krasch.] [7]

*Tanacetumlanuginosum* Sch. [≡ *Pyrethrumlanuginosum* (Sch.Bip. & Herder) Tzvelev] [1, 6, 7, 13]

SE*Tanacetumpulchellum* Sch. [≡ *Pyrethrumpulchellum* Turcz.] [7]

*Tanacetumpulchrum* Sch. [= *Pyrethrumpulchrum* Ledeb.] [3, 6, 7, 13]

*Tanacetumtanacetoides* (DC.) Tzvelev [2, 3, 6, 7]

*Tanacetumvulgare* L. [= *Tanacetumboreale* Fisch. & DC.] [1–7, 9]

*Taraxacumarmeriifolium* Soest [3–5, 7, 9–14, 16]

*Taraxacumasiaticum* Dahlst. [4, 7, 8, 13]

*Taraxacumatrans* Schischk. [7, 13]

*Taraxacumbessarabicum* (Hornem.) Hand.-Mazz. [2–4, 7–10]

*Taraxacumbicorne* Dahlst. [1, 2, 3, 7, 9–12]

SE*Taraxacumbornuurense* R.Doll [3, 4, 6, 7]

*Taraxacumbrevirostre* Hand.-Mazz. [3, 7, 13]

*Taraxacumceratophorum* (Ledeb.) DC. [= *Taraxacumaltaicum* Schischk.] [1–7, 9, 13, 14]

*Taraxacumcollinum* DC. [3, 4, 6–10, 14]

*Taraxacumdealbatum* Hand.-Mazz. [1–4, 6–15]

*Taraxacumdissectum* Ledeb. [1–4, 6–10, 12, 13]

*Taraxacumeriopodum* DC. [6, 7, 13, 14]

*Taraxacumerythrospermum* Andrz. [3]

*Taraxacumglabrum* DC. [1–3, 6, 7, 14]

*Taraxacumglaucanthum* Nakai [3, 4, 8]

*Taraxacumgoloskokovii* Schischk. [6, 7, 10, 13]

E *Taraxacuminimitabile* Kirschner & Štěpánek [13]

E *Taraxacumjunatovii* Tzvelev [3, 7, 13, 14]

*Taraxacumkok-saghyz* Rodin [3, 7, 13]

SE*Taraxacumkrasnoborovii* Krasnikov [7]

SE*Taraxacumkrylovii* Krasnikov & Khanm. [7]

*Taraxacumleucanthum* Ledeb. [1–4, 6–8, 10–15]

*Taraxacumlinczevskyi* Schischk. [7]

SE*Taraxacumlongicorne* Dahlst. [1, 2, 5–10]

*Taraxacumluridum* G.E.Haglund [6, 7]

*Taraxacumlyratum* (Ledeb.) DC. [1, 3, 6, 7]

*Taraxacummacilentum* Dahlst. [1, 3, 6, 7]

*Taraxacummicrospermum* Schischk. [= *Taraxacumcompactum* Schischk.] [1, 2]

*Taraxacumminutilobum* Popov [7]

*Taraxacummongolicum* Hand.-Mazz. [1–4, 6, 7, 10, 11, 13]

*Taraxacummongoliforme* R.Doll [1, 2, 4, 7–11, 13, 15]

*Taraxacummonochlamydeum* Hand.-Mazz. [3, 4, 7, 12–15]

*Taraxacummujense* Petrochenko [1, 2]

*Taraxacummultisectum* Kitag. [9]

*Taraxacumofficinale* F.H.Wigg. [1, 2, 3, 4]

*Taraxacumparvulum* DC. [14]

*Taraxacumpawlodarskum* R.Doll [= *Taraxacumustamenum* R.Doll] [7]

*Taraxacumpingue* Schischk. [1, 3, 6, 7]

*Taraxacumpseudoatratum* Orazova [6]

SE*Taraxacumpseudonivale* Malyschev [1]

*Taraxacumpuberulum* G.E.Haglund [14]

SE*Taraxacumsangilense* Krasnob. & Khanm. [1, 2, 3, 4, 6, 7]

*Taraxacumscariosum* (Tausch) Kirschner & Štěpánek [= *Taraxacumstenolobum* Stschegl., *Taraxacumcommixtiforme* Soest] [4, 8–10, 13, 14]

E *Taraxacumselengensis* Tzvelev [3]

*Taraxacumsinicum* Kitag. [= *Taraxacumborealisinense* Kitam.] [3–16]

SE*Taraxacumsmirnovii* M.S.Ivanova [7]

SE*Taraxacumsongoricum* Schischk. [6, 7, 13]

*Taraxacumstanjukoviczii* Schischk. [7, 13]

E *Taraxacumsubmacilentum* Tzvelev [7]

*Taraxacumsumneviczii* Schischk. [1, 7, 13]

*Taraxacumtibetanum* Hand.-Mazz. [13, 14]

*Taraxacumturgaicum* Schischk. [7, 13, 14]

SE*Taraxacumtuvense* Krasnob. & Krasnikov [1]

*Tephroserisflammea* (DC.) Holub [≡ *Senecioflammeus* DC.] [5]

Tephroserisintegrifoliasubsp.atropurpurea (Ledeb.) B.Nord. [1]

Tephroserisintegrifolia(L.)Holubsubsp.integrifolia [= *Seneciocampestris* (Retz.) DC.] [1–4, 6–9, 13]

*Tephroseriskirilowii* (DC.) Holub [5]

*Tephroserispalustris* (L.) Rchb. [≡ *Seneciopalustris* (L.) Hook.] [1, 2, 4–10]

SE*Tephroserisporphyrantha* (Schischk.) Holub [= *Senecioporphyranthus* Schischk.] [1, 7]

*Tephroserispraticola* (Sisk. & Serg.) Holub [= *Senecioasiatica* Schischk. & Serg.] [1, 2, 3, 7]

*Tephroserispricei* (N.D.Simpson) Holub [≡ *Seneciopricei* N.D.Simpson] [1, 3, 6, 7, 13, 14]

SE*Tephroserissukaczevii* (Schischk.) Holub [≡ *Seneciosukaczevii* Schischk.] [2, 4, 9]

*Tephroseristurczaninovii* (DC.) Holub [≡*Seneciosumneviczii* Schischk. & Serg.] [1, 2, 3, 6, 7]

*Tephroserisvereszczaginii* (Schischk. & Serg.) Holub [≡ *Senecioveresczaginii* Schischk. & Serg.] [7]

*Tibetiodesflaccida* (Bunge) G.L.Nesom [≡ *Erigeronflaccidus* (Bunge) Botsch.] [1, 2, 3, 4, 6, 7]

*Tragopogonkasahstanicus* S.A.Nikitin [7]

*Tragopogonorientalis* L. [6, 7]

*Tragopogonruber* S.G.Gmel. [7, 14]

*Tragopogonsongoricus* S.A.Nikitin [6, 7, 13, 14]

SE*Tragopogontrachycarpus* S.A.Nikitin [2–5, 7, 8, 13]

*Tripleurospermumambiguum* (Ledeb.) Franch. & Sav. [≡ *Matricariaambigua* (Ledeb.) Krylov] [6, 7]

*Tripoliumpannonicum* (Jacq.) Dobrocz. [≡ *Tripoliumpannonicum* Jacq.] [4, 9–13]

*Trommsdorffiaciliata* (Thunb.) Soják [≡ *Hypochaerisciliata* (Thunb.) Makino] [5]

SE*Tugarinoviamongolica* Iljin [11, 12, 13, 16]

*Turczaninoviafastigiata* (Fisch.) DC. [≡*Asterfastigiatus* Fisch.] [2, 4]

*Vickifunkiasongarica* (Fisch.) C.Ren [≡ *Ligulariasongarica* (Fisch.) Y.Ling] [14]

*Vickifunkiathomsonii* (C.B.Clarke) C.Ren [≡ *Ligulariathomsonii* (C.B.Clarke) Pojark.] [14]

*Vickifunkiathyrsoidea* (Ledeb.) C.Ren [≡ *Ligulariathyrsoidea* (Ledeb.) DC.] [6, 7, 14]

**29. Balsaminaceae** A.Rich. (1 genus and 2 species)

*Impatiensnoli-tangere* L. [1, 2, 3, 4, 5, 9]

*Impatiensparviflora* DC. [7]

**30. Berberidaceae** Juss. (1 genus and 2 species)

*Berberisamurensis* Rupr. [5]

*Berberissibirica* Pall. [1–4, 6, 7, 9, 10, 13]

**31. Betulaceae** Gray (2 genera and 9 taxa)

Alnusalnobetulasubsp.fruticosa (Rupr.) Raus [1, 2, 4, 9]

*Betulafruticosa* Pall. [1, 2, 3, 4, 5, 6]

Betulamandshuricasubsp.tauschii (Regel) Kamelin [4, 5]

*Betulamicrophylla* Bunge [1–4, 6–8, 10, 13, 14]

Betulananasubsp.exilis (Sukachev) Hultén [2, 3]

Betulananasubsp.rotundifolia (Spach) Malyschev [1, 2, 3, 6, 7]

*Betulaovalifolia* Rupr. [1–5, 7, 8, 13]

Betulapendulasubsp.mandshurica (Regel) Ashburner & McAll. [1–5, 8, 13]

BetulapendulaRothsubsp.pendula [2, 3, 4]

**32. Biebersteiniaceae** Schnizl. (1 genus and 1 species)

*Biebersteiniaodora* Stephan [6, 7]

**33. Bignoniaceae** Juss. (1 genus and 1 species)

SE*Incarvilleapotaninii* Batalin [13, 15, 16]

**34. Boraginaceae** Juss. (24 genera and 78 taxa)

Note: Since Urgamal et al. (2014), several genera and species have been critically revised and updated by Ovczinnikova (2019a). Additionally, six new species of *Craniospermum* Lehm. have been described from Mongolia by Ovczinnikova and Korolyuk (2016) and Ovczinnikova (2019b, 2020). We follow the treatment of Ovczinnikova (2019a). Furthermore, *Arnebiatibetica* previously known as a synonym of *A.guttata*, differs from *A.guttata* based on floral morphology and plastid genome characteristics discovered by Park et al. (2020).

*Amblynotusrupestris* (Pall.) Popov [≡ *Eritrichiumrupestre* (Georgi) Bunge] [1–9, 13]

*Anchusaarvensis* (L.) M.Bieb. [7, 10, 11, 14]

SE*Anoplocaryumcompressum* Ledeb. [≡ *Echinospermumcompressum* (Ledeb.) Turcz.] [1, 2, 3, 6, 8]

E *Anoplocaryumtenellum* A.L.Ebel & Rudaya [≡ *Microulatenella* (A.L.Ebel & Rudaya)] [7]

SE*Anoplocaryumturczaninovii* Krasnob. [1, 3, 6, 7, 8, 10, 14]

*Arnebiadecumbens* Coss. & Kralik [6, 7, 8, 13, 14]

*Arnebiafimbriata* Maxim. [11–13, 15, 16]

*Arnebiaguttata* Bunge [3, 7, 10–16]

*Arnebiatibetana* Kurz [7]

*Asperugoprocumbens* L. [3, 6, 7, 10, 14]

E *Asperulagobicola* Grubov [= *Asperulasaxicola* Grubov] [13, 16]

SE*Craniospermumcanescens* DC. [3, 7, 13, 14]

E *Craniospermumdesertorum* Ovczinnikova & A.Korolyuk [7]

E *Craniospermumgubanovii* Ovczinnikova [14]

E *Craniospermumkamelinii* Ovczinnikova [7]

SE*Craniospermummongolicum* I.M.Johnst. [7, 11–14]

E *Craniospermummontanostepposum* Ovczinnikova [7]

E *Craniospermumpseudotuvinicum* Ovczinnikova & A.Korolyuk [10]

SE*Craniospermumtuvinicum* Ovczinnikova [6, 7]

E *Craniospermumvolkovae* Ovczinnikova [10]

*Cynoglossumdivaricatum* Steph. [3, 4, 8, 9, 13, 14]

SE*Eritrichiumalpinum* Ovczinnikova [6]

*Eritrichiumpauciflorum* DC. [1–8, 13]

*Eritrichiumpectinatum* DC. [3]

SE*Eritrichiumpulviniforme* Popov [3, 10, 13]

SE*Eritrichiumsajanense* (Malysch.) Sipliv. [1]

*Eritrichiumthymifolium* (DC.) Y.S.Lian & J.Q.Wang [3, 4, 6–15]

*Eritrichiumtianschanicum* Iljin [6]

*Eritrichiumvillosum* (Ledeb.) Bunge [≡ *Myosotisvillosa* Ledeb.] [2, 3, 4, 6, 7, 14]

*Hackeliadeflexa* (Wahlenb.) Opiz [≡ *Myosotisdeflexa* Wahlenb.] [2–5, 7, 9, 10, 13]

*Heliotropiumellipticum* Ledeb. [6, 7, 15]

*Lappulabalchaschensis* Popov [7, 13, 14, 15]

*Lappulabrachycentroides* Popov [3]

*Lappulaconsanguinea* Gürke [2–4, 6, 7, 10, 11, 13, 14]

*Lappulacoronifera* Popov [3]

*Lappuladuplicicarpa* Pavlov [7, 12, 14]

SE*Lappulagranulata* (Krylov) Popov [3, 7, 9, 10, 12]

*Lappulaheteracantha* (Ledeb.) Gürke [7]

*Lappulaintermedia* (Ledeb.) Popov [3, 4, 6, 7, 9, 14, 15]

*Lappulakrylovii* Ovczinnikova, Pjak & A.L.Ebel [7]

*Lappulamacrantha* (Ledeb.) Gürke [7, 14]

*Lappulamicrocarpa* Gürke [7, 10]

*Lappulamyosotis* Wolf [2–5, 8, 9, 13]

*Lappulapatula* Asch. [3, 15]

*Lappularedowskii* (Hornem.) Greene [1–4, 8, 9, 11–13]

*Lappulasemiglabra* (Ledeb.) Gürke [7, 11, 14, 15]

*Lappulastricta* (Ledeb.) Gürke [3, 7–12, 14, 15]

*Lappulatadshikorum* Popov [7]

*Lappulatenuis* Gürke [14, 15]

*Lappulatianschanica* Popov & Zakirov [7]

*Lappulatuvinica* Ovczinnikova [6]

*Lindelofiastylosa* (Kar. & Kir.) Brand [≡ *Cynoglossumstylosum* Kar. & Kir.] [7, 10, 11, 14]

*Mertensiadavurica* (Sims) G.Don [= *Mertensiaochroleuca* Ikonn.-Gal.] [1, 2, 3, 4]

*Mertensiapallasii* G.Don [7]

*Mertensiastylosa* DC. [1, 2, 3]

*Mertensiatarbagataica* B.Fedtsch. [7]

SEMicroulatibeticavar.pratensis (Maxim.) W.T.Wang [≡ *Tretocaryapratensis* Maxim.] [3, 7]

*Myosotisalpestris* F.W.Schmidt [1–4, 6, 7, 9, 14]

*Myosotisaustrosibirica* O.D.Nikif. [7, 13]

*Myosotisbaltica* Sam. [3, 5]

*Myosotiscaespitosa* Schultz [2–5, 9, 10, 14]

*Myosotiskrylovii* Serg. [1–4, 6, 7, 13]

*Myosotisscorpioides* L. [2]

*Myosotisstricta* Link [7]

*Noneacaspica* G.Don [7, 10, 11, 14, 15]

*Noneapulla* DC. [2, 4, 8, 9, 14]

*Nonearossica* Steven [3]

*Onosmafuyunensis* Y.He & Q.R.Liu [7]

*Onosmagmelinii* Ledeb. [7, 14]

OnosmasetosaLedeb.subsp.setosa [7]

Onosmasetosasubsp.transrhymnensis (Klokov) Kamelin [3, 7, 10]

*Pseudolappulaoccultata* (Popov) Q.R.Liu & D.H.Liu [≡ *Lappulaoccultata* Popov] [14]

*Pulmonariadacica* (Simonk.) Simonk. [= *Pulmonariamollissima* A.Kern.] [2, 4]

*Rinderatetraspis* Pall. [14]

*Rocheliabungei* Trautv. [3, 6, 14]

*Rochelialeiocarpa* Ledeb. [6, 14]

*Stenosoleniumsaxatile* (Pall.) Turcz. [≡ *Anchusasaxatilis* Pall.] [3, 4, 10]

*Tournefortiasibirica* L. [= *Messerschmidiasibirica* (L.) L.] [5, 8–13, 16]

**35. Brassicaceae** Burnett (51 genera and 138 taxa)

Note: The updated checklist and taxonomic notes of Brassicaceae was recently revised by German (2015). In this study, we followed German (2015) where the names of several species and genera changed compared to Urgamal et al (2014). Since 2015, several new records of this family have been found in the flora of Mongolia (Dorofeyev 2019; Dorofeyev and Ekhmaa 2020). For example, *Lepidiumgobicum* V.I.Dorof. was newly described from Mongolia and China by Dorofeyev (2019); however, this species should be referred to *Lepidiumapetalum* Willd. (German 2020).

*Alyssumdesertorum* Stapf [3, 6, 7, 8, 10]

*Alyssumlenense* Adams [1–5, 7, 8, 9]

*Aphragmusinvolucratus* O.E.Schulz [7, 13]

*Arabidopsisthaliana* (L.) Heynh. [6, 7]

*Arabisborealis* Andrz. [2, 3, 4, 5, 9]

*Barbareaorthoceras* Ledeb. [2, 3, 4, 5, 9]

*Barbareavulgaris* W.T.Aiton [2, 3, 4, 5, 7]

*Brayahumilis* (C.A.Mey.) B.L.Rob. [= *Neotorulariagrubovii* (Botsch.) Botsch., *Neotorulariamongolica* Botsch. & Gubanov] [1, 3, 4, 6, 7, 8]

*Brayarosea* Bunge [1, 3, 6, 7]

*Brayasiliquosa* Bunge [1]

*Camelinamicrocarpa* Andrz. [4, 6, 14]

*Capsellabursa-pastoris* (L.) Medik. [2–4, 6, 7, 10, 14]

*Capsellaorientalis* Klokov [≡ Capsellabursa-pastorissubsp.orientalis (Klokov) Tzvelev] [7, 10]

*Cardaminebellidifolia* L. [1, 2, 3, 7]

*Cardamineimpatiens* L. [6]

*Cardamineleucantha* (Tausch) O.E.Schulz [5]

*Cardaminemacrophylla* Willd. [1, 2, 6, 7]

*Cardamineparviflora* L. [2, 3]

*Cardaminepratensis* L. [1–5, 7, 9]

*Cardamineprorepens* Fisch. [5]

*Cardaminetrifida* (Lam.) B.M.G.Jones [5]

*Catolobuspendulus* (L.) Al-Shehbaz [= *Arabispendula* L.] [1–6, 8, 9, 10, 12, 13]

*Chorisporabungeana* Fisch. & C.A.Mey. [7]

*Chorisporasibirica* (L.) DC. [6, 7, 13, 14]

*Chorisporatenella* (Pall.) DC. [7, 14]

*Clausiaaprica* Trotzky [1–4, 6, 7, 9]

*Clausiatrichosepala* (Turcz.) F.Dvořák [4]

*Crucihimalayamollissima* (C.A.Mey.) Al-Shehbaz [≡ *Sisymbriummollissimum* C.A.Mey.] [6, 7, 9, 13, 14]

SE*Crucihimalayarupicola* (Krylov) A.L.Ebel & D.A.German [≡ *Arabisrupicola* Krylov] [6, 7, 10, 11, 13, 14]

*Dendroarabisfruticulosa* (C.A.Mey.) D.A.German & Al-Shehbaz [≡ *Arabisfruticulosa* C.A.Mey.] [1, 7]

*Descurainiasophia* (L.) Webb [1–10, 12–14]

SE*Dontostemoncrassifolius* (Bunge) Maxim. [7, 10–16]

*Dontostemondentatus* Ledeb. [5]

SE*Dontostemonelegans* Maxim. [6, 7, 10, 11, 13–16]

E *Dontostemongubanovii* (D.A.German) D.A.German [≡ Dontostemonsenilissubsp.gubanovii D.A.German] [6, 7, 10]

*Dontostemonintegrifolius* (L.) Ledeb. [1–13, 16]

*Dontostemonmicranthus* C.A.Mey. [1–5, 8, 9, 13]

SE*Dontostemonperennis* C.A.Mey. [3, 5–8, 10–13, 15]

*Dontostemonpinnatifidus* (Willd.) Al-Shehbaz & H.Ohba [≡ *Cheiranthuspinnatifidus* Willd.] [1, 3, 4, 8, 13]

SE*Dontostemonsenilis* Maxim. [6, 7, 8, 10–16]

*Drabaalpina* L. [6]

*Drabaaltaica* (C.A.Mey.) Bunge [6, 7, 13]

SE*Drababaicalensis* Tolm. [3, 6, 7]

*Drabaeriopoda* Turcz. [1, 2, 3, 6]

*Drabafladnizensis* Wulfen [1, 2, 3, 6, 7, 13]

*Drabahirta* L. [1, 2, 3, 6, 7, 13]

*Drabakusnetzovii* (Turcz.) Hayek [1, 3, 6, 7, 13]

*Drabalanceolata* Royle [1–4, 6, 7, 13]

*Drabamongolica* Turcz. [1, 3]

*Drabanemorosa* L. [1–10, 13]

*Drabaochroleuca* Bunge [1, 3, 6, 7, 13]

*Drabaoreades* Schrenk [1, 3, 6, 7, 13]

SE*Drabapygmaea* Turcz. [1, 3, 6]

*Drabasibirica* (Pall.) Thell. [3, 7]

*Drabastenocarpa* Hook.f. & Thomson [7]

*Drabasubamplexicaulis* C.A.Mey. [1–3, 6, 7, 13, 14]

*Drabaturczaninowii* Pohle [1, 6, 7, 13]

*Erysimumandrzejowskianum* Bess. [7]

Erysimumcheiranthoidessubsp.altum Ahti [without indication of regions]

ErysimumcheiranthoidesL.subsp.cheiranthoides [1–5, 7–11, 13, 14]

Erysimumcheiranthoidessubsp.transiliense (Popov) D.A.German [≡ *Erysimumtransiliense* Popov] [7]

Erysimumflavum(Georgi)Bobrovsubsp.flavum [1–5, 8, 9, 12]

Erysimumflavumsubsp.altaicum (C.A.Mey.) Polozhij [3, 6, 7, 10]

SE*Erysimumkotuchovii* D.A.German [7]

*Erysimumledebourii* D.A.German [7]

*Erysimummarschallianum* Andrz. [2–4, 6, 7, 10, 13, 14]

SE*Erysimummongolicum* D.A.German [7, 14]

*Erysimumsisymbrioides* C.A.Mey. [6, 7, 15]

Eutremaedwardsiisubsp.compactum (O.E.Schulz) A.L.Ebel [≡ *Eutremacompactum* O.E.Schulz] [7]

EutremaedwardsiiR.Br.subsp.edwardsii [1–3, 6, 7, 13]

*Eutremasalsugineum* (Pall.) Al-Shehbaz & Warwick [≡ *Sisymbriumsalsugineum* Pall.] [3, 4, 6–10]

E *Galitzkyamacrocarpa* (Ikonn.-Gal.)Botsch. [≡ *Berteroamacrocarpa* Ikonn.-Gal.] [13, 15]

SE*Galitzkyapotaninii* (Maxim.)Botsch. [7, 14, 15]

SE*Goldbachiaikonnikovii* Vassilcz. [6, 7, 8, 10, 11, 13, 14]

*Goldbachiapendula*Botsch. [7, 14]

*Hesperissibirica* L. [1, 2, 3, 4, 7]

*Hornungiaprocumbens* Hayek [3, 6, 7, 10, 11, 14]

*Iljinskaeaplanisiliqua* (Fisch. & C.A.Mey.) Al-Shehbaz [≡ *Conringiaplanisiliqua* Fisch. & C.A.Mey.] [6, 14]

*Isatiscostata* C.A.Mey. [2–4, 6–9, 11–14]

*Isatisgymnocarpa* (Fisch.) Al-Shehbaz, Moazzeni & Mumm. [≡ *Tauscheriagymnocarpa* Fisch.] [14]

*Isatismulticaulis* (Kar. & Kir.) Jafri [14]

*Isatisoblongata* DC. [1, 3, 4, 6–9, 13]

*Leiocarpaeacochlearioides* (Murray) D.A.German & Al-Shehbaz [≡ *Buniascochlearioides* Murray] [1]

*Leiosporaexscapa* (C.A.Mey.) F.Dvořák [≡ *Parryaexscapa* C.A.Mey.] [1, 6, 7]

*Lepidiumaffine* Ledeb. [≡ Lepidiumlatifoliumsubsp.affine (Ledeb.) Kitag.] [4, 9, 14]

*Lepidiumamplexicaule* Willd. [3, 7–11, 14, 15]

*Lepidiumapetalum* Willd. [1–5, 7–15]

*Lepidiumappelianum* Al-Shehbaz [7, 10, 11, 14–16.]

*Lepidiumcartilagineum* Thell. [5–8, 10, 12, 14]

*Lepidiumcordatum* Willd. [6–11, 13–16]

*Lepidiumlacerum* C.A.Mey. [= *Lepidiumsongaricum* Schrenk] [7, 14]

*Lepidiumobtusum* Basiner [6, 7, 10, 14, 15]

*Litwinowiatenuissima* (Pall.) Woronow [14]

*Macropodiumnivale* R.Br. [1, 7]

*Matthiolasuperba* Conti [14]

*Megacarpaeamegalocarpa* Schischk. [14]

*Meniocuslinifolius* (Willd.) DC. [≡ *Alyssumlinifolium* Willd.] [3, 5, 7]

SE*Microstigmabrachycarpum* Botsch. [6, 7, 15, 16]

SE*Microstigmadeflexum* (Bunge) Juz. [3, 6, 7, 12, 13, 15, 16]

*Neotorulariabrevipes* (Kar. & Kir.) Hedge & J.Léonard [≡ *Sisymbriumbrevipes* F.Muell.] [7, 14]

*Noccaeaferganensis* (N.Busch) Czerep. [≡ *Thlaspiferganense* N.Busch] [7]

*Noccaeathlaspidioides* (Pall.) F.K.Mey. [≡ *Lepidiumthlaspidioides* Pall. = *Thlaspicochleariforme* DC.] [1–9, 13]

*Odontarrhenaobovata* C.A.Mey. [≡ *Alyssumobovatum* (C.A.Mey.) Turcz.] [1–10]

*Olimarabidopsispumila* (Stephan) Al-Shehbaz [≡ *Sisymbriumpumilum* Stephan] [14]

SE*Pachyneurumgrandiflorum* Bunge [1, 3, 6, 7, 13]

*Pugioniumdolabratum* Maxim. [11, 12, 13, 16]

SE*Pugioniumpterocarpum* Kom. [10]

*Rhammatophyllumerysimoides* (Kar. & Kir.) Al-Shehbaz & O.Appel [≡ *Arabiserysimoides* Kar. & Kir.] [7, 14]

*Rorippabarbareifolia* (DC.) Kitag. [2]

*Rorippadogadovae* Tzvelev [3, 11]

*Rorippapalustris* Besser [1–11, 13, 14]

*Sisymbriumbrassiciforme* C.A.Mey. [7, 9, 14, 15]

*Sisymbriumheteromallum* C.A.Mey. [2–4, 6–8, 10–14]

*Sisymbriumloeselii* L. [3, 4, 14]

*Sisymbriumpolymorphum* (Murr.) Roth [3, 4, 6–10, 14]

*Sisymbriumsubspinescens* Bunge [14]

*Smelowskiaalba* (Pall.) B.Fedtsch. [1, 3, 4, 6, 7, 10, 13, 14]

SE*Smelowskiaaltaica* (Pobed.) Botsch. [6, 7]

*Smelowskiabifurcata* (Ledeb.) Botsch. [1, 3]

*Smelowskiacalycina* (Stephan) C.A.Mey. [= *Lepidiumcalycinum* Steph.] [1, 3, 6, 7, 13, 14]

SESmelowskiacalycinasubsp.pectinata (Bunge) D.A.German [= *Hutchinsiapectinata* Bunge] [3, 7, 13, 14]

E *Smelowskiamongolica* Kom. [3]

SE*Sterigmostemumviolaceum* (Botsch.) H.L.Yang [≡ *Oreolomaviolaceum* Botsch., = *Sterigmostemumregeliorum* Kamelin & D.German] [7, 14]

*Steveniaalyssoides* Adams & Fisch. [1, 3]

SESteveniaalyssoidessubsp.zinaidae (Malyschev) Kamelin [≡ *Steveniazinaidae* Malyschev] [1, 3]

*Steveniacanescens* (DC.) D.A.German [≡ *Alyssumcanescens* DC. ≡ *Ptilotrichumcanescens* (DC.) C.A.Mey.] [1–4, 6–9, 11–16]

SESteveniacheiranthoidesDC.subsp.cheiranthoides [4, 5, 6, 7, 9]

Steveniacheiranthoidessubsp.incarnata (Kamelin) D.A.German [1–4, 6–8, 10]

SE*Steveniadahurica* (Peschkova) D.A.German & Al-Shehbaz [≡ *Alyssumdahuricum* (Peschkova) Al-Shehbaz, *Ptilotrichumdahuricum* Peschkova] [4, 5, 8, 9]

SE*Steveniasergievskajae* (Krasnob.) Kamelin & Gubanov [≡ *Alyssumsergievskajae* Krasnob.] [3]

SE*Steveniatenuifolia* (Stephan) D.A.German [≡ *Alyssumtenuifolium* Steph.] [2–10, 12–15]

*Strigosellaafricana* (L.) Botsch. [10, 11]

*Strigosellabrevipes* (Bunge) Botsch. [14]

*Subulariaaquatica* L. [3, 6]

*Tetracmequadricornis* (Steph.) Bunge [7, 14]

*Thlaspiarvense* L. [1–4, 6, 7, 13, 14]

*Thlaspiceratocarpum* (Pall.) Murray [≡ *Carpocerasceratocarpum* (Pall.) N. Busch] [6, 10, 14]

*Turritisglabra* L. [7]

**36. Butomaceae** Mirb. (1 genus and 2 species)

*Butomusjunceus* Turcz. [1, 8, 9, 10, 14]

*Butomusumbellatus* L. [1–5, 8, 9, 14]

**37. Campanulaceae** Juss. (4 genera and 18 taxa)

E *Adenophorachangaica* Gubanov & Kamelin [3]

*Adenophoragmelinii* Fisch. [4, 5, 9]

*Adenophoralamarkii* Fisch. [≡ *Campanulalamarckii* D.Dietr.] [2, 3, 4, 6]

*Adenophoraliliifolia* (L.) A.DC. [≡ *Campanulaliliifolia* L.] [2, 6, 8]

*Adenophorapereskiifolia* (Fisch.) G.Don [4, 5, 9]

*Adenophorastenanthina* (Ledeb.) Kitagawa [= *Adenophoracrispata* Turcz.] [1–5, 8, 9, 13]

*Adenophoratricuspidata* A.DC. [2, 4, 5, 9]

*Adenophoratriphylla* (Thunb.) A.DC. [≡ *Campanulatriphylla* Thunb.] [2, 4, 9]

*Campanulacervicaria* L. [2]

*Campanuladasyantha* M.Bieb. [1, 2]

*Campanulaglomerata* L. [1–7, 9]

*Campanulapunctata* Lam. [9]

*Campanularotundifolia* L. [6]

Campanulasteveniisubsp.altaica (Ledeb.) Fed. [≡ *Campanulaaltaica* Ledeb.] [7]

Campanulasteveniisubsp.turczaninovii (Fed.) Victorov [≡ *Campanulaturczaninovii* Fed.] [1, 2, 3, 6, 13]

Campanulasteveniisubsp.wolgensis (P.A.Smirn.) Fed. [≡ *Campanulawolgensis* P.A.Smirn.] [7]

*Codonopsisclematidea* C.B.Clarke [7]

*Platycodongrandiflorus* A.DC. [5]

**38. Caprifoliaceae** Juss. (5 genera and 24 taxa)

*Linnaeaborealis* L. [1, 7, 13, 14]

Loniceracaeruleasubsp.altaica (Pall.) Gladkova [≡ *Loniceraaltaica* Pall.] [1–4, 6, 7, 13, 14]

LoniceracaeruleaL.subsp.caerulea [6]

Loniceracaeruleavar.venulosa (Maxim.) Vorosch. [≡ *Loniceravenulosa* Maxim.] [5]

*Lonicerachrysantha* Turcz. [5]

*Lonicerahispida* Pall. [3, 6, 7, 13, 14]

*Loniceramicrophylla* Willd. [≡ *Caprifoliummicrophyllum* (Willd.) Kuntze] [3, 6, 7, 9, 10, 13, 14, 16]

*Loniceratatarica* L. [4]

*Patriniaheterophylla* Bunge [9]

*Patriniaintermedia* Roem. & Schult. [3, 6, 7, 14]

*Patriniarupestris* (Pall.) Dufr. [≡ *Valerianarupestris* Pall.] [1–5, 8, 9]

*Patriniascabiosifolia* Fisch. [4, 5]

*Patriniasibirica* (L.) Juss. [1–7]

*Scabiosacomosa* Fisch. [1–5, 8, 9]

*Scabiosaochroleuca* L. [3, 10]

*Valerianaaltaica* Sumnev. [1, 2]

*Valerianaalternifolia* Ledeb. [= *Valerianadahurica* Sumnev.] [1–6, 9]

*Valerianacapitata* Pall. [1]

*Valerianadubia* Bunge [1, 3, 6, 7, 14]

*Valerianamartjanovi* Krylov [= *Valerianasaichanensis* Kom.] [13]

*Valerianaofficinalis* L. [1, 2, 3, 4, 8, 9]

*Valerianapetrophila* Bunge [1, 3, 6, 7, 13]

SE*Valerianatangutica* Batalin [16]

SE*Valerianatransjenisensis* Kreyer [1, 3, 7]

**39. Caryophyllaceae** Juss. (20 genera and 97 taxa)

*Acanthophyllumpungens* Boiss. [6, 7, 14]

*Arenarialeptoclados* Guss. [7]

*Arenariaserpyllifolia* L. [7, 14]

*Cerastiumalpinum* L. [6]

*Cerastiumarvense* L. [1–10, 13, 14]

*Cerastiumcerastoides* (L.) Britton [≡ *Dichodoncerastoides* (L.) Rchb. ≡ *Stellariacerastoides* L.] [1–7, 10, 14]

*Cerastiumdavuricum* Fisch. [2, 4, 7, 14]

*Cerastiumfalcatum* (Gren.) Bunge [≡ *Stellariafalcata* Ser.] [14]

*Cerastiumholosteoides* Fr. [≡ *Cerastiumfontanumf.holosteoides* (Fr.) M.B.Wyse Jacks.] [2]

*Cerastiumlithospermifolium* Fisch. [1, 3, 6, 7, 10, 13]

*Cerastiummaximum* L. [13]

*Cerastiumpauciflorum* Steven [1, 2, 3, 6, 7]

*Cerastiumpusillum* Ser. [1, 2, 3, 6, 7, 14]

*Cherleriaarctica* (Steven) A.J.Moore & Dillenb. [≡ *Minuartiaarctica* (Steven) Graebn.] [1, 2, 3, 4, 6, 7]

*Cherleriabiflora* (L.) A.J.Moore & Dillenb. [≡ *Minuartiabiflora* (L.) Schinz & Thell.] [1, 2, 3, 6, 7]

*Dianthuschinensis* L. [= *Dianthusversicolor* Fisch.] [1–11, 13]

Dianthuscrinitussubsp.soongoricus (Schischk.) Kozhevn. [≡ *Dianthussoongoricus* Schischk.] [7, 14]

*Dianthusramosissimus* Pall. [10]

*Dianthusrepens* Willd. [≡ Dianthuschinensissubsp.repens (Willd.) Vorosch.] [6, 7]

*Dianthussuperbus* L. [1–10, 13]

*Eremogoneandrosacea* (Grubov) Ikonn. [≡ *Arenariaandrosacea* Grubov] [13]

*Eremogoneasiatica* (Schischk.) Ikonn. [≡ *Arenariaasiatica* Schischk.] [7]

*Eremogonecapillaris* (Poir.) Fenzl [≡ *Arenariacapillaris* Poir.] [1–10, 12, 13]

*Eremogonejuncea* (M.Bieb.) Fenzl [≡ *Arenariajuncea* M.Bieb.] [4, 5, 9]

*Eremogonemeyeri* (Fenzl) Ikonn. [≡ *Arenariameyeri* Fenzl] [2–4, 6, 7, 9, 10, 12, 13]

SE*Eremogonemongolica* (Schischk.) Ikonn. [≡ *Arenariamongolica* Schischk.] [7]

SE*Gymnocarposprzewalskii* Maxim. [≡ *Paronychiaprzewalskii* (Bunge) Rohweder & Urmi-König] [12, 14, 16]

*Gypsophilaaltissima* L. [7]

*Gypsophilacapituliflora* Rupr. [7, 13, 14, 15]

*Gypsophilacephalotes* (Schrenk) F.N.Williams [6, 7]

*Gypsophiladavurica* Fenzl [≡ Gypsophilapatriniisubsp.davurica (Fenzl) Kozhevn.] [2–5, 8, 9, 13]

*Gypsophilapaniculata* L. [3, 4, 7, 10]

*Gypsophilapatrinii* Ser. [1, 3, 4, 6–8, 10, 11]

*Gypsophilaperfoliata* L. [10]

*Gypsophilasericea* (Ser.) Krylov [≡ *Arenariasericea* Ser.] [7]

*Herniariacaucasica* Rupr. [7]

*Herniariaglabra* L. [7]

*Heterochroadesertorum* Bunge [≡ *Gypsophiladesertorum* Fenzl] [1–4, 6–13, 16]

*Lepyrodiclisholosteoides* (C.A.Mey.) Fenzl [≡ *Gouffeiaholosteoides* C.A.Mey.] [3, 10]

*Moehringialateriflora* (L.) Fenzl [≡ *Arenarialateriflora* L.] [1–5, 7, 9, 13]

*Moehringiaumbrosa* (Bunge) Fenzl [≡ *Arenariaumbrosa* Bunge] [1, 2, 6, 7]

*Pseudocherlerialaricina* (L.) Dillenb. & Kadereit [≡ *Minuartialaricina* Mattf.] [4, 5]

*Pseudostellariarupestris* (Turcz.) Pax [1–4, 7, 13]

*Sabulinaregeliana* (Trautv.) Dillenb. & Kadereit [= *Minuartiaregeliana* (Trautv.) Mattf.] [3]

*Sabulinastricta* (Sw.) Rchb. [≡ *Minuartiastricta* (Sw.) Hiern] [1, 2, 3]

*Sabulinaverna* Rchb. [≡ *Minuartiaverna* (L.) Hiern] [1–3, 6, 7, 14]

*Saginasaginoides* (L.) H.Karst. [7]

*Saponariafloribunda* (Kar. & Kir.) Boiss. [≡ *Psammophiliellafloribunda* (Kar. & Kir.) Ikonn.] [14]

*Silenealexandrae* B.Keller [14]

*Silenealtaica* Pers. [7, 13, 14]

*Sileneaprica* Turcz. [≡ *Ussuriaaprica* (Turcz.) Tzvelev] [1–5, 7–10, 12, 13]

*Sileneborysthenica* (Gruner) Walters [3, 10]

*Silenebungei* Bocquet [1, 2, 3, 6]

*Silenechamarensis* Turcz. [≡ Silenetenuissubsp.chamarensis (Turcz.) Kozhevn.] [1–3, 6, 7, 9, 10, 12, 13]

*Sileneconoidea* L. [7]

*Silenefoliosa* Maxim. [4, 12, 13]

*Silenegraminifolia* Otth [= *Silenesobolevskajae* Czerep.] [2, 6, 7, 10, 14]

*Silenegubanovii* Lazkov [6, 7, 13, 14]

SE*Sileneintramongolica* Lazkov [7, 14]

*Silenejeniseensis* Willd. [= *Sileneiche-bogdo* Grubov] [1–6, 8, 9, 13]

Silenelatifoliasubsp.alba (Mill.) Greuter & Burdet [≡ *Lychnisalba* Mill.] [7]

E *Silenemongolica* Maxim. [10, 13]

*Silenequadriloba* Turcz. [2, 3, 7, 10, 14]

*Silenerepens* Patrin [1–10, 12–14]

*Silenesamojedorum* (Sambuk) Oxelman [≡ Lychnissibiricasubsp.samojedorum Sambuk.] [1, 2, 3, 4, 5, 9]

*Silenesibirica* Pers. [14]

*Silenesongarica* (Fisch., C.A.Mey. & Avé-Lall.) Bocquet [= *Gastrolychnisbrachypetala* (Hornem.) Tolm. & Kozhanczikov] [1–7, 9, 12, 13]

*Silenesuaveolens* Kar. & Kir. [≡ *Carpophorasuaveolens* (Kar. & Kir.) Tzvelev ≡ *Melandriumsuaveolens* (Kar. & Kir.) Schischk,] [7, 10, 14]

*Sileneuralensis* (Rupr.) Bocquet [1–3, 6, 7, 10, 13, 14]

*Sileneviolascens* (Tolm.) V.V.Petrovsky & Elven [≡ *Gastrolychnisviolascens* Tolm.] [7]

*Sileneviscosa* Schleich. [3, 7, 10, 14]

*Silenevulgaris* (Moench) Garcke [≡ *Behenvulgaris* Moench [2, 4, 6, 7]

*Spergulariamarina* (L.) Besser [≡ Arenariarubravar.marina L.] [4, 5, 7, 10–15]

*Spergulariasegetalis* G.Don [14]

*Stellariaalsinoides* Boiss. & Buhse [7, 14]

*Stellariaamblyosepala* Schrenk [7, 10–16]

*Stellariabrachypetala* Bunge [= *Stellariaalatavica* Popov, Stellariabrachypetalavar.alatavica (Popov) Kozhevn.] [1, 3–7, 9, 11, 13, 14]

*Stellariabungeana* Fenzl [≡ *Hylebiabungeana* (Fenzl) Tzvelev] [2, 3, 6, 7, 9]

*Stellariacherleriae* (Fisch.) F.N.Williams [≡ *Arenariacherleriae* Fisch.] [1–9, 13]

*Stellariacrassifolia* Ehrh. [1–11, 14]

*Stellariadavurica* Willd. [1–4, 7, 14]

*Stellariadepressa* Schmid [7]

*Stellariadichotoma* L. [1–14]

Stellariadichotomavar.lanceolata Bunge [= *Stellariagypsophiloides* Fenzl] [3, 7–9, 11–13, 15, 16]

*Stellariadiscolor* Turcz. [4, 5, 9]

*Stellariafilicaulis* Makino [2, 3]

*Stellariaimbricata* Bunge [6, 7, 14]

*Stellariairrigua* Bunge [1–4, 6, 7, 13]

*Stellarialongifolia* Muhl. [1, 2, 3, 4, 5, 9]

*Stellarialongipes* Goldie [= *Stellularialongipes* (Goldie) MacMill.] [1–3, 5–7, 9]

*Stellariamartjanovii* Krylov [= *Mesostemmamartjanovii* (Krylov) Ikonn.] [7]

*Stellariamedia* (L.) Vill. [= *Alsinemedia* L.] [2, 3]

*Stellariapalustris* Ehrh. [2, 3, 7, 9]

*Stellariapetraea* Bunge [1–4, 6, 7, 13]

*Stellariapulvinata* Grubov [6, 7]

*Stellariaradians* L. [5, 9]

*Stellariazolotuchinii* A.L.Ebel [≡ *Stellariaglandulifera* N.Zolot. nom. illegit.] [3, 10]

**40. Celastraceae** R.Br. (2 genera and 3 species)

*Euonymusmaackii* Rupr. [5, 9]

*Parnassialaxmannii* Pall. [1–4, 6, 10]

*Parnassiapalustris* L. [1–11, 14]

**41. Ceratophyllaceae** Gray (1 genus and 2 taxa)

*Ceratophyllumdemersum* L. [1, 4, 8–10, 14]

Ceratophyllumplatyacanthumsubsp.oryzetorum (Kom.) Les [10]

**42. Cleomaceae** Bercht. & J.Presl (1 genus and 1 species)

E *Cleomegobica* Grubov [15]

**43. Convolvulaceae** Juss. (4 genera and 15 species)

*Calystegiahederacea* Wall. [12, 13]

*Calystegiapellita* G.Don [= *Calystegiadahurica* Herb.] [1, 3, 4]

*Calystegiasepium* (L.) R.Br. [≡ *Convolvulussepium* L.] [14]

*Calystegiasubvolubilis* G.Don [2, 3]

*Convolvulusammannii* Desr. [2, 3, 4, 6–14, 16]

*Convolvulusarvensis* L. [2, 3, 4, 7–16]

*Convolvulusfruticosus* Pall. [7, 10–16]

*Convolvulusgortschakovii* Schrenk [7, 8, 10, 11, 13–16]

*Convolvulustragacanthoides* Turcz. [12, 16]

*Cuscutaaustralis* R.Br. [9]

*Cuscutachinensis* Lam. [4, 9, 12, 13, 15, 16]

*Cuscutaeuropaea* L. [3–7, 9, 13, 14]

*Cuscutalupuliformis* Krock. [6, 7, 10, 14]

*Cuscutamonogyna* Vahl [≡ *Monogynellamonogyna* (Vahl) Hadač] [4, 5, 8, 14]

*Merremiasibirica* (L.) Hallier f. [3]

**44. Cornaceae** Bercht. & J.Presl (1 genus and 1 species)

*Cornusalba* L. [1, 2, 3, 4, 5, 9]

**45. Crassulaceae** J.St.-Hil. (6 genera and 17 taxa)

*Crassulaaquatica* (L.) Schönland [2, 4, 6, 10]

*Hylotelephiumewersii* (Ledeb.) H.Ohba [≡ *Sedumewersii* Ledeb.] [6, 7, 14]

*Hylotelephiumpallescens* (Freyn) H.Ohba [2, 3, 4, 5]

*Hylotelephiumtelephium* (L.) H.Ohba [≡ *Sedumtelephium* L.] [1–10]

*Orostachysfimbriata* (Turcz.) A.Berger [≡ *Cotyledonfimbriata* Turcz.] [2–6, 8–13]

*Orostachysmalacophylla* (Pall.) Fisch. [≡ *Cotyledonmalacophylla* Pall.] [1–5, 8, 9]

*Orostachysspinosa* (L.) Sweet [≡ *Cotyledonspinosa* L.] [1–4, 6–15]

*Orostachysthyrsiflora* Fisch. [1, 3–11, 13–15]

*Phedimusaizoon* (L.) ‘t Hart [≡ *Sedumaizoon* L.] [1–14]

*Phedimushybridus* (L.) ‘t Hart [≡ *Sedumhybridium* L.] [1, 3–9, 12]

*Pseudosedumlievenii* A.Berger [7, 14]

*Rhodiolaalgida* (Ledeb.) Fisch. & C.A.Mey. [6, 7]

*Rhodiolacoccinea* (Royle) Boriss. [7]

*Rhodiolalitwinowii* Boriss. [3, 6, 7, 10]

*Rhodiolaquadrifida* (Pall.) Fisch. & C.A.Mey. [1–3, 6, 7, 13]

*Rhodiolarosea* L. [≡ *Sedumroseum* (L.) Scop.] [1–8, 13, 14]

*Rhodiolastephani* (Cham.) Trautv. & C.A.Mey. [= *Rhodiolakrylovii* Polozhij & Revjakina = *Rhodiolapinnatifida* Boriss. = *Rhodiolasubpinnata* (Krasnob.) Krasnob.] [1, 2, 6, 7, 10]

**46. Cynomoriaceae** Endl. (1 genus and 1 species)

*Cynomoriumsongaricum* Rupr. [≡ Cynomoriumcoccineumsubsp.songaricum (Rupr.) J.Léonard] [10–16]

**47. Cyperaceae** Juss. (10 genera and 131 taxa)

Note: To date, eight species of *Kobresia* Willd. have been recorded in Mongolia (Nyambayar 2009b; Urgamal et al. 2014), and they were recently transferred to the genus *Carex* L. (Global Carex Group 2015).

BlysmuscompressusL.subsp.brevifolius (Decne.) Kukkonen [2–5, 7–9, 11, 13, 14]

*Blysmusrufus* Link [1, 3–15]

*Bolboschoenusmaritimus* (L.) Palla [≡ *Scirpusmaritimus* L.] [14, 16]

Bolboschoenusmaritimussubsp.affinis (Roth) T.Koyama [= *Bolboschoenuspopovii* T.V.Egorova] [10–16]

*Bolboschoenusplaniculmis* (F.Schmidt) T.V.Egorova [4, 5, 8–12, 15]

*Carexaccrescens* Ohwi [= *Carexpallida* C.A.Mey.] [2]

*Carexacuta* L. [2, 3, 14]

*Carexalatauensis* S.R.Zhang [= *Kobresiahumilis* (C.A.Mey.) Serg.] [3, 6, 7, 11, 13]

*Carexalba* Scop. [1, 2]

*Carexaltaica* (Gorodkov) V.I.Krecz. [≡ Carexorbicularissubsp.altaica (Gorodkov) T.V.Egorova] [3]

*Carexamgunensis* F.Schmidt [= *Carexchloroleuca* Meinsh.] [1, 2, 3, 5, 7]

*Carexappendiculata* Kük. [2, 4, 5, 8–10]

*Carexargunensis* Turcz. [≡ Carexrupestrissubsp.argunensis (Turcz.) Vorosch.] [2, 4, 5, 7, 9]

*Carexarnellii* Christ [2, 3, 4, 5, 9]

*Carexaterrima* Hoppe [1, 2, 3, 6, 7]

*Carexatherodes* Spreng. [1–5, 7, 8, 9, 11]

*Carexatrofusca* Schkuhr [1, 3, 7]

Carexbigelowiisubsp.ensifolia (Gorodkov) Holub [1, 2, 3, 6, 7]

Carexbigelowiisubsp.rigidioides (Gorodkov) T.V.Egorova [2, 3]

*Carexbistaminata* (W.Z.Di & M.J.Zhong) S.R.Zhang [≡ *Kobresiabistaminata* W.Z.Di & M.J.Zhong = *Kobresiamyosuroides* (Vill.) Fiori] [1–3, 5–7, 10, 13, 14]

*Carexbohemica* Schreb. [4]

*Carexborealipolaris* S.R.Zhang [= *Kobresiasibirica* (Turcz.) Boeck. = *Kobresiasmirnovii* Ivanova] [1, 2, 3, 6, 7, 14]

*Carexbrunnescens* (Pers.) Poir. [≡ Carexcurtavar.brunnescens Pers.] [2, 9]

*Carexcanescens* L. [2, 4, 7]

*Carexcapillifolia* (Decne.) S.R.Zhang [= *Kobresiacapilliformis* Ivanova] [3, 7]

*Carexcapitata* L. [1, 2, 3, 7]

*Carexcapricornis* Meinsh. [10]

*Carexcaryophyllea* Latourr. [1–4, 6, 7, 8, 13]

*Carexcespitosa* L. [1–10, 15]

*Carexchordorrhiza* L.f. [2]

*Carexcoriophora* Fisch. & C.A.Mey. [1–5, 8, 9]

*Carexcuraica* Kunth [1–3, 6, 7, 10, 11, 14]

*Carexdahurica* Kük. [1, 2]

*Carexdelicata* C.B.Clarke [1–10, 13]

*Carexdiandra* Schrank [2, 5, 9, 10]

*Carexdiluta* M.Bieb. [10]

Carexdistanssubsp.aspratilis (V.I.Krecz.) T.V.Egorova [4, 10]

*Carexduriuscula* C.A.Mey. [1–14, 16]

*Carexeleusinoides* Turcz. [2, 3]

*Carexenervis* C.A.Mey. [1–13, 15]

*Carexeremopyroides* V.I.Krecz. [9, 11]

*Carexericetorum* Pollich [4]

*Carexglobularis* L. [1, 2, 7]

*Carexgotoi* Ohwi [≡ Carexsongoricasubsp.gotoi (Ohwi) Popov] [4, 5, 9]

*Carexhancockiana* Maxim. [2, 3, 5]

*Carexheterolepis* Bunge [5]

SE*Carexiljinii* V.I.Krecz. [1, 2, 3]

*Carexkaroi* Freyn [= *Carexselengensis* N.A.Ivanova] [2, 3, 4, 5, 9, 10]

*Carexkorshinskii* Kom. [1–6, 8–10, 12, 13]

*Carexlachenalii* Schkuhr [1, 7, 14]

*Carexlanceolata* Boott [2]

*Carexlasiocarpa* Ehrh. [2]

*Carexlaxa* Wahlenb. [1, 2]

*Carexledebouriana* C.A.Mey. [≡ Carexcapillarissubsp.ledebouriana (C.A.Mey.) Vorosch.] [1, 2, 3, 6, 7]

*Carexleporina* L. [2, 3, 7]

*Carexlimosa* L. [2]

*Carexlithophila* Turcz. [≡ Carexdistichasubsp.lithophila (Turcz.) D.Hämet-Ahti] [2–5, 7, 9, 10]

*Carexloliacea* L. [2, 5]

*Carexmacrogyna* Turcz. [1, 3, 6, 7, 13]

*Carexmacroprophylla* (Y.C.Yang) S.R.Zhang [≡ *Kobresiafilifolia* (Turcz.) C.B.Clarke ≡ Kobresiafilifolia(Turcz.)C.B.Clarkevar.macroprophylla Y.C.Yang] [1–4, 6–9, 13]

CarexmagellanicaLam.subsp.irrigua (Wahlenb.) Hiitonen [≡ CarexlimosaL.var.irrigua Wahlenb.] [2, 3]

*Carexmedia* R.Br. [1–4, 6, 7, 10]

*Carexmelanantha* C.A.Mey. [1–3, 6, 7, 9, 13]

*Carexmelanocephala* Turcz. [1, 3, 7]

*Carexmeyeriana* Kunth [1, 2, 3, 5]

*Carexmicroglochin* Wahlenb. [1–4, 6–8, 11, 14]

Carexnigrasubsp.juncea (Fr.) Soó [= *Carexjuncella* T.M.Fries] [1, 3]

*Carexnorvegica* Retz. [1, 2, 3, 4, 6, 7]

*Carexobtusata* Lilj. [1–3, 6, 7, 9, 13]

*Carexorbicularis* Boott [1–8, 12–15]

Carexpamirica(O.Fedtsch.)B.Fedtsch.subsp.dichroa (Freyn) T.V.Egorova [≡ CarexpullaGooden.subsp.dichroa Freyn] [1–4, 6, 7, 10]

CarexparallelaLaest.subsp.redowskiana (C.A.Mey.) T.V.Egorova [≡ *Carexredowskiana* C.A.Mey.] [1, 7]

*Carexparva* Nees [3]

Carexpediformisvar.macroura (Meinsh.) Kük. [≡ *Carexmacroura* Meinsh.] [1, 2, 3, 5, 7]

Carexpediformissubsp.pediformis [1–9, 13, 14]

*Carexpraecox* Schreb. [2, 4]

*Carexpseudofoetida* Kük. [7, 13]

*Carexpycnostachya* Kar. & Kir. [≡ Carexcuraicasubsp.pycnostachya (Kar. & Kir.) T.V.Egorova] [3, 11, 14]

*Carexraddei* Kük. [2]

*Carexrelaxa* V.I.Krecz. [3, 4, 8, 9]

*Carexreptabunda* (Trautv.) V.I.Krecz. [1, 3–5, 8, 9, 11, 12, 16]

*Carexrhynchophysa* Fisch. [2–5, 9, 10]

*Carexrostrata* Stokes [1–5, 8, 9, 10]

*Carexrupestris* All. [1, 2, 3, 6, 7, 13]

*Carexsabulosa* Turcz. [1–4, 7, 8, 10]

*Carexsabynensis* Less. [2, 3]

SE*Carexsajanensis* V.I.Krecz. [1–4, 6–9, 11, 12]

*Carexsargentiana* (Hemsl.) S.R.Zhang [≡ *Kobresiasargentiana* Hemsl. = *Kobresiarobusta* Maxim.] [3]

*Carexsaxatilis* L. [= Carexsaxatilissubsp.laxa (Trautv.) Kalela] [1, 3]

*Carexschmidtii* Meinsh. [2, 3, 4, 5, 8, 9]

*Carexsedakowii* C.A.Mey. [1, 2, 4]

*Carexsimpliciuscula* Wahlenb. [= Kobresiasimpliciusculasubsp.subholarctica T.V.Egorova] [1, 2, 3, 7, 12, 14]

*Carexsongorica* Kar. & Kir. [4, 5, 9, 10, 14]

*Carexsordida* Van Heurck & Müll.Arg. [1, 2, 3, 6, 7, 8, 13, 14]

Carexstenophyllasubsp.stenophylloides (V.I.Krecz.) T.V.Egorova [3, 7–16]

*Carexsupermascula* V.I.Krecz. [1, 2, 4, 5, 9, 13]

*Carextenuiflora* Wahlenb. [1, 2, 3]

*Carextomentosa* L. [2, 4, 8]

Carextristissubsp.stenocarpa (Turcz.) T.V.Egorova [1–3, 6–8, 13, 14]

Carexvaginatavar.petersii (C.A.Mey.) Akiyama [= *Carexfalcata* Turcz.] [2, 3]

CarexvaginataTauschvar.vaginata [1, 2, 3]

*Carexvesicata* Meinsh. [1–6, 8, 9, 14]

*Carexwilliamsii* Britton [2]

*Carexyamatsutana* Ohwi [= *Carexdiplasiocarpa* V.I.Krecz.] [2, 5, 9]

*Cyperusfuscus* L. [8, 9, 10]

*Cyperushamulosus* M.Bieb. [≡ *Mariscushamulosus* (M.Bieb.) S.S.Hooper] [10]

*Cyperusmichelianus* (L.) Delile [≡ *Scirpusmichelianus* L.] [4]

*Cyperuspannonicus* Jacq. [≡ *Juncelluspannonicus* (Jacq.) C.B.Clarke] [10, 11, 16]

*Eleocharisacicularis* (L.) Roem. & Schult. [2–4, 6–9, 11, 14]

*Eleocharismamillata* (H.Lindb.) H.Lindb. [10]

*Eleocharismitracarpa* Steud. [7, 14, 15]

*Eleocharispalustris* (L.) Roem. & Schult. [≡ *Scirpuspalustris* L.] [1–16]

*Eleocharisquinqueflora* (Hartmann) O.Schwarz [≡ *Scirpusquinqueflorus* Hartmann] [2–4, 7, 8, 10, 11, 15]

*Eleocharisuniglumis* Schult. [= *Eleocharisklingei* (Meinsh.) B.Fedtsch.] [1, 3–5, 8–10, 12–15]

*Eleocharisyokoscensis* (Franch. & Sav.) Tang & F.T.Wang [≡ Eleocharisacicularissubsp.yokoscensis (Franch. & Sav.) T.V.Egorova] [2–5, 8, 9, 14]

*Eriophorumaltaicum* Meinsh. [1, 2, 3, 6, 7]

*Eriophorumangustifolium* Honck. [1–7, 9, 10, 11]

Eriophorumangustifoliumsubsp.komarovii (V.N.Vassil.) M.S.Novos. [1–10]

*Eriophorumbrachyantherum* Trautv. & C.A.Mey. [1–4, 6, 7, 10]

*Eriophorumcallitrix* C.A.Mey. [1]

*Eriophorumchamissonis* C.A.Mey. [= *Eriophorummandshuricum* Meinsh.] [1, 3, 4, 5, 7]

*Eriophorumgracile* W.D.J.Koch [= Eriophorumgracilesubsp.asiaticum (V.N.Vassil.) M.S.Novos.] [3, 4]

*Eriophorumhumile* Turcz. [1, 2, 3, 6, 7]

*Eriophorumvaginatum* L. [3]

*Schoenoplectiellasupina* (L.) Lye [= *Schoenoplectussupinus* (L.) Pall.] [10]

Schoenoplectuslacustrissubsp.hippolytii (V.I.Krecz.) Kukkonen [≡ *Scirpushippolyti* V.I.Krecz.] [1–10, 12–15]

*Schoenoplectustabernaemontani* (C.C.Gmel.) Pall. [2, 3, 14]

*Schoenoplectustriqueter* (L.) Palla [≡ *Scirpustriqueter* L.] [9]

*Scirpusorientalis* Ohwi [≡ Scirpussylvaticussubsp.orientalis (Ohwi) Vorosch.] [2, 3, 4, 5, 9]

*Scirpusradicans* Schkuhr [2, 3, 4, 5, 9]

*Trichophorumpumilum* (Vahl) Schinz & Thell. [1, 3, 4, 8, 9, 10, 14]

**48. Droseraceae** Salisb. (2 genera and 3 species)

*Aldrovandavesiculosa* L. [10]

*Droseraanglica* Huds. [2]

*Droserarotundifolia* L. [2]

**49. Elaeagnaceae** Juss. (2 genera and 3 taxa)

*Elaeagnusangustifolia* L. [15, 16]

HippophaerhamnoidesSt.-Lag.subsp.mongolica Rousi [≡ *Hippophaemongolica* (Rousi) Tzvelev] [3, 4, 6, 7, 10, 11, 13]

HippophaerhamnoidesSt.-Lag.subsp.turkestanica Rousi [≡ *Hippophaeturkestanica* (Rousi) Tzvelev] [14]

**50. Ericaceae** Durande (12 genera and 27 taxa)

*Arctostaphylosuva-ursi* (L.) Spreng. [2]

*Arctousalpina* (L.) Nied. [= *Arbutusalpina* L.] [1, 3, 6, 10]

*Cassiopeericoides* D.Don [4]

*Chamaedaphnecalyculata* (L.) Moench [1–3, 6, 7, 13]

EmpetrumnigrumL.subsp.nigrum [1, 2, 3, 4]

Empetrumnigrumsubsp.sibiricum (V.N.Vassil.) Kuvaev [≡ *Empetrumsibiricum* V.N.Vassil.] [1, 2, 3, 4, 6, 7]

*Monesesuniflora* A.Gray [1, 2, 4, 7]

*Monotropahypopitys* L. [1, 3, 4]

*Orthiliaobtusata* (Turcz.) H.Hara [≡ Pyrolasecundavar.obtusata Turcz.] [1, 2, 3, 4, 6, 7]

*Orthiliasecunda* (L.) House [≡ *Pyrolasecunda* L] [1, 2, 3, 4, 7]

*Phyllodocecaerulea* (L.) Bab. [≡ *Andromedacaerulea* L.] [1]

Pyrolaasarifoliasubsp.incarnata (DC.) A.E.Murray [≡ Pyrolarotundifoliavar.incarnata (DC.) A.P.Khokhr.] [1–7, 9]

*Pyrolachlorantha* Sw. [1, 4]

*Pyroladaurica* Kom. [1, 4, 5, 7, 9]

*Pyrolamedia* Sw. [3, 4]

*Pyrolaminor* L. [2]

*Pyrolarotundifolia* L. [1, 2, 3, 4, 7]

*Rhododendronadamsii* Rehder [1, 3]

*Rhododendronaureum* Georgi [1, 2]

*Rhododendrondauricum* L. [1, 2, 3, 4, 5]

*Rhododendronlapponicum* (L.) Wahlenb. [1, 2, 3]

*Rhododendronledebourii* Pojark. [≡ Rhododendrondauricumsubsp.ledebourii (Pojark.) Alexandrova & P.A.Schmidt] [1, 3]

*Rhododendrontomentosum* Harmaja [≡ *Ledumpalustre* L., = Ledumpalustrevar.decumbens Aiton] [1, 2, 3, 4]

*Vacciniummicrocarpum* (Turcz.) Schmalh. [≡ *Oxycoccusmicrocarpus* Turcz.] [1, 2]

*Vacciniummyrtillus* L. [≡ *Vitis-idaeamyrtillus* (L.) Moench] [1, 2]

*Vacciniumuliginosum* L. [1, 2, 3, 4, 6]

*Vacciniumvitis-idaea* L. [≡ *Vitis-idaeavitis-idaea* (L.) Britton] [1, 2, 3, 4, 5, 6]

**51. Euphorbiaceae** Juss. (1 genus and 15 species)

*Euphorbiaalpina* C.A.Mey. [7]

*Euphorbiacaesia* Kar. & Kir. [7]

*Euphorbiaesula* L. [= *Euphorbiadiscolor* Ledeb.] [1–5, 8, 9, 12]

*Euphorbiafischeriana* Steud. [4, 5, 9]

*Euphorbiahumifusa* Willd. [3, 4, 7–16]

*Euphorbiakozlovii* Prokh. [8, 10, 12, 13, 16]

*Euphorbiamacrorhiza* Ledeb. [6, 7]

*Euphorbiamongolica* Prokh. [3, 6, 7, 10–13]

*Euphorbiapachyrhiza* Kar. & Kir. [7]

*Euphorbiapilosa* L. [7]

*Euphorbiapotaninii* Prokh. [3, 6, 7, 10, 13]

*Euphorbiasoongarica* Boiss. [≡ *Galarhoeussoongaricus* (Boiss.) Prokh.] [7]

*Euphorbiasubcordata* C.A.Mey. [3, 6, 7, 10, 14]

*Euphorbiatshuiensis* (Prokh.) Serg. [6, 7, 10]

*Euphorbiavirgata* Waldst. & Kit. [≡ *Galarhoeusvirgatus* (Waldst. & Kit.) Prokh.] [4, 8]

**52. Fabaceae** Lindl. (24 genera and 328 taxa)

*Alhagimaurorum* Medik. [14, 15, 16]

Alhagipseudalhagisubsp.kirghisorum (Schrenk) Yakovl. [= *Alhagisparsifolia* Shap.] [14, 15]

SE*Ammopiptanthusmongolicus* (Maxim.) S.H.Cheng [14, 15, 16]

SE*Astragalusadmirabilus* Pjak & E.Pjak [7]

*Astragalusadsurgens* Pall. [1–13]

*Astragalusagrestis* Douglas ex G.Don [7, 10]

*Astragalusaksaicus* Schischk. [7]

SE*Astragalusalaschanus* Bunge [13]

*Astragalusalberti* Bunge [10, 11]

*Astragalusalbicans* Bong. [14]

*Astragalusalpinus* L. [1, 2, 7, 9, 13]

*Astragalusaltaicola* Podlech [6, 7, 14]

*Astragalusammodytes* Pall. [10, 14]

*Astragalusankylotus* Fisch. & C.A.Mey. [7, 14]

*Astragalusarcuatus* Kar. & Kir. [14]

*Astragalusargutensis* Bunge [4, 6, 7, 8]

*Astragalusarkalycensis* Bunge [6, 7, 14]

*Astragalusaustro-sibiricus* Schischk. [1, 3, 6, 7, 8, 13]

SE*Astragalusbaitagensis* Sanchir [14]

*Astragalusbeketowii* (Krassn.) B.Fedtsch. [6]

*Astragalusborodinii* Krasnob. [7, 14]

*Astragalusbrachybotrys* Bunge [6, 7, 10–14]

*Astragalusbrevifolius* Ledeb. [1–4, 6–8, 10–13]

SE*Astragalusburtschumensis* Sumnev. [6, 7, 10]

*Astragaluscandidissimus* Ledeb. [7, 14]

E *Astragaluschamonobrychis* Podlech [7]

E *Astragaluschangaicus* Sanchir [3, 7]

*Astragaluschinensis* L.f. [5, 9]

SE*Astragaluschorinensis* Bunge [= *Astragaluspseudochorinensis* N.Ulziykh.] [2, 3, 4]

E *Astragaluschubsugulicus* Gontsch. ex N.Ulziykh. [1]

*Astragaluscompressus* Ledeb. [7]

*Astragalusconfertus* Benth. [3, 7]

*Astragalusconsanguineus* Bong. & C.A.Mey. [10]

*Astragaluscontortuplicatus* L. [14]

*Astragalusdahuricus* Patrin [3–5, 8–10, 12]

*Astragalusdanicus* Retz. [1, 4]

*Astragalusdepauperatus* Ledeb. [3, 6, 7]

*Astragalusdilutus* Bunge [3, 6, 7, 10, 12–14]

*Astragalusdschimensis* Gontsch. [7, 14]

*Astragalusellipsoideus* Ledeb. [3, 6, 7, 10, 12, 14–16]

*Astragalusfiliformis* (DC.) Poir. [≡ *Oxytropisfiliformis* DC.] [1–5, 7–11, 13]

*Astragalusfollicularis* Pall. [3]

*Astragalusfrigidus* A.Gray [1–3, 6, 7, 13]

*Astragalusfruticosus* Pall. [2, 3, 4, 9, 13]

*Astragalusgalactites* Pall. [1–5, 8, 9]

*Astragalusglomeratus* Ledeb. [3, 7, 13]

E *Astragalusgobicus* Hanelt & Davaz. [14, 15]

E *Astragalusgranitovii* Sanchir [7, 14]

SE*Astragalusgregorii* B. Fedtsch. & Basil. [7]

SE*Astragalusgrubovii* Sanchir [= *Astragalusalaschanensis* H.C.Fu] [7, 10–16]

SE*Astragalusgrum-grshimailoi* Palib. [7]

E *Astragalusgubanovii* N.Ulziykh. [7, 10]

SE*Astragalushabaheensis* Y.X.Liou [14]

SE*Astragalushamiensis* S.B.Ho [= *Astragalusbanzragczii* N.Ulziykh.] [14]

SE*Astragalushsinbaticus* P.Y.Fu & Y.A.Chen [= *Astragalusquasitesticulatus* Barratte & Z.Y.Chu] [9]

*Astragalushypogaeus* Ledeb. [3, 6, 7, 10]

*Astragalusinopinatus* Boriss. [1–5, 7–9, 13]

SE*Astragalusjunatovii* Sanchir [12, 13, 15, 16]

*Astragaluskasachstanicus* Golosk. [7]

*Astragaluskaufmannii* Krylov [1, 3]

E *Astragaluskenteicus* N.Ulziykh. [2]

*Astragalusklementzii* N.Ulziykh. [3]

E *Astragaluskoslovii* B.Fedtsch. & N.Basil. [13]

*Astragaluskurtschumensis* Bunge [7, 10]

*Astragaluslaguroides* Pall. [= *Astragalusgobi-altaicus* N.Ulziykh.] [2–4, 6–8, 10–13]

*Astragaluslasiopetalus* Bunge [7, 14]

*Astragaluslaxmannii* Jacq. [7, 10, 14]

*Astragaluslepsensis* Bunge [7]

*Astragalusleptostachys* Pall. [= *Astragalusmacropterus* DC. = *Astragalusmulticaulis* Ledeb.] [1, 3, 6, 7, 13]

SE*Astragaluslupulinus* Pall. [3, 4, 7, 11–14, 16]

SE*Astragalusluxurians* Bunge [7]

*Astragalusmacrolobus* M.Bieb.[= *Astragalusmacrocerus* C.A.Mey.] [7, 10, 11]

*Astragalusmacrotrichus* E.Peter [7, 10, 12, 14–16]

*Astragalusmajevskianus* Krylov [7]

*Astragalusmegalanthus* DC. [8, 12]

*Astragalusmelilotoides* Pall. [2, 3, 4, 8–13, 16]

*Astragalusminiatus* Bunge [3, 4, 8, 9, 11–13]

*Astragalusmongholicus* Bunge [= *Astragalusmembranaceus* Fisch. = *Astragaluspropinquus* Schischk.] [1–11, 13]

*Astragalusmonophyllus* Bunge [6–16]

*Astragalusnorvegicus* Weber [1, 2]

SE*Astragalusochrias* Bunge [12, 14, 15, 16]

*Astragalusonobrychis* L. [10]

*Astragalusortholobus* Bunge [7]

*Astragalusoxyglottis* Steven [7, 14]

*Astragaluspallasii* Spreng. [= *Astragaluslasiophyllus* Ledeb.] [14]

SE*Astragaluspavlovii* B.Fedtsch. & Basil. [13–16]

*Astragaluspeterae* Tsai & Yu [6, 10]

*Astragalusphysocarpus* Ledeb. [7]

SE*Astragaluspolitovii* Krylov [7]

SE*Astragaluspolozhiae* Timokhina [6, 7]

SE*Astragaluspseudoborodinii* S.B.Ho [= *Astragalusbaischinticus* N.Ulziykh.] [14]

*Astragaluspseudobrachytropis* Gontsch. [6]

E *Astragaluspseudotesticulatus* Sanchir [7]

E *Astragaluspseudovulpinus* Sanchir [14]

*Astragaluspuberulus* Ledeb. [≡ *Craccinapuberula* (Ledeb.) Steven] [7, 10, 11, 13, 14]

*Astragalusroseus* Ledeb. [7, 14]

*Astragalusrudolffii* N.Ulziykh. [7, 14]

*Astragalusrytidocarpus* Ledeb. [2, 3, 7]

*Astragalussabuletorum* Ledeb. [7, 14, 15]

E *Astragalussaichanensis* Sanchir [7, 13]

E *Astragalussanczirii* N.Ulziykh. [7, 14]

SE*Astragalussaralensis* Gontsch. [1]

*Astragalusscaberrimus* Bunge [2–4, 8, 9, 12]

*Astragalusscabrisetus* Bong. [15]

*Astragalusschanginianus* Pall. [7]

*Astragalusschrenkianus* Fisch. & C.A.Mey. [7]

*Astragalusscleropodius* Ledeb. [7]

*Astragalussecundus* DC. [≡ Astragalusfrigidussubsp.secundus (DC.) Vorosch.] [1, 3, 13]

*Astragalussphaerocystis* Bunge [7]

*Astragalusstenoceras* C.A.Mey. [10]

*Astragalussuffruticosus* DC. [1–4, 7, 8, 13]

*Astragalussulcatus* L. [7, 10, 11, 13, 14]

E *Astragalustamiricus* N.Ulziykh. [3]

*Astragalustenuis* Turcz. [≡ AstragalusmelilotoidesPall.var.tenuis (Turcz.) Ledeb.] [1, 2, 3, 5, 8, 9]

*Astragalustephrolobus* Bunge [7]

*Astragalustibetanus* Benth. [7, 10, 11, 14, 15]

*Astragalustschujensis* Bunge [7]

*Astragalustulinovii* B.Fedtsch. [7]

SE*Astragalustuvinicus* Timokhina [7, 14]

*Astragalusuliginosus* L. [1–5, 8]

E *Astragalusulziykhutagii* Sytin [= *Astragalusalexandrii* N.Ulziykh.] [7]

*Astragalusurunguensis* N.Ulziykh. [14]

SE*Astragalusvallestris* Kamelin [3, 7, 10–14]

*Astragalusvariabilis* Bunge [7, 11–16]

*Astragalusversicolor* Pall. [= *Astragalusalexandrii* N.Ulziykh. nom. illegit.] [1, 2, 3, 4, 6]

E *Astragalusviridiflavus* N.Ulziykh. [1, 2, 3, 4]

*Astragalusxanthotrichos* Ledeb. [7]

SE*Astragalusyumenensis* S.B.Ho [14, 15]

SE*Astragaluszacharensis* Bunge [9]

*Astragaluszaissanensis* Sumnev. [7]

*Caraganaarborescens* Lam. [1, 3, 10]

*Caraganabrachypoda* Pojark. [12, 13, 16]

*Caraganabungei* Ledeb. [3, 6, 7, 10, 11, 13–15]

SE*Caraganadavazamcii* Sanchir [≡ Caraganakorshinskiivar.davazamcii (Sanchir) Yakovlev] [9, 11–13, 16]

E *Caraganagobica* Sanczir [7, 12, 13, 14]

*Caraganahalodendron* (Pall.) Dum.Cours. [≡ *Halimodendronhalodendron* (Pall.) Voss.] [7, 10, 14, 15]

*Caraganajubata* Poir. [1, 2, 3, 7, 13]

SE*Caraganakorshinskii* Kom. [9, 11–13, 16]

*Caraganaleucophloea* Pojark. [3, 4, 6–8, 10–16]

*Caraganamicrophylla* Lam. [2, 3, 4, 8, 9]

*Caraganapygmaea* (L.) DC. [≡ *Robiniapygmaea* L.] [1–14]

*Caraganaspinosa* (L.) Vahl [4, 6–8, 10, 11, 14, 16]

*Caraganastenophylla* Pojark. [3–5, 8, 9, 12, 13]

SE*Caraganatibetica* Kom. [12, 13, 16]

*Chesneyaferganensis* Korsh. [≡ *Chesniellaferganensis* (Korsh.) Boriss.] [15]

SE*Chesneyamongolica* Maxim. [10–13, 15, 16]

SE*Chesniellamacrantha* (W.C.Cheng) L.Duan, J.Wen & Zhao Y.Chang [= *Spongiocarpellagrubovii* (N.Ulziykh.) Yakovlev] [15, 16]

*Cicersongaricum* Steph. [7]

*Corethrodendronfruticosum* (Pall.) B.H.Choi & H.Ohashi [≡= *Hedysarumfruticosum* Pall.] [3, 4, 5, 8–13, 16]

*Corethrodendronscoparium* (Fisch. & C.A.Mey.) Fisch. & Basiner [≡ *Hedysarumscoparium* Fisch. & C.A.Mey. = *Hedysarumarbuscula* Maxim.] [15, 16]

*Glycyrrhizaaspera* Pall. [10, 14]

*Glycyrrhizaglabra* L. [= *Glycyrrhizaalaschanica* Grankina] [10, 12, 14, 15, 16]

SE*Glycyrrhizainflata* Batalin [7, 15, 16]

*Glycyrrhizapallidiflora* Maxim. [9]

SE*Glycyrrhizasquamulosa* Franch. [12, 14]

*Glycyrrhizauralensis* Fisch. [= *Glycyrrhizagobica* Grankina = *Glycyrrhizasoongorica* Grankina] [2–5, 8–16]

*Gueldenstaedtiamonophylla* Fisch. [6, 7, 10, 12, 13, 16]

*Gueldenstaedtiaverna* (Georgi) Boriss. [= *Gueldenstaedtiastenophylla* Bunge] [1, 2, 4, 5, 9]

*Hedysarumalpinum* L. [≡ *Echinolobiumalpinum* (L.) Desv.] [1–7, 9]

*Hedysarumaustrosibiricum* B.Fedtsch. [≡ Hedysarumhedysaroidessubsp.austrosibiricum (B.Fedtsch.) Jurtzev] [3, 6, 7]

*Hedysarumbrachypterum* Bunge [2, 9]

SE*Hedysarumchalchorum* N.Ulziykh. [3, 4, 8]

*Hedysarumconsanguineum* DC. [7]

*Hedysarumdahuricum* Turcz. [≡ Hedysarumgmeliniivar.dahuricum (Turcz.) R.Sha] [1, 3–10, 13]

*Hedysarumferganense* Korsh. [1–4, 6–11, 13, 14, 16]

*Hedysarumgmelinii* Ledeb. [1–4, 6, 7, 9, 10, 13]

Hedysarumhedysaroidessubsp.arcticum (B.Fedtsch.) P.W.Ball [≡ *Hedysarumarcticum* B.Fedtsch.] [1, 3, 6, 7]

*Hedysarumiliense* B.Fedtsch. [7]

*Hedysaruminundatum* Turcz. [1–3, 6, 7, 10, 13]

*Hedysarumkamelinii* N.Ulziykh. [7]

*Hedysarumkrylovii* Sumn. [7]

*Hedysarumlintschevskyi* Bajtenov [7, 13]

*Hedysarumneglectum* Ledeb. [1, 2, 3, 6, 7]

*Hedysarumroseum* Sims [2, 3, 4]

*Hedysarumsajanicum* N.Ulziykh. [1]

SE*Hedysarumsangilense* Krasnob. & Timokhina [1, 3]

*Hedysarumsetigerum* Turcz. [2–7, 9]

*Hedysarumtheinum* Krasnob. [7]

*Lathyrushumilis* (Ser.) Fisch. [≡ *Orobushumilis* Ser.] [1–5, 8, 9]

*Lathyrusledebourii* Trautv. [7]

LathyruspalustrisL.subsp.pilosus (Cham.) Hultén [1–5, 8, 9, 10, 14]

*Lathyruspisiformis* L. [5, 9]

*Lathyruspratensis* L. [2, 3, 4, 10]

*Lathyrusquinquenervius* (Miq.) Litv. [3, 4, 5]

*Lespedezabicolor* Turcz. [5]

*Lespedezadaurica* (Laxm.) Schindl. [≡ *Trifoliumdauricum* Laxm.] [2–5, 8, 9, 11–13, 16]

*Lespedezajuncea* (L.f.) Pers. [2–5, 8, 9]

*Lespedezatomentosa* Siebold [5, 9]

*Lotuskrylovii* Schischk. & Serg. [7, 9, 10, 12, 14–16]

*Medicagofalcata* L. [2–12, 14]

*Medicagolupulina* L. [2–5, 7–11, 13, 14]

*Medicagoplatycarpa* (L.) Trautv. [1, 2, 3, 4, 7, 9]

*Medicagoruthenica* Trautv. [1–5, 8–11, 13, 14]

*Melilotusdentatus* (Waldst. & Kit.) Pers. [2–5, 7–12, 14]

*Melilotusofficinalis* (L.) Lam. [2, 3, 4]

*Melilotussuaveolens* Ledeb. [≡ *Trigonellasuaveolens* (Ledeb.) Coulot & Rabaute] [1, 3–14]

*Melilotuswolgicus* Poir. [9, 12]

Onobrychisarenaria(Kit.)DC.subsparenaria [2, 3, 4, 8]

Onobrychisarenariasubsp.sibirica (Turcz.) P.W.Ball [≡ *Onobrychissibirica* (Sirj.) Turcz.] [2, 3, 4, 13]

SE*Oxytropisacanthacea* Jurtzev [6, 7]

*Oxytropisaciphylla* Ledeb. [3, 6, 7, 10–16]

SE*Oxytropisalpestris* Schischk. [7]

*Oxytropisalpicola* Turcz. [2]

*Oxytropisalpina* Bunge [1, 2, 3, 6, 7, 13]

*Oxytropisaltaica* (Pall.) Pers. [6, 7]

*Oxytropisambigua* (Pall.) DC. [1, 2, 3, 4, 7, 13]

*Oxytropisampullata* (Pall.) Pers. [2, 3, 7–9, 12, 13]

SE*Oxytropisbaicalia* (Pall.) Pers. [1, 3, 4]

SE*Oxytropisbicolor* Bunge [9]

*Oxytropisbrachycarpa* Vassilcz. [7]

E *Oxytropisbungei* Kom. [3, 6, 7, 8, 10–14]

*Oxytropiscaerulea* DC. [1, 2, 4, 5, 9]

*Oxytropiscaespitosa* Pers. [1, 2, 3, 4, 5, 8]

*Oxytropiscampanulata* Vassilcz. [1, 3]

*Oxytropischionophylla* Schrenk [3, 6, 7, 13]

*Oxytropisdeflexa* (Pall.) DC. [1–4, 6, 7, 10, 13]

*Oxytropisdiantha* Bunge [= *Oxytropischangaica* B.Fedtsch. & Basil.] [1, 3]

SE*Oxytropisdubia* Turcz. [2]

SE*Oxytropiseriocarpa* Bunge [6, 7]

*Oxytropisfalcata* Bunge [6, 7]

E *Oxytropisfragilifolia* N.Ulziykh. [7, 13]

SE*Oxytropisgebleri* Fisch. [1, 3, 6, 7, 13]

*Oxytropisglabra* DC. [1–4, 6–16]

*Oxytropisglandulosa* Turcz. [1, 3]

*Oxytropisglareosa* Vassilcz. [3, 10]

*Oxytropisgorbunovii* Boriss. [3, 6, 7]

*Oxytropisgrandiflora* DC. [2, 4, 5, 8, 9]

*Oxytropishailarensis* Kitag. [5, 9]

SE*Oxytropisheterophylla* Bunge [6, 7, 10, 13, 14]

*Oxytropishirta* Bunge [5]

SE*Oxytropisintermedia* Bunge [3, 6, 7, 10]

E *Oxytropisjunatovii* Sanchir [13]

SE*Oxytropisjurtzevii* Malyschev [1]

E *Oxytropisklementzii* N.Ulziykh. [2, 3, 4, 8]

SE*Oxytropiskomarovii* Vassilcz. [5, 9]

SE*Oxytropiskossinskyi* B.Fedtsch. & Basil. [3, 4, 8, 11, 13]

*Oxytropiskrylovii* Schipcz. [7]

SE*Oxytropiskusnetzovii* Kryl. & Steinb. [1, 7]

*Oxytropisladyginii* Krylov [7]

*Oxytropislanata* DC. [1, 3, 4, 8, 9]

SE*Oxytropislanuginosa* Kom. [3, 10]

*Oxytropislapponica* Gaudin [3, 6, 7]

*Oxytropislasiopoda* Bunge [3, 4, 8, 9, 13]

SE*Oxytropislatibracteata* Jurtzev [3]

E *Oxytropislavrenkoi* N.Ulziykh. [12]

*Oxytropisleptophylla* DC. [1, 3–5, 8, 9, 12]

SE*Oxytropisleucotricha* Turcz. [1, 2, 3, 8]

*Oxytropislongirostra* DC. [1, 2, 3]

*Oxytropismacrosema* Bunge [6, 7]

SE*Oxytropismartjanovii* Krylov [3, 6, 7, 10]

E *Oxytropismicrantha* Bunge [3, 6, 7, 10, 11]

*Oxytropismicrophylla* (Pall.) DC. [3, 6, 7, 10, 12]

SE*Oxytropismixotriche* Bunge [2, 3, 4, 8]

SE*Oxytropismongolica* Kom. [6, 10]

SE*Oxytropismonophylla* Grubov [12, 13]

*Oxytropismuricata* (Pall.) DC. [1, 3, 4, 9, 13]

*Oxytropismyriophylla* (Pall.) DC. [1–5, 8, 9]

SE*Oxytropisnitens* Turcz. [1, 2, 3, 4, 8, 9]

SE*Oxytropisochrantha* Turcz. [9]

*Oxytropisoligantha* Bunge [3, 6, 7, 10, 13]

*Oxytropisoxyphylla* (Pall.) DC. [1–5, 8, 9, 12]

*Oxytropispauciflora* Bunge [1, 6, 7, 13]

E *Oxytropispavlovii* B.Fedtsch. & Basil. [3, 8, 11, 12, 13]

SE*Oxytropisphysocarpa* Ledeb. [7]

E *Oxytropispotaninii* Bunge [7, 10]

SE*Oxytropisprostrata* (Pall.) DC. [4, 8, 9]

SE*Oxytropispseudoglandulosa* Gontsch. [1–4, 8, 9, 12, 13]

*Oxytropispuberula* Boriss. [7, 13, 14]

*Oxytropispumila* Fisch. [3, 6, 7, 8, 10, 11, 13]

*Oxytropisracemosa* Turcz. [= *Oxytropisgracillima* Bunge] [3, 4, 6, 8–12]

*Oxytropisrecognita* Bunge [6, 7]

SE*Oxytropisreverdattoi* Jurtzev [2, 3, 4]

SE*Oxytropisrhizantha* Palib. [6, 7, 10]

*Oxytropisrhynchophysa* Schrenk [6, 7]

SE*Oxytropissacciformis* H.C.Fu [12]

SE*Oxytropissajanensis* Jurtzev [1, 3]

*Oxytropissaposhnikovii* Krylov [7, 10]

SE*Oxytropisselengensis* Bunge [2, 3, 4, 8, 9]

SE*Oxytropissetosa* (Pall.) DC. [3, 6]

*Oxytropissongorica* (Pall.) DC. [7]

*Oxytropissordida* (Willd.) Pers. [1]

*Oxytropissquammulosa* DC. [2–4, 6–10, 12, 13]

SE*Oxytropisstenophylla* Bunge [3, 13]

*Oxytropisstrobilacea* Bunge [1–4, 6, 7, 13]

SE*Oxytropisstukovii* Palib. [3, 9]

*Oxytropissulphurea* Ledeb. [7]

E *Oxytropissutaica* N.Ulziykh. [3, 7]

E *Oxytropistenuis* Palib. [6, 7]

*Oxytropisteres* DC. [7]

*Oxytropistragacanthoides* Fisch. [1, 3, 6–8, 10, 11, 13–15]

*Oxytropistrichophysa* Bunge [3, 6, 7, 10, 11, 13, 14]

SE*Oxytropistschujae* Bunge [1, 7]

SE*Oxytropisturczaninovii* Jurtzev [1, 3, 4]

E *Oxytropisulzijchutagii* Sanchir [7]

SE*Oxytropisvarlakovii* Serg. [4, 9]

E *Oxytropisviridiflava* Kom. [1–4, 7–9, 11, 13]

*Sophoraalopecuroides* L. [12–16]

*Sophoraflavescens* Aiton [5, 9]

*Sphaerophysasalsula* (Pall.) DC. [5, 7, 9–16]

*Thermopsisalpina* Ledeb. [1, 2]

SE*Thermopsisdahurica* Czefr. [2, 4, 5, 9, 12]

*Thermopsislanceolata* R.Br. [= Thermopsislanceolatavar.glabra (Czefr.) Yakovlev] [1–5, 8, 9, 11, 13]

E *Thermopsislongicarpa* N.Ulziykh. [6, 10]

*Thermopsismongolica* Czefr. [≡ Thermopsislanceolatavar.mongolica (Czefr.) Q.R.Wang & X.Y.Zhu] [6, 7, 10–14, 16]

SE*Thermopsisprzewalskii* Czefr. [9, 13]

*Trifoliumeximium* Steph. [1–4, 6–11, 13]

*Trifoliumlupinaster* L. [1–9]

*Trifoliumpratense* L. [1, 4]

*Trifoliumrepens* L. [2, 7]

*Trigonellaarcuata* C.A.Mey. [7, 14]

*Trigonellacancellata* Desf. [7, 14]

*Viciaamoena* Fisch. [= Viciaamoenasubsp.sericea (Kitag.) Kamelin & Gubanov] [1–5, 7, 8, 9]

*Viciaamurensis* Oett. [4, 5, 9]

*Viciacostata* Ledeb. [2–4, 6–14, 16]

*Viciacracca* L. [1–10, 14]

*Viciageminiflora* Trautv. [3, 4, 5]

*Viciajaponica* A.Gray [2, 5]

*Viciamacrantha* Jurtzev [= Viciamacranthasubsp.olchonensis Peschkova] [1, 2, 3]

*Viciamegalotropis* Ledeb.. [1–5, 8, 9]

*Viciamulticaulis* Ledeb. [= *Vicianervata* Sipliv.] [1–6, 8, 13]

SE*Viciaolchonensis* (Peschkova) O.D.Nikif. [≡ Viciamacranthasubsp.olchonensis Peschkova] [1, 4]

*Viciapseudorobus* Fisch. & C.A.Mey. [5, 9]

*Viciaramuliflora* (Maxim.) Ohwi [= *Viciabaicalensis* (Turcz.) B.Fedtsch.] [2, 3, 4, 5]

*Viciasemenovii* B.Fedtsch. [3, 13]

*Viciatenuifolia* Roth [3, 6, 7]

SE*Viciatsydenii* Malyshev [4]

*Viciaunijuga* A.Braun [1–8]

*Viciavenosa* Maxim. [1, 2, 3, 4, 5, 8]

**53. Frankeniaceae** Desv. (1 genus and 2 species)

*Frankeniapulverulenta* L. [10]

SE*Frankeniatuvinica* Lomon. [10]

**54. Gentianaceae** Juss. (8 genera and 32 taxa)

Centauriumpulchellumsubsp.meyeri (Bunge) Tzvelev [7, 10, 13]

Centauriumpulchellum(Sw.)Hayeksubsp.pulchellum [10, 11, 15]

*Comastomafalcatum* (Turcz.) Toyokuni [1, 6, 7, 13]

*Comastomamalyschevii* (Zuev) Zuev [≡ *Gentianellamalyschevii* Zuev] [1, 3, 7]

*Comastomapulmonarium* (Turcz.) Toyokuni [1, 2, 3, 6]

*Comastomatenellum* (Rottb.) Toyok. [≡ *Gentianatenella* Rottb.] [1–3, 6, 7, 13, 14]

*Gentianaalgida* Pall. [1–3, 6, 7, 13]

GentianaaquaticaL.subsp.aquatica [1, 2, 3, 6, 7, 8]

Gentianaaquaticavar.pseudoaquatica (Kusn.) S.Agrawal [≡ *Gentianapseudoaquatica* Kusnezow] [1–4, 6–9, 13]

*Gentianadahurica* Fisch. [≡ *Dasystephanadahurica* (Fisch.) Zuev] [4, 5, 8, 9]

*Gentianadecumbens* L.f. [≡ *Dasystephanadecumbens* (L.f.) Zuev] [1–11, 13, 14]

*Gentianagrandiflora* Laxm. [1, 2, 3, 7]

*Gentianaleucomelaena* Maxim. [≡ *Ciminalisleucomelaena* (Maxim.) Zuev] [1–4, 7, 8, 10, 11, 13, 14]

*Gentianamacrophylla* Pall. [1–7, 9, 13, 14]

*Gentianaprostrata* Haenke [1–4, 6–8, 10, 11, 13, 14]

*Gentianakarelinii* Griseb. [≡ Gentianaprostratavar.karelinii (Griseb.) Kusn.] [7]

*Gentianariparia* Kar. & Kir. [7, 14]

*Gentianasquarrosa* Ledeb. [≡ *Ciminalissquarrosa* (Ledeb.) Zuev] [1–11]

*Gentianatriflora* Pall. [2, 4, 5]

*Gentianauniflora* Georgi [1, 3, 6, 7]

GentianellaamarellaL.subsp.acuta (Michx.) J.M.Gillett [≡ *Gentianaacuta* Michx.] [1–4, 6–9, 13]

*Gentianellaatrata* (Bunge) Holub [5]

*Gentianellaaurea* (L.) Harry Sm. [2, 3, 6, 7, 13]

*Gentianellaturkestanorum* (Gand.) Holub [7, 14]

*Gentianopsisbarbata* (Froel.) Ma [1–11, 13, 14]

*Haleniacorniculata* (L.) Cornaz [≡ *Swertiacorniculata* L.] [1–5, 8, 13]

*Lomatogoniumcarinthiacum* (Wulfen) Rchb. [≡ *Swertiacarinthiaca* Wulfen] [1–4, 6–8, 11, 13]

*Lomatogoniumrotatum* Fr. [1–8, 10, 13, 14]

*Swertiabanzragczii* Sanchir [6, 7]

*Swertiadichotoma* L. [≡ *Anagallidiumdichotomum* (L.) Griseb.] [1, 2, 3, 4, 8, 9]

*Swertiamarginata* Schrenk [= *Swertiakomarovii* Pissjauk.] [1, 7]

*Swertiaobtusa* Ledeb. [2, 6, 7]

**55. Geraniaceae** Juss. (2 genera and 19 taxa)

*Erodiumcicutarium* (L.) L’Hér. [2, 4, 12]

*Erodiumstephanianum* Willd. [2–5, 7–16]

*Erodiumtibetanum* Edgew. & Hook.f. [4, 6, 7, 8, 10–16]

*Geraniumaffine* Ledeb. [6, 7, 14]

*Geraniumalbiflorum* Ledeb. [2, 3, 6, 7]

*Geraniumamurense* Tsyren. [3, 4, 9]

*Geraniumcollinum* Stephan [7, 10, 14, 15, 16]

*Geraniumdahuricum* DC. [1, 5, 6, 9, 10, 12]

*Geraniumkrylovii* Tzvelev [2, 3, 4]

*Geraniumlaetum* Ledeb. [3, 7, 14]

*Geraniumpamiricum* Ikonn. [14]

*Geraniumplatyanthum* Duthie [2, 3, 4, 5, 9, 12]

*Geraniumpratense* L. [1–4, 6–9, 12, 13]

*Geraniumpseudosibiricum* J.Mayer [1–8, 10, 14]

*Geraniumsaxatile* Kar. & Kir. [7, 14]

*Geraniumsibiricum* L. [1–5, 7–10, 12–14, 16]

*Geraniumtransbaicalicum* Serg. [1,7, 9]

Geraniumtransbaicalicumsubsp.turczaninovii (Serg.) Peschkova [3, 4]

*Geraniumwlassovianum* Fisch. [1, 2, 3, 4, 5, 9]

**56. Grossulariaceae** DC. (1 genus and 12 taxa)

*Ribesaciculare* Sm. [≡ *Grossulariaacicularis* (Sm.) Spach] [2–4, 6–8, 10, 13, 14]

*Ribesdiacanthum* Pall. [=*Ribesdiacanthum f. weichangense* J.X.Huang & J.Z.Wang] [1–5, 8, 9]

*Ribesfragrans* Pall. [1, 2]

*Ribesgraveolens* Bunge [1, 7]

*Ribesheterotrichum* C.A.Mey. [6, 7, 10, 14, 15]

*Ribesmeyeri* Maxim. [7, 14]

*Ribesnigrum* L. [1–7, 10, 13]

*Ribespetraeum* Wulfen [= *Ribesaltissimum* Turcz.] [1–4, 6, 7, 14]

*Ribesprocumbens* Pall. [1, 3, 7]

*Ribespulchellum* Turcz. [1–5, 8, 9, 12, 14]

*Ribesrubrum* L. [1–7, 9, 13]

*Ribesspicatum* E.Robson [1–3, 6, 7, 9, 10]

**57. Haloragaceae** R.Br. (1 genus and 3 species)

*Myriophyllumsibiricum* Kom. [5]

*Myriophyllumspicatum* L. [1–11, 14]

*Myriophyllumverticillatum* L. [1–10, 14]

**58. Hydrocharitaceae** Juss. (2 genera and 5 species)

*Hydrillaverticillata* (L.f.) Royle [≡ *Serpiculaverticillata* L.f.] [10]

*Najasflexilis* (Willd.) Rostk. & W.L.E.Schmidt [≡ *Cauliniaflexilis* Willd.] [10]

*Najasmarina* L. [10, 11]

*Najasminor* All. [10]

*Najastenuissima* (A.Braun) Magnus [≡ *Cauliniatenuissima* (A.Braun) Tzvelev] [10]

**59. Hypericaceae** Juss. (1 genus and 4 taxa)

HypericumascyronL.subsp.ascyron [2, 3, 4, 5]

Hypericumascyronsubsp.gebleri (Ledeb.) N.Robson [≡ *Hypericumgebleri* Ledeb.] [2]

*Hypericumattenuatum* Choisy [2, 3, 4, 5, 9]

*Hypericumperforatum* L. [3, 6]

**60. Iridaceae** Juss. (1 genus and 21 taxa)

SE*Irisbungei* Maxim. [≡ *Cryptobasisbungei* (Maxim.) M.B.Crespo] [3, 5, 8, 9, 11–13, 16]

*Irisdichotoma* Pall. [2, 4, 5, 8, 9]

*Irisglaucescens* Bunge [6]

*Irishalophila* Pall. [≡ *Chamaeirishalophila* (Pall.) M.B.Crespo] [6, 14]

*Irishumilis* Georgi [= *Irisflavissima* Pall.] [1–5, 8, 9, 12, 13]

SE*Irisivanovae* Doronkin [2, 3]

SE*Iriskamelinii* Alexeeva [1, 7]

*Irislactea* Pall. [≡ *Eremirislactea* (Pall.) Rodion.] [1–13, 15, 16]

*Irisloczyi* Kanitz [7, 10]

*Irisludwigii* Maxim. [≡ *Xyridionludwigii* (Maxim.) Rodion.] [7]

*Irispotaninii* Maxim. [1–4, 6–13]

*Irispsammocola* Y.T.Zhao [10]

SE*Irispseudothoroldii* Galanin [4]

Irisruthenicasubsp.brevituba (Maxim.) Doronkin [≡ Irisruthenicavar.brevituba Maxim.] [1, 2]

IrisruthenicaKer Gawl.subsp.ruthenica [1, 2, 3, 4, 5]

E *Irisschmakovii* Alexeeva [1]

*Irissibirica* L. [= *Irissanguinea* Donn] [2, 4, 5, 9]

*Iristenuifolia* Pall. [7–15]

*Iristigridia* Bunge [1, 2, 3, 4, 8]

*Irisuniflora* Pall. [≡ *Jonirisuniflora* (Pall.) M.B.Crespo] [4, 5]

*Irisventricosa* Pall. [≡ *Cryptobasisventricosa* (Pall.) M.B.Crespo] [5, 9]

**61. Juncaceae** Juss. (2 genera and 32 taxa)

Juncusalpinoarticulatussubsp.fischerianus (V.I.Krecz.) Hämet-Ahti [1–10, 14]

SEJuncusarcticussubsp.grubovii (Novikov) Novikov [≡ *Juncusgrubovii* Novikov] [1, 2, 3]

JuncusarticulatusL.subsp.articulatus [4, 9, 14]

Juncusarticulatussubsp.limosus (Vorosch.) Vorosch. [≡ *Juncuslimosus* Vorosch.] [3, 4, 5, 9]

*Juncusbiglumis* L. [1, 6, 7]

*Juncusbufonius* L. [1–16]

Juncuscastaneussubsp.leucochlamys (V.I.Krecz.) Hultén [≡ *Juncusleucochlamys* V.I.Krecz.] [1–4, 6, 7, 9]

Juncuscastaneussubsp.triceps (Rostk.) Novikov [1–3, 6, 7, 13]

*Juncuscompressus* Jacq. [1, 3–5, 8–10, 13, 14]

*Juncusfiliformis* L. [7]

*Juncusgerardi* Loisel [2–4, 6–10, 13–15]

*Juncusgracillimus* (Buchenau) V.I.Krecz. & Gontsch. [2, 3, 5, 9]

*Juncushybridus* Brot. [= Juncusbufoniussubsp.ambiguus (Guss.) Schinz & Thell.] [3, 4, 6, 7, 9–11, 13–15]

*Juncusorchonicus* Novikov [2–5, 8, 9, 10]

Juncuspersicussubsp.libanoticus (J.Thiébaut) Novikov & Snogerup [= *Juncuslibanoticus* J.Thiébaut] [3, 4, 6, 7]

*Juncusranarius* Songeon & E.P.Perrier [= Juncusbufoniussubsp.nastanthus (V.I.Krecz. & Gontsch.) Soó] [10, 14]

*Juncussalsuginosus* Turcz. [1–4, 6–8, 10–13, 15]

*Juncussoranthus* Schrenk [3, 7]

*Juncustriglumis* L. [1–4, 6, 7, 10, 13, 14]

*Juncusturkestanicus* V.I.Krecz. & Gontsch. [≡ Juncusbufoniussubsp.turkestanicus (V.I.Krecz. & Gontsch.) Novikov] [3–5, 7, 9, 10, 14]

*Juncusvirens* Buchenau [≡ Juncuspapillosusvar.virens (Buchenau) Vorosch.] [4]

*Luzulaconfusa* Lindeb. [1, 2, 6, 7]

*Luzulamultiflora* (Ehrh.) Lej. [3]

Luzulamultiflorasubsp.frigida (Buchenau) V.I.Krecz. [7]

Luzulamultiflorasubsp.sibirica V.I.Krecz. [≡ *Luzulasibirica* (V.I.Krecz.) V.I.Krecz.] [1– 4, 6, 7]

*Luzulanivalis* (Laest.) Spreng. [≡ Luzulacampestrisvar.nivalis Laest.] [1]

*Luzulapallescens* Sw. [1–5, 7, 9]

*Luzulaparviflora* Desv. [1, 2, 3, 6, 7]

*Luzulapilosa* (L.) Willd. [≡ *Juncuspilosus* L.] [2]

Luzularufescensvar.macrocarpa Buchenau [= *Luzulachangaica* Novikov] [3]

Luzulaspicatasubsp.mongolica Novikov [1–3, 6, 7, 13]

LuzularufescensFisch.var.rufescens [1, 2, 3, 4]

**62. Juncaginaceae** Juss. (1 genus and 2 species)

*Triglochinmaritima* L. [1–16]

*Triglochinpalustris* L. [1–9, 11–16]

**63. Lamiaceae** Martinov (22 genera and 103 taxa)

Note: The herbarium records of *Phlomisoreophila* in Mongolia was identified as *Phlmoideschinghoensis* by Lazkov (2011).

*Amethysteacaerulea* L. [2–11, 13]

*Caryopterismongholica* Bunge [2–4, 7–9, 11–13, 15, 16]

*Dracocephalumargunense* Fisch. [5]

*Dracocephalumdiscolor* Bunge [3, 7, 10]

*Dracocephalumfoetidum* Bunge [1–4, 6–13]

*Dracocephalumfragile* Turcz. [1, 3, 6, 7]

*Dracocephalumfruticulosum* Stephan [3, 4, 6–8, 10–13, 16]

*Dracocephalumgrandiflorum* L. [1–3, 6, 7, 13]

Dracocephalumheterophyllumsubsp.heterophyllum Benth. [3]

Dracocephalumheterophyllumsubsp.ovalifolium A.L.Budantzev [≡ *Dracocephalumovalifolium* (A.L.Budantzev) Doronkin] [3]

*Dracocephalumimberbe* Bunge [1, 6, 7]

*Dracocephalumintegrifolium* Bunge [≡ *Ruyschianaintegrifolia* (Bunge) House] [6, 7]

*Dracocephalumjunatovii* A.L.Budantzev [4, 9]

*Dracocephalummoldavicum* C.Morren [12, 13, 15]

*Dracocephalumnodulosum* Rupr. [14]

*Dracocephalumnutans* L. [1, 2, 3, 4, 7]

*Dracocephalumolchonense* Peschkova [4]

DracocephalumoriganoidesSteph.subsp.origanoides [1, 3, 4, 6–9, 13, 14]

Dracocephalumoriganoidessubsp.bungeanum (Schischk. & Serg. A.L.Budantzev [≡ *Dracocephalumbungeanum* Schischk. & Serg.] [1, 6, 7, 13]

*Dracocephalumpaulsenii* Briq. [14]

*Dracocephalumperegrinum* L. [6, 7]

*Dracocephalumpinnatum* L. [6]

*Dracocephalumruyschiana* L. [2, 3, 4, 5, 6, 8]

*Elsholtziaciliata* (Thunb.) Hyl. [2, 4]

*Elsholtziadensa* Benth. [4, 13]

*Galeopsisbifida* Boenn. [≡ Galeopsistetrahitvar.bifida (Boenn.) Lej. & Courtois] [2, 3, 4, 9]

*Hyssopusambiguus* (Trautv.) Iljin [= Hyssopusofficinalisvar.ambiguus Trautv.] [7]

*Hyssopuscuspidatus* Boriss. [7, 14]

*Lagochilusbungei* Benth. [7, 14]

*Lagochilusdiacanthophyllus* Benth. [6, 7, 14]

*Lagochilusilicifolius* Bunge [3, 7, 8, 10–16]

E *Lagopsisdarwiniana* Pjak [7]

*Lagopsiseriostachya* (Benth.) Ikonn.-Gal. [1, 7, 10, 14]

*Lagopsisflava* Kar. & Kir. [7]

*Lagopsismarrubiastrum* (Steph.) Ikonn.-Gal. [3, 6, 7, 13, 14]

*Lagopsissupina* (Steph.) Ikonn.-Gal. [2, 3, 4, 9]

*Lamiumalbum* L. [1, 2, 4, 5, 7, 9]

*Leonurusdeminutus* V.I.Krecz. [≡ Leonurusglaucescensvar.deminutus (V.I.Krecz.) Karav.] [1–4, 7, 8, 9, 13]

*Leonurusglaucescens* Bunge [6, 7, 8, 9]

*Leonurusmongolicus* V.I.Krecz. & Kuprian. [2–4, 6–9]

*Leonuruspseudopanzerioides* Krestovsk. [= Leonuruscardiacasubsp.turkestanicus (V.I.Krecz. & Kuprian.) Rech.f.] [7, 14]

*Leonurussibiricus* L. [1–5, 8, 9, 12]

*Leonurusturkestanicus* V.I.Krecz. & Kuprian. [7]

*Lophanthuschinensis* Benth. [1–4, 6–10, 12, 13]

*Lophanthuskrylovii* Lipsky [7]

*Lycopuslucidus* Turcz. [9]

*Menthaaquatica* L. [4]

*Menthaarvensis* L. [2–10, 14]

*Menthacanadensis* L. [2]

*Nepetaannua* Pall. [≡ *Schizonepetaannua* (Pall.) Schischk.] [3, 6–16]

*Nepetadensiflora* Kar. & Kir. [7, 14]

*Nepetamicrantha* Bunge [7, 14]

*Nepetamultifida* L. [1–5, 7–9, 13]

*Nepetanuda* L. [6]

*Nepetapungens* Benth. [14]

*Nepetasibirica* L. [2, 3, 6, 7, 10, 13, 14]

*Origanumvulgare* L. [1, 6, 9]

*Panzerinacanescens* (Bunge) Soják [6, 7, 10, 13]

*Panzerinalanata* (L.) Soják [≡ *Ballotalanata* Willd.] [2, 3, 4, 6–14, 16]

*Phlomoidesagraria* (Bunge) Adylov [≡ *Phlomisagraria* Bunge] [6, 7]

*Phlomoidesalpina* (Pall.) Adylov [≡ *Phlomisalpina* Pall.] [7]

SE*Phlomoideschinghoensis* (C.Y.Wu) Kamelin & Makhm. [≡ *Phlomischinghoensis* C.Y.Wu] [7, 10, 14]

*Phlomoidesmolucelloides* (Bunge) Salmaki [≡ *Eremostachysmolucelloides* Bunge] [6, 14]

*Phlomoidesmongolica* (Turcz.) Kamelin & A.L.Budantzev [≡ *Phlomismongolica* Turcz.] [5, 9]

*Phlomoidespratensis* (Kar. & Kir.) Adylov [≡ *Phlomispratensis* Kar. & Kir.] [6]

*Phlomoidestuberosa* Moench [≡ *Phlomistuberosa* L.] [2–9]

*Phlomoidestuvinica* (A.Schroet.) Kamelin [≡ *Phlomistuvinica* A.Schroet.] [6, 7, 8]

*Salviaabrotanoides* (Kar.) Sytsma [≡ *Perovskiaabrotanoides* Kar.] [6]

*Salviadeserta* Schangin [6]

*Scutellariaaltaica* Ledeb. [7]

*Scutellariabaicalensis* Georgi [1–5, 8, 9]

*Scutellariadependens* Maxim. [2, 4]

*Scutellariagalericulata* L. [1–6, 9, 10, 14]

ScutellariagrandifloraSimssubsp.grandiflora [2–4, 6, 7, 10, 13, 14]

E Scutellariagrandiflorasubsp.gymnosperma Kamelin & Gubanov [7, 13]

*Scutellariakrasevii* Kom. & I.Schischk. [3]

*Scutellariapaulsenii* Briq. [7]

Scutellariaregelianavar.ikonnikovii (Juz.) C.Y.Wu & H.W.Li [2, 4]

*Scutellariascordiifolia* Fisch. [1–9]

*Scutellariasieversii* Bunge [6, 7]

*Scutellariasupina* L. [≡ Scutellariaalpinasubsp.supina (L.) I.Richardson] [7, 14]

*Scutellariatuvensis* Juz. [≡ Scutellariagrandiflorasubsp.tuvensis (Juz.) Kamelin & Gubanov] [10]

*Scutellariaviscidula* Bunge [9]

Stachysasperasubsp.baicalensis (Fisch.) Krestovsk. [≡ *Stachysbaicalensis* Fisch.] [2, 3, 4, 5]

*Stachyspalustris* L. [2–6, 9, 10]

*Thymusaltaicus* Klokov & Des.-Shost. [3, 6, 7, 10]

*Thymusbaicalensis* Serg. [1, 2, 3, 4, 10]

*Thymusbituminosus* Klokov [1]

*Thymusdahuricus* Serg. [2, 4, 5, 8, 9]

E *Thymusgobi-altaicus* (N.Ulziykh.) Kamelin & A.L.Budantzev [13]

*Thymusgobicus* Tscherneva [2, 3, 4, 7–13]

*Thymuskomarovii* Serg. [9]

*Thymusmichaelis* Kamelin & A.L.Budantzev [2, 4, 8, 9]

*Thymusminussinensis* Serg. [10]

*Thymusmongolicus* (Ronniger) Ronniger [3, 7, 9, 13]

*Thymusnarymensis* Serg. [7]

*Thymuspavlovii* Serg. [1, 3]

*Thymusroseus* Schipcz. [7]

*Thymussibiricus* Klokov & Des.-Shost. [4]

*Thymusturczaninovii* Serg. [9]

ZiziphoraclinopodioidesLam.subsp.clinopodioides [7, 14]

Ziziphoraclinopodioidessubsp.bungeana (Juz.) Rech.f. [≡ *Ziziphorabungeana* Juz.] [6, 7, 13]

*Ziziphorapamiroalaica* Juz. [7, 14]

**64. Lentibulariaceae** Rich. (2 genera and 7 species)

*Pinguiculaalpina* L. [1]

*Pinguiculavulgaris* L. [1]

*Utriculariaaustralis* R.Br. [10, 14]

*Utriculariaintermedia* Hayne [≡ *Lentibulariaintermedia* (Hayne) Nieuwl. & Lunell] [1, 2, 3, 6]

Utricularia×japonica Makino [10]

*Utriculariaminor* L. [3, 9, 10, 14]

*Utriculariavulgaris* L. [≡ *Lentibulariavulgaris* (L.) Moench] [1–11, 14, 15]

**65. Liliaceae** Juss. (5 genera and 15 taxa)

*Erythroniumsibiricum* (Fisch. & C.A.Mey.) Krylov [7]

SE*Fritillariadagana* Turcz. [1, 2, 3]

*Gageabrevistolonifera* Levichev [7]

*Gageafiliformis* Merckl. [7]

*Gageagranulosa* Turcz. [7]

*Gageahiensis* Pasch. [= *Gageaterraccianoana* Pasch.] [1, 2]

SE*Gageakuraiensis* Levichev [7]

*Gageafragifera* (Vill.) Ehr.Bayer & G.López [= *Gagealiotardii* (Sternb.) Schult. & Schult. f.] [7]

*Gageapauciflora* Turcz. [1–5, 7, 9, 14]

*Gageaserotina* (L.) Ker Gawl. [≡ *Lloydiaserotina* (L.) Salisb.] [1–3, 6, 7, 13]

Liliumconcolorvar.partheneion (Siebold & de Vriese) Baker [= *Liliumbuschianum* G.Lodd.] [5]

*Liliummartagon* L. [1–7]

*Liliumpensylvanicum* Ker Gawl. [= *Liliumdauricum* Ker Gawl.] [2, 4, 5]

*Liliumpumilum* Redouté [= *Liliumpotaninii* Vrishcz] [1–5, 8, 9, 12]

*Tulipauniflora* (L.) Besser [3, 5, 7–10, 14]

**66. Linaceae** DC. (1 genus and 5 species)

*Linumaltaicum* Ledeb. [1, 6, 7, 8, 9]

*Linumbaicalense* Juz. [1–5, 7–9, 13]

*Linumpallescens* Bunge [2–5, 7–10, 13, 14]

*Linumperenne* L. [7, 14]

*Linumviolascens* Bunge [7]

**67. Lythraceae** J.St.-Hil. (1 genus and 3 species)

*Lythrumsalicaria* L. [4]

*Lythrumvirgatum* L. [6, 14]

*Lythrumborysthenicum* (Schrank) Litv. [≡ *Peplisborysthenica* Schrank] [10]

**68. Malvaceae** Juss. (2 genera and 5 species)

*Abutilontheophrasti* Medik. [15]

*Malvaneglecta* Wallr.. [4, 7, 10, 11, 14]

*Malvapusilla* Sm. [13, 14, 15]

*Malvasylvestris* L. [13, 15]

*Malvaverticillata* L. [1–4, 7, 8, 10, 16]

**69. Mazaceae** Reveal (3 genera and 3 species)

Note: Mazaceae was separated from Phrymaceae according to APG IV (2016).

*Dodartiaorientalis* L. [6, 7, 14]

*Lanceatibetica* Hook.f. & Thomson [1, 3]

*Mazusstachydifolius* Maxim. [5]

**70. Melanthiaceae** Batsch (3 genera and 5 species)

*Anticleasibirica***(L.)** Kunth [≡ *Zigadenussibiricus* (L.) A.Gray] [1, 3]

*Parisquadrifolia* L. [2, 3, 4, 5]

*Parisverticillata* M.Bieb. [2, 4, 5]

*Veratrumlobelianum* Bernh. [1, 3, 4, 5, 7, 9]

*Veratrumnigrum* L. [3, 4, 5, 9]

**71. Menispermaceae** Juss. (1 genus and 1 species)

*Menispermumdauricum* DC. [2, 3, 4, 5]

**72. Menyanthaceae** Dumort. (2 genera and 2 species)

*Nymphoidespeltata* (S.G.Gmel.) Kuntze [1, 3, 8–10, 14]

*Menyanthestrifoliata* L. [1, 2, 3, 4]

**73. Molluginaceae** Bartl. (1 genus and 1 species)

*Hyperteliscerviana* (L.) Thulin [≡ *Mollugocerviana* (L.) Ser.] [12, 14, 15]

**74. Montiaceae** Raf. (1 genus and 1 species)

*Claytoniajoanneana* Roem. & Schult. [1, 2, 3, 4, 6, 7]

**75. Nitrariaceae** Lindl. (2 genera and 5 species)

*Nitrariaroborowskii* Kom. [≡ Nitrariaschoberivar.roborowskii (Kom.) Grubov] [7, 10, 13, 14, 15]

*Nitrariasibirica* Poir. [4, 6–16]

*Nitrariasphaerocarpa* Maxim. [13, 15, 16]

*Peganumharmala* L. [= *Peganummultisectum* (Maxim.) Bobrov] [7, 10, 13, 14, 15]

*Peganumnigellastrum* Bunge [3, 4, 8–13, 16]

**76. Nymphaeaceae** Salisb. (2 genera and 2 species)

Note: Recently, this family was revised based on field observations and extensive herbarium specimens in Mongolia (Baasanmunkh et al. 2022b). *Nymphaeatetragona* Georgi is not recorded in Mongolia, according to Baasanmunkh et al (2022b).

*Nupharpumila* (Timm) DC. [1, 3, 10, 11]

*Nymphaeacandida* J.Presl. [1, 3, 7, 10, 11]

**77. Onagraceae** Juss. (2 genera and 12 taxa)

CircaeaalpinaL.subsp.alpina [2, 3, 4, 5]

Circaeaalpinasubsp.caulescens (Kom.) Tatew. [3]

*Epilobiumanagallidifolium* Lam. [= *Epilobiumalpinum* L.] [7]

*Epilobiumangustifolium* L. [≡ *Chamaenerionangustifolium* (L.) Schur] [1–9, 14]

*Epilobiumciliatum* Raf. [2]

*Epilobiumdavuricum* Fisch. [2, 3, 4, 11]

*Epilobiumfastigiato-ramosum* Nakai [= *Epilobiumbaicalense* Popov] [3, 4, 9]

*Epilobiumhirsutum* L. [3, 6]

*Epilobiumlatifolium* L. [= *Chamaenerionlatifolium* (L.) Sweet] [1–3, 6, 7, 13, 14]

*Epilobiumminutiflorum* Hausskn. [7, 10, 15]

*Epilobiumnervosum* Boiss. & Buhse [7]

*Epilobiumpalustre* L. [1–10, 13–15]

**78. Orchidaceae** Juss. (14 genera and 26 taxa)

Note: Orchids of Mongolia were recently revised by Baasanmunkh et al. (2021b).

*Calypso bulbosa* (L.) Oakes [= *Cypripediumbulbosum* L.] [2, 4]

*Corallorhizatrifida* Châtel. [1, 2, 4]

*Cypripediumcalceolus* L.. [1, 2, 4]

*Cypripediumguttatum* Sw. [1, 2, 3, 4, 5]

*Cypripediummacranthos* Sw. [1, 2, 4, 5]

*Cypripedium***x***ventricosum* Sw. [2]

*Dactylorhizafuchsii* (Druce) Soó [2, 4]

*Dactylorhizaincarnata* (L.) Soó [3, 5]

Dactylorhizaincarnatasubsp.cruenta (O.F.Müll.) P.D.Sell [3]

*Dactylorhizasalina* (Turcz.) Soó [1–11, 14]

*Dactylorhizaumbrosa* (Kar. & Kir.) Nevski [1, 3, 4, 7, 10, 14]

*Dactylorhizaviridis* (L.) R.M.Bateman [= *Coeloglossumviride* (L.) Hartm.] [1–7]

*Epipogiumaphyllum* Sw. [1, 2, 3, 4]

*Goodyerarepens* (L.) R.Br. [1, 2, 3, 4, 6]

*Gymnadeniaconopsea* (L.) R.Br. [≡ *Orchisconopsea* L.] [1–5]

*Herminiumalaschanicum* Maxim. [≡ *Peristylusalaschanicus* (Maxim.) N.Pearce & P.J.Cribb] [16]

*Herminiummonorchis* R.Br. [1–5, 8, 9, 10]

*Malaxismonophyllos* (L.) Sw. [≡ *Ophrysmonophyllos* L.] [1, 2, 3, 4, 5]

*Neottiacamtschatea* Sprengel [1, 2, 3, 7]

*Neottiapuberula* (Maxim.) Szlach. [≡ *Listerapuberula* Maxim.] [5]

*Orchismilitaris* L. [3, 4]

*Platantherabifolia* (L.) Rich. [1, 2, 3, 4]

*Platantherafuscescens* Kraenzl. [2, 3, 4, 5]

*Platantheraoligantha* Turcz. [1, 3]

*Ponerorchiscucullata* (L.) X.H.Jin [≡ *Neottianthecucullata* (L.) Schltr.] [1, 2, 3, 4]

*Spiranthesaustralis* Lindl. [2–5, 8, 9, 10]

**79. Orobanchaceae** Vent. (9 genera and 57 taxa)

*Boschniakiarossica* (Cham. & Schltdl.) B.Fedtsch. [2]

*pallida* (L.) Spreng. [1–9, 13]

*Cistanchedeserticola* Ma [7, 10–16]

*Cistanchefeddeana* K.S.Hao [9, 12, 13, 16]

*Cistanchelanzhouensis* Zhi Y.Zhang [12]

*Cistanchesalsa* (C.A.Mey.) Beck [12–16]

*Cymbariadaurica* L. [2–5, 7–13]

*Euphrasiaaltaica* Serg. [7]

*Euphrasiahirtella* Jord. [2, 3, 4, 5]

*Euphrasiamaximowiczii* Wettst. [2, 4, 5, 9]

*Euphrasiapectinata* Ten. [1–10, 13, 14]

*Euphrasiaschischkinii* Serg. [7]

*Euphrasiasyreitschikovii* Govor. [1, 2, 3, 5–8]

*Odontitesvulgaris* Moench [2–4, 7–11, 14]

*Orobancheamoena* C.A.Mey. [7, 14]

*Orobanchecaesia* Rchb. [= *Phelypaealanuginosa* C.A.Mey. ≡ *Orobanchelanuginosa* (C.A.Mey.) Beck] [1, 2, 3, 4]

*Orobanchecernua* Loefl. [7, 10, 13, 14]

*Orobanchecoerulescens* Steph. [≡ *Orobanchellacoerulescens* (Steph.) Piwow.] [5, 7–15]

Orobanchecoerulescensvar.albiflora Kuntze [= *Orobanchekorshinskyi* Novopokr.] [1–15]

*Orobanchepycnostachya* Hance [5]

*Pedicularisabrotanifolia* M.Bieb. [1, 3, 6, 7, 13, 14]

*Pedicularisachilleifolia* Steph. [1, 3, 6–8, 10, 14]

*Pedicularisaltaica* Steph. [6, 7, 10, 14]

*Pedicularisamoena* Adams [1–3, 6, 7, 13, 14]

*Pedicularisanthemifolia* Fisch. [1, 3, 6, 7, 13]

*Pediculariscompacta* Steph. [1, 2, 3, 6, 7]

*Pedicularisdolichorrhiza* Schrenk [7, 14]

*Pediculariselata* Willd. [3, 6, 7]

SE*Pedicularisfetisowii* Regel [14]

*Pedicularisfissa* Turcz. [2, 7]

*Pedicularisflava* Pall. [2–4, 6–11, 13–15]

SE*Pedicularisincarnata* L. [1]

*Pedicularislabradorica* Wirsing [1, 2, 3, 4]

*Pedicularislapponica* L. [1]

*Pedicularislasiostachys* Bunge [3, 6, 7]

*Pedicularislongiflora* Rudolph [1, 2, 3, 7, 11]

*Pedicularismoschata* Maxim. [6, 7, 10]

*Pedicularismyriophylla* Pall. [1–4, 6–8, 13]

*Pedicularisoederi* Vahl [1, 2, 3, 6, 7]

PedicularispalustrisL.subsp.karoi (Freyn) P.C.Tsoong [≡ *Pediculariskaroi* Freyn] [1–6, 8, 9, 10, 14]

*Pedicularisphysocalyx* Bunge [7]

*Pedicularisproboscidea* Steven [7]

*Pedicularisresupinata* L. [1–6, 8–10, 13]

*Pedicularisrhinanthoides* Schrenk [7]

*Pedicularisrubens* Steph. [1, 2, 3, 4, 5]

*Pedicularissceptrum-carolinum* L. [2, 3, 4, 5, 7]

*Pedicularissibirica* Vved. [1, 3, 7]

*Pedicularisspicata* Pall. [3, 4, 5]

*Pedicularisstriata* Pall. [1–5, 8, 9]

*Pedicularissudetica* Willd. [1, 2]

*Pedicularistristis* L [1, 2, 3, 6, 7, 8]

*Pedicularisuliginosa* Bunge [1–4, 6, 7, 10, 13]

*Pedicularisvenusta* Schangin [1–4, 6–11]

*Pedicularisverticillata* L. [1, 2, 3, 4, 5, 9]

*Pediculariswlassoviana* Steven [2]

*Rhinanthusserotinus* Oborny [2]

*Rhinanthussongaricus* (Sterneck) B.Fedtsch. [≡ Rhinanthusborbasii(Dörf.)Soósubsp.songaricus (Sterneck) Soó] [2, 3, 4]

**80. Oxalidaceae** R.Br. (1 genus and 1 species)

*Oxalisacetosella* L. [1, 2]

**81. Paeoniaceae** Raf. (1 genus and 3 species)

*Paeoniaanomala* L. [1, 2, 3, 4, 6, 7]

*Paeoniaintermedia* C.A.Mey. [6, 7]

*Paeonialactiflora* Pall. [2, 4, 5, 9]

**82. Papaveraceae** Juss. (6 genera and 30 species)

*Chelidoniummajus* L. [1–5, 7, 9]

*Corydalisadunca* Maxim. [6, 7, 13, 14, 15]

*Corydaliscapnoides* Pers. [2, 6, 7, 9, 14]

*Corydalisgrubovii* Mikhailova [6, 7]

*Corydalisimpatiens* Fisch. [1, 2, 3, 7]

*Corydalisinconspicua* Bunge [1, 2, 6, 7]

*Corydalispauciflora* Pers. [1, 2, 6, 13]

*Corydalissajanensis* Peschkova [≡ Corydalispauciflorasubsp.sajanensis (Peschkova) Mikhailova] [1]

*Corydalisschanginii* (Pall.) B.Fedtsch. [7, 14]

*Corydalissibirica* Pers. [1–4, 6–8, 10, 13]

*Corydalisstricta* Steph. [= *Corydalisgrubovii* Mikhailova] [6, 7, 13]

*Fumariaofficinalis* L. [7]

*Fumariaschleicheri* Soy.-Will. [7, 14]

*Glauciumelegans* Fisch. & C.A.Mey. [14]

*Glauciumsquamigerum* Kar. & Kir. [7, 14]

*Hypecoumerectum* L. [2–5, 8, 9, 11, 12]

*Hypecoumlactiflorum* (Kar. & Kir.) Pazii [3, 4, 6–16]

*Hypecoumleptocarpum* Hook.f. & Thomson [3]

E *Papaverbaitagense* Kamelin & Gubanov [6, 7, 14]

*Papavercanescens* Tolm. [1–7, 13]

*Papaverchakassicum* Peschkova [6, 7]

*Papaverlapponicum* (Tolm.) Nordh. [7]

*Papavernudicaule* L. [1–7, 9, 13]

*Papaverpseudocanescens* Popov [1, 3, 6, 7, 13]

*Papaverpseudotenellum* Grubov [7, 10, 13, 14]

*Papaverrefractum* (DC.) K.-F.Günther [≡ *Roemeriarefracta* DC.] [3, 14]

*Papaverrubroaurantiacum* (Fisch.) C.E.Lundstr. [1–10, 13]

SE*Papaversaichanense* Grubov [≡ Papaverrubroaurantiacumsubsp.saichanense (Grubov) Kamelin & Gubanov] [7, 13]

*Papaversmirnovii* Peschkova [≡ Papaverrubroaurantiacumsubsp.smirnovii (Peschkova) Kamelin & Gubanov] [4, 9]

*Papaversetosum* (Tolm.) Peschkova [≡ Papaverrubroaurantiacumsubsp.setosum Tolm.] [4]

**83. Phyllanthaceae** Martinov (1 genus and 1 species)

*Flueggeasuffruticosa* Baill. [5, 9]

**84. Plantaginaceae** Juss. (7 genera and 47 species)

*Callitrichehermaphroditica* L. [1, 3, 4, 5]

*Callitrichepalustris* L [1–5, 7, 9–11, 14]

*Hippurisvulgaris* L. [1–11, 13, 14]

*Lagotisintegrifolia* (Willd.) Schischk. [1, 2, 3, 7, 13]

*Linariaacutiloba* Fisch. [≡ LinariavulgarisMill.subsp.acutiloba (Fisch.) D.Y.Hong] [1–4, 6–8, 13, 14]

*Linariaaltaica* Fisch. [3, 6, 7, 10, 14]

*Linariaburiatica* Turcz. [1–6, 8, 9]

*Linariadebilis* Kuprian. [7, 14]

*Linariahepatica* Bunge [6, 7, 11, 13, 14]

*Linariaincompleta* Kuprian. [7]

*Linariamelampyroides* Kuprian. [3, 4, 5, 7, 9]

*Linariapedicellata* Kuprian. [6, 7, 10, 13, 14]

*Plantagoarachnoidea* Schrenk [= *Plantagolorata* (J.Z.Liu) Shipunov ≡ Plantagoarachnoideavar.lorata J.Z.Liu] [14]

*Plantagocornuti* Gouan [2, 9, 10]

*Plantagodepressa* Willd. [1–10, 12, 13]

*Plantagokomarovii* Pavlov [1, 3, 6, 7, 13]

*Plantagomajor* L. [2–14]

Plantagomaritimasubsp.ciliata Printz [= *Plantagosalsa* Pall.] [1–6, 8–11, 13, 14]

*Plantagominuta* Pall. [3, 7, 8, 10–15]

*Plantagopolysperma* Kar. & Kir. [10, 13, 15]

*Plantagourvillei* Opiz [2]

*Veronicaanagallis-aquatica* L. [1–4, 7–16]

*Veronicaanagalloides* Guss. [10]

*Veronicaarenosa* (Serg.) Boriss. [= *Veronicalaeta* auct. non Kar. & Kir.] [2, 3, 6, 7, 8, 14]

*Veronicabeccabunga* L. [3, 7, 8]

*Veronicabiloba* Schreb. [3, 6, 7, 14, 15]

*Veronicaciliata* Fisch. [1, 2, 3, 6, 7]

*Veronicadaurica* Steven [2, 4, 5, 8, 9]

*Veronicadensiflora* Ledeb. [2, 7]

*Veronicaferganica* Popov [6, 7, 14]

*Veronicahispidula* Boiss. & Huet [= *Veronicapusilla* Hohen. & Boiss.] [7]

*Veronicaincana* L. [1–11, 13]

*Veronicakrylovii* Schischkin [9]

*Veronicalinariifolia* Link [1–5, 8, 9]

*Veronicalongifolia* L. [1–4, 6, 7, 9, 10]

*Veronicamacrostemon* Bunge [1, 7]

*Veronicaoxycarpa* Boiss. [≡ Veronicaanagallis-aquaticasubsp.oxycarpa (Boiss.) A. Jelen] [7]

Veronicapinnatasubsp.nana Polozhij [7]

VeronicapinnataL.subsp.pinnata [2–4, 6–8, 10, 14]

*Veronicaporphyriana* Pavlov [7, 14]

*Veronicasajanensis* Printz [7]

E *Veronicasapozhnikovii* Kosachev [7, 14]

*Veronicascutellata* L. [7]

*Veronica×schmakovii* Kosachev [7]

*Veronica×smirnovii* Kosachev & D.A.German [7]

*Veronicastrumsibiricum* (L.) Pennell [2, 4, 5, 6, 7, 9]

*Veronicastrumtubiflorum* (Fisch. & C.A.Mey.) Soják [≡ *Veronicatubiflora* Fisch. & C.A.Mey.] [4]

**85. Plumbaginaceae** Juss. (4 genera and 20 taxa)

Armeriamaritimasubsp.sibirica (Turcz.) Nyman [≡ *Armeriasibirica* Turcz.] [3, 7]

*Goniolimoncallicomum* Boiss. [7, 14]

*Goniolimoneximium* Boiss. [7]

*Goniolimonkrylovii* A.V.Grebenjuk [7, 14]

*Goniolimonspeciosum* Boiss. [1–4, 6–15]

*Limoniumaureum* (L.) Hill [3–13, 15, 16]

*Limoniumbicolor* Kuntze [4, 6, 8, 9, 11–14]

*Limoniumchrysocomum* (Kar. & Kir.) Kuntze [3, 7, 10, 11, 13–15]

Limoniumchrysocomumsubsp.semenovii (Herder) Kamelin [7, 11, 13]

*Limoniumcongestum* Kuntze [6, 7, 10]

*Limoniumcoralloides* (Tausch) Lincz. [6, 10, 14]

*Limoniumflexuosum* Kuntze [1–4, 6–9, 12, 13]

*Limoniumgmelinii* Kuntze [6, 10, 14]

E *Limoniumgobicum* Ikonn.-Gal. [12]

E *Limoniumgrubovii* Lincz. [9]

E *Limoniumklementzii* Ikonn.-Gal. [7, 10, 15]

*Limoniummyrianthum* Kuntze [14]

*Limoniumsuffruticosum* Kuntze [14]

*Limoniumtenellum* Kuntze [4, 8, 9, 11–13, 15, 16]

*Plumbagellamicrantha* (Ledeb.) Spach [3, 4]

**86. Poaceae** Barnhart (58 genera and 229 taxa)

Note: The genus *Stipa* L. was recently revised which included a taxonomic key and species synopsis by Zhao et al (2019).

*Achnatherumcaragana* (Trin.) Nevski [= *Stipaconferta* Poir.] [7]

*Achnatherumconfusum* (Litv.) Tzvelev [≡ *Stipaconfusa* Litv.] [2, 3, 4]

SE*Achnatheruminebrians* (Hance) Keng [≡ *Stipainebrians* Hance] [7, 12, 13, 16]

SE*Achnatherumpelliotii* (Danguy) Röser & Hamasha [≡ *Stipapelliotii* Danguy] [7, 11–16]

*Achnatherumsibiricum* (L.) Keng [≡ *Stipasibirica* (L.) Lam.] [1–13]

*Aeluropuslittoralis* (Gouan) Parl. [10, 14, 15]

*Agropyroncristatum* (L.) Gaertn. [≡ *Bromuscristatus* L.] [1–7, 9–15]

*Agropyrondesertorum* Schult. [3, 6–9, 11–13, 15]

*Agropyronfragile* (Roth) P.Candargy [6, 8, 9, 11, 12, 14]

*Agropyronkrylovianum* Schischk. [≡ *Kengyiliakryloviana* (Schischk.) C.Yen, J.L.Yang & B.R.Baum] [4, 6, 7]

*Agropyronmichnoi* Roshev. [≡ Agropyroncristatum(L.)Gaertn.subsp.michnoi (Roshev.) Á.Löve] [1, 3–5, 8–10, 13, 14]

*Agropyronpumilum* (Steud.) P.Candargy [6, 10, 14]

*Agrostisclavata* Trin. [≡ Agrostisexaratasubsp.clavata (Trin.) T.Koyama] [1, 2, 3, 4, 5, 9]

*Agrostisdivaricatissima* Mez [= *Agrostismongolica* Roshev.] [2–13, 15]

*Agrostisgigantea* Roth [1–7, 9, 10, 13, 14]

*Agrostisstolonifera* L. [1, 3, 4, 7–10, 14]

SE*Agrostistuvinica* Peschkova [1, 2]

*Agrostisvinealis* Schreb. [= *Agrostistrinii* Turcz.] [1–10, 13]

*Alopecurusaequalis* Sobol. [1–10, 12]

*Alopecurusarundinaceus* Poir. [1–10, 12–14]

*Alopecurusbrachystachyus* M.Bieb. [1–10, 13]

*Alopecuruspratensis* L. [3, 4, 6, 8–10, 13, 14]

SE*Alopecurusturczaninovii* O.D.Nikif. [1–4, 6–8, 13]

*Anthoxanthumglabrum* (Trin.) Veldkamp [≡ *Hierochloeglabra* Trin.] [1–10]

*Anthoxanthummonticola* (Bigelow) Veldkamp [≡ *Holcusmonticola* Bigelow] [1–3, 6, 7, 10]

*Anthoxanthumnitens* (Weber) Y.Schouten & Veldkamp

[≡ *Poanitens* Weber = *Hierochloeodorata* (L.) P.Beauv.] [1–4, 6–10, 14]

*Anthoxanthumodoratum* L. [2, 3, 4, 5, 7]

*Arctagrostislatifolia* Griseb. [1, 2, 3]

*Arctopoaschischkinii* (Tzvelev) Prob. [≡ *Poaschischkinii* Tzvelev] [1, 7, 8]

*Arctopoatibetica* (Munro) Prob. [≡ *Poatibetica* Munro] [1, 3, 6–8, 10–15]

*Aristidaadscensionis* L. [3, 8–13, 15, 16]

*Arundinellahirta* (Thunb.) Tanaka [≡ *Poahirta* Thunb.] [4, 5, 9]

*Beckmanniasyzigachne* Fernald [1–6, 8–16]

*Brachypodiumpinnatum* (L.) P.Beauv. [≡ *Bromuspinnatus* L.] [3, 4]

*Bromusinermis* Leyss. [1–10, 12–15]

*Bromusjaponicus* Thunb. [6, 7, 10, 12, 14]

*Bromusoxyodon* Schrenk [7, 11, 14]

*Bromuspumpellianus* Scribn. [= *Bromuskorotkiji* Drobow] [3, 4, 7–11, 13]

*Bromusscoparius* L. [14]

*Bromussquarrosus* L. [10]

*Bromustectorum* L. [7, 14]

*Calamagrostisangustifolia* Kom. [= Calamagrostisangustifoliasubsp.tenuis (V.N.Vassil.) Tzvelev] [1–11, 13, 14]

*Calamagrostisepigejos* (L.) Roth [= Calamagrostisepigejossubsp.glomerata (Boiss. & Buhse) Tzvelev] [2, 4, 5, 9]

*Calamagrostisinexpansa* A.Gray [= Calamagrostisinexpansasubsp.micrantha (Kearney) Stebbins] [2, 3, 4, 5]

*Calamagrostiskorotkyi* Litv. [≡ *Deyeuxiakorotkyi* (Litv.) S.M.Phillips & W.L.Chen] [1, 2, 3, 4, 6]

SE*Calamagrostis×kuznetzovii* Tzvelev [4]

*Calamagrostislapponica* (Wahlenb.) Hartm. [≡ *Arundolapponica* Wahlenb.] [1–4, 6–8, 10, 13–15]

*Calamagrostismacilenta* Litv. [1–4, 7–13, 15]

*Calamagrostismacrolepis* Litv. [≡ Calamagrostisepigejossubsp.macrolepis (Litv.) Tzvelev] [2, 3, 4]

*Calamagrostisobtusata* Trin. [3, 4]

*Calamagrostispavlovii* (Roshev.) Roshev. [1–4, 7, 8, 10, 11]

*Calamagrostispseudophragmites* (Haller f.) Koeler [≡ *Arundopseudophragmites* Haller f.] [1–7, 9]

*Calamagrostispurpurea* (Trin.) Trin. [≡ *Arundopurpurea* Trin.] [2, 3, 4, 10]

SE*Calamagrostissajanensis* Malyschev [8, 9, 10, 13]

*Calamagrostissalina* Tzvelev [1–5, 8, 9, 13]

*Calamagrostisstricta* (Timm) Koeler [≡ *Arundostricta* Timm] [2]

*Catabrosaaquatica* (L.) P.Beauv. [2–4, 8–10, 13]

*Cenchrusflaccidus* (Griseb.) Morrone [≡ *Pennisetumflaccidum* Griseb.] [10, 12]

*Cinnalatifolia* (Trevir.) Griseb. [≡ *Agrostislatifolia* Trevir.] [2, 3, 4]

SE*Cleistogenescaespitosa* Keng [12]

SE*Cleistogenesfestucacea* Honda [= *Cleistogenesfoliosa* Keng] [4, 12, 13]

*Cleistogeneskitagawae* Honda [≡ *Kengiakitagawae* (Honda) Packer] [2–5, 8, 9]

*Cleistogenessongorica* (Roshev.) Ohwi [4, 7–16]

*Cleistogenessquarrosa* (Trin.) Keng [2–13]

*Colpodiumaltaicum* Trin. [1, 7]

DeschampsiacaespitosaP.Beauv.subsp.caespitosa [1–4, 6, 7, 8]

Deschampsiacaespitosasubsp.orientalis Hultén [1, 2, 3, 4, 5]

Deschampsiacaespitosasubsp.pamirica (Roshev.) Tzvelev [≡ *Deschampsiapamirica* Roshev.] [1, 3]

*Deschampsiakoelerioides* Regel [1, 2, 3, 6, 7]

*Echinochloacrus-galli* (L.) P.Beauv. [4, 7, 9, 12, 13]

*Elymusbungeanus* (Trin.) Melderis [2, 4, 6, 7, 12, 14, 15]

*Elymusconfusus* (Roshev.) Tzvelev [≡ *Roegneriaconfusa* (Roshev.) Nevski] [1–4, 7, 8, 13]

*Elymusdahuricus* Turcz. [1–10, 13]

*Elymusfedtschenkoi* Tzvelev [≡ *Roegneriafedtschenkoi* (Tzvelev) J.L.Yang & C.Yen] [7]

*Elymusgmelinii* (Ledeb.) Tzvelev [1–5, 7, 8, 9, 13]

SE*Elymuskarakabinicus* Kotukhov [7]

*Elymusmacrourus* (Turcz.) Tzvelev [= *Elymuskronokensis* (Kom.) Tzvelev, Elymuskronokensissubsp.subalpinus (Neuman) Tzvelev] [1, 4, 7]

*Elymusmutabilis* (Drobow) Tzvelev [= *Elymustransbaicalensis* (Nevski) Tzvelev = *Elymuspraecaespitosus* (Nevski) Tzvelev] [1, 2, 3, 4, 6, 7]

*Elymusnutans* Griseb. [3, 4, 9, 13]

*Elymuspendulinus* (Nevski) Tzvelev [= *Agropyronvernicosum* Nevski = *Elymusbrachypodioides* (Nevski) Peschkova] [4, 5, 7, 9, 12, 13, 16]

*Elymusreflexiaristatus* (Nevski) Melderis [= *Elymusaegilopoides* (Drobow) Vorosch.] [1–7, 12, 13, 14]

*Elymusrepens* (L.) Gould [= *Elytrigiarepens* (L.) Nevski] [2–11, 13–15]

*Elymusschrenkianus* (Fisch. & C.A.Mey.) Tzvelev [= *Elymuspamiricus* Tzvelev] [3, 4, 7, 13]

*Elymussibiricus* L. [1–10, 12–16]

*Elymusuralensis* (Nevski) Tzvelev [= Elymusuralensissubsp.komarovii (Nevski) Tzvelev] [2, 3, 4, 6, 13]

*Elymusvarius* (Keng) Tzvelev [4]

*Enneapogondesvauxii* P.Beauv. [3, 4, 6–15]

*Eragrostiscilianensis* (All.) Vignolo [10]

*Eragrostisminor* Host [2, 3, 4, 6–16]

*Eragrostispilosa* (L.) P.Beauv. [= Eragrostispilosasubsp.imberbis (Franch.) Tzvelev] [2, 4, 7–10, 12]

*Eremopyrumdistans* (K.Koch) Nevski [14]

*Festucaaltaica* Trin. [1, 3, 4, 6, 7]

*Festucabrachyphylla* Schult. & Schult.f. [1, 3, 6, 7, 13]

*Festucadahurica* V.I.Krecz. & Bobr. [2, 4, 5, 9]

*Festucaextremiorientalis* Ohwi [2]

SE*Festucahubsugulica* Krivot. [= *Festucasumneviczii* Serg.] [1]

*Festucajacutica* Drobow [4, 9]

*Festucakomarovii* Krivot. [1, 2]

*Festucakryloviana* Reverd. [1–4, 6, 7, 9, 13]

*Festucakurtschumica* E.B.Alexeev [7]

*Festucalenensis* Drobow [1–9, 13, 15]

*Festucalitvinovii* (Tzvelev) E.B.Alexeev [9]

*Festucaoreophila* Markgr.-Dann. [= Festucavalesiacasubsp.hypsophila (St.-Yves) Tzvelev] [1–4, 6, 7, 13, 14]

*Festucaovina* L. [= Festucaovinasubsp.sphagnicola (B.Keller) Tzvelev] [1–7, 9, 13]

SE*Festucapseudosulcata* Drobow [4]

*Festucarubra* L. [1–9, 13]

*Festucasibirica* Hack. [1–5, 8–10, 13]

*Festucatristis* Krylov & Ivanitzk. [3, 6, 7]

SE*Festucatschujensis* Reverd. [3, 6, 7, 10]

*Festucavalesiaca* Schleich. [1–10, 13]

*Festucavenusta* St.-Yves [1, 2, 3, 4]

*Glyceriaarundinacea* Kunth [1–10, 14]

*Glycerialithuanica* (Gorski) Gorski [3, 5]

*Glyceriaspiculosa* Roshev. [= *Glycerialongiglumis* Hand.-Mazz.] [4, 5, 9]

*Helictochloadahurica* (Kom.) Romero Zarco [≡ *Helictotrichondahuricum* (Kom.) Kitag.] [1, 4, 5]

*Helictochloahookeri* (Scribn.) Romero Zarco [= *Helictotrichonschellianum* (Hack.) Kitag.] [1–9, 13]

*Helictotrichondesertorum* (Less.) Pilg. [= *Helictotrichonaltaicum* Tzvelev] [3, 6, 7]

*Helictotrichonmongolicum* (Roshev.) Henrard [≡ *Avenastrummongolicum* (Roshev.) Roshev.] [1–3, 6, 7, 13]

*Helictotrichonpubescens* (Huds.) Pilg. [= Hordeumbrevisubulatumsubsp.turkestanicum (Nevski) Tzvelev] [3, 4, 7, 9]

*Hordeumbogdanii* Wilensky [3, 7, 9, 10, 14–16]

*Hordeumbrevisubulatum* Link [= Hordeumbrevisubulatumsubsp.turkestanicum (Nevski) Tzvelev] [1–16]

*Hordeumroshevitzii* Bowden [≡ *Critesionroshevitzii* (Bowden) Tzvelev] [1, 2, 4, 8–11, 13]

*Koeleriaaltaica* (Domin) Krylov [1–10, 13]

*Koeleriaasiatica* Domin [2, 8]

*Koeleriaglauca* DC. [4]

*Koelerialitvinowii* Domin [1, 3, 7, 13]

*Koeleriamacrantha* (Ledeb.) Schult. [1–5, 8–13]

Koeleriaspicatasubsp.mongolica (Hultén) Barberá, Quintanar, Soreng, & P.M.Peterson [≡ Trisetumspicatumsubsp.mongolicum Hultén] [1–4, 6, 7, 13]

SE*Koeleriathonii* Domin [5]

*Leymusangustus* (Trin.) Pilg. [≡ *Elymusangustus* Trin.] [3, 6, 7, 10–14, 16]

*Leymuschinensis* (Trin.) Tzvelev [1–6, 8–14]

SE*Leymusordensis* Peschkova [15]

*Leymuspaboanus* (Claus) Pilg. [3, 6–8, 10–14]

*Leymusracemosus* (Lam.) Tzvelev [3, 5, 6, 8–13]

*Leymusramosus* (K.Richt.) Tzvelev [≡ *Agropyronramosum* K.Richt.] [5, 9]

*Leymussecalinus* (Georgi) Tzvelev [= Leymussecalinusvar.mongolicus (Meld.) Tzvelev = *Leymusovatus* (Trin.) Tzvelev] [1–4, 6–16]

*Melicanutans* L. [1, 2, 4]

*Melicatranssilvanica* Schur [7]

*Melicaturczaninowiana* Ohwi [2, 3, 4, 5, 9]

*Melicavirgata* Turcz. [1–5, 8–10, 12, 13]

*Miliumeffusum* L. [2]

*Nardusstricta* L. [3]

*Neotriniasplendens* (Trin.) M.Nobis [≡ *Achnatherumsplendens* (Trin.) Nevski ≡ *Stipasplendens* Trin.] [2–5, 7–16]

*Phalarisarundinacea* L. [1–5, 7, 9, 10, 14]

*Phleumalpinum* L. [7]

*Phleumphleoides* (L.) H.Karst. [2–4, 6–8, 10]

*Phragmitesaustralis* (Cav.) Steud. [1–16]

*Piptatherumsongaricum* (Trin. & Rupr.) Roshev. [7, 14]

*Poaalpina* L. [1, 3, 6, 7, 15]

*Poaalta* Hitchc. [= *Poamongolica* (Rendle) Keng] [2, 5]

*Poaaltaica* Trin. [≡ Poaglaucasubsp.altaica (Trin.) Olonova & G.H.Zhu] [1–3, 6, 7, 10, 13]

*Poaangustifolia* L. [≡ Poapratensissubsp.angustifolia (L.) Dumort.] [2, 3, 4, 5, 9]

*Poaannua* L. [≡ *Ochlopoaannua* (L.) H.Scholz] [2, 7, 9]

*Poaargunensis* Roshev. [1–11, 13, 15]

PoaattenuataTrin.subsp.attenuata . [1–4, 6–10, 12–14]

Poaattenuatasubsp.botryoides (Trin.) Tzvelev [1, 3–9, 13]

Poaattenuatasubsp.dahurica (Trin.) Gubanov [≡ *Poadahurica* Trin.] [1–4, 7–13]

Poaattenuatasubsp.tshuensis (Serg.) Olonova [≡ *Poaargunensisf.tshuensis* Serg.] [1–4, 6–10, 12–15]

*Poaglauca* Vahl [1, 3, 6, 7, 13]

*Poaircutica* Roshev. [4]

SE*Poakenteica* Ivanova [2, 3]

*Poakrylovii* Reverd. [≡ Poaurssulensissubsp.krylovii (Reverd.) Olonova] [2, 3, 4, 7, 8]

*Poanemoralis* L. [1–5, 8, 9]

*Poapalustris* L. [1–5, 7, 8, 9, 13]

*Poapratensis* L. [= Poapratensissubsp.sabulosa (Turcz.) Tzvelev] [1–10, 13, 14]

*Poaraduliformis* Prob. [2, 7]

*Poasibirica* Roshevitz [1–7, 10, 13]

*Poasmirnowii* Roshev. [1, 3, 4, 6, 7]

*Poasubfastigiata* Trin. [1–6, 8–10, 13]

*Poasupina* Schrad. [3, 4, 7]

*Poatianschanica* Hack. [1–10, 13]

*Poatrivialis* L. [1]

*Poaurssulensis* Trin. [2, 3, 5]

*Poaveresczaginii* Tzvelev [7]

Poaversicolorsubsp.reverdattoi (Roshev.) Olonova & G.H.Zhu [≡ *Poareverdattoi* Roshev.] [1, 3, 5–10, 13, 14]

PoaversicolorBessersubsp.versicolor [= Poaversicolorsubsp.stepposa (Krylov) Tzvelev] [1–9, 11, 13]

*Polypogonmaritimus* Willd. [11, 15]

*Polypogonmonspeliensis* (L.) Desf. [7, 10, 11, 13, 15, 16]

SE*Psammochloavillosa* (Trin.) Bor [3, 9–13, 16]

*Psathyrostachysjuncea* (Fisch.) Nevski [3, 6, 7, 10, 12–15]

*Psathyrostachyslanuginosus* (Trin.) Nevski [7, 14]

*Ptilagrostismongholica* (Turcz.) Griseb. [≡ *Stipamongholica* Turcz.] [1, 2, 3, 4, 7]

*Puccinelliaaltaica* Tzvelev [14]

*Puccinelliadistans* (Jacq.) Parl. [11, 13, 14]

SE*Puccinelliafilifolia* (Trin.) Tzvelev [8, 12]

*Puccinelliahackeliana* (V.I.Krecz.) V.I.Krecz. [7]

*Puccinelliahauptiana* (V.I.Krecz.) Kitag. [3–11, 13, 14]

*Puccinelliamacranthera* V.I.Krecz. [2–5, 7–10, 12, 14]

*Puccinelliamanchuriensis* Ohwi [3]

*Puccinellianudiflora* (Hack.) Tzvelev [7, 13]

SE*Puccinelliaprzewalskii* Tzvelev [10]

*Puccinelliaschischkinii* Tzvelev [5, 8, 10, 12, 13, 15]

*Puccinelliatenuiflora* Scribn. & Merr. [= *Puccinelliakreczetoviczii* Bubnova] [1–16]

*Schismusarabicus* Nees [4, 7, 11, 14]

Schizachnepurpurascenssubsp.callosa (Turcz.) T.Koyama & Kawano [2, 3, 4, 5]

*Scolochloafestucacea* Link [4]

*Sibirotrisetumsibiricum* (Rupr.) Barberá [≡ *Trisetumsibiricum* Rupr.] [1–5, 7–10, 13]

*Spodiopogonsibiricus* Trin. [2, 3, 4, 5, 8, 9]

*Sporobolusaculeatus* (L.) P.M.Peterson [≡ *Crypsisaculeata* (L.) Aiton] [10–13, 15]

*Sporobolusschoenoides* (L.) P.M.Peterson [≡ *Crypsisschoenoides* Lam.] [3, 10, 14]

E *Stipaaustromongolica* M.Nobis [10]

*Stipabaicalensis* Roshev. [1–5, 7, 8, 9, 12]

*Stipabreviflora* Griseb. [7, 8, 9, 12, 13]

*Stipacapillata* L. [3, 4, 7, 10, 12]

StipacaucasicaSchmalh.subsp.caucasica [7, 10–14, 16]

Stipacaucasicasubsp.desertorum (Roshev.) Tzvelev [10]

*Stipaconsanguinea* Trin. & Rupr. [2, 7, 10]

Stipaglareosaf.pubescens P.A.Smirn. [4–12]

*Stipaglareosa* P.A.Smirn. [≡ Stipacaucasicasubsp.glareosa (P.A.Smirn.) Tzvelev] [3, 6–16]

*Stipagobica* Roshev. [3, 4, 6–16]

*Stipagrandis* P.A.Smirn. [3, 4, 5, 8, 9]

E *Stipakhovdensis* L.Q.Zhao [3, 6]

*Stipakirghisorum* P.A.Smirn. [3, 7, 10, 14]

*Stipaklemenzii* Roshev. [3, 4, 6, 8, 9–13]

*Stipakrylovii* Roshev. [1–14]

*Stipamongolorum* Tzvelev [7, 8, 10, 11, 12]

*Stipaorientalis* Trin. [1, 3, 6, 7, 10, 11, 14]

*Stipapennata***L.** subsp. pennata [3, 4]

Stipapennatasubsp.sabulosa (Pacz.) Tzvelev [10]

*Stipasareptana* A. Beck. [3, 7, 10, 11, 13]

*Stipasczerbakovii* Kotuch. [7]

*Stipatianschanica* Roshev. [= Stipatianschanicasubsp.gobica (Roshev.) D.F. Cui = Stipatianschanicavar.klemenzii (Roshev.) Norl.] [3, 4, 6–16]

*Stipazalesskii* Wilensky [3, 7, 10]

*Timouriasaposhnikowii* Roshev. [= *Stipasaposhnikowii* (Roshev.) Kitag.] [13, 15]

*Tragusmongolorum* Ohwi [12, 13]

*Tripogonchinensis* Hack. [2, 4, 5, 8, 9, 12]

*Tripogonpurpurascens* Duthie [12, 13, 16]

*Trisetumaltaicum* Roshev. [1, 2, 3, 6, 7]

*Zizanialatifolia* Turcz. [9]

**87. Polemoniaceae** Juss. (2 genera and 4 species)

*Phloxsibirica* L. [1, 4]

*Polemoniumboreale* Adams [1, 3, 6]

*Polemoniumchinense* Brand [1–7, 9]

*Polemoniumpulchellum* Bunge [1, 3, 6]

**88. Polygalaceae** Hoffmanns. & Link (1 genus and 3 species)

*Polygalacomosa* Schkuhr [= *Polygalahybrida* DC.] [1–4, 6, 7, 14]

*Polygalasibirica* L. [1, 2, 3, 4, 5, 9]

*Polygalatenuifolia* Willd. [1–5, 8, 9, 12, 13]

**89. Polygonaceae** Juss. (11 genera and 63 taxa)

*Atraphaxisbracteata* Losinsk. [3, 6, 7, 9, 10–16]

*Atraphaxiscompacta* Ledeb. [13, 14, 15]

*Atraphaxisfrutescens* (L.) K.Koch [3, 5, 6, 7, 9–16]

E *Atraphaxiskamelinii* Yurtseva [14]

*Atraphaxispungens* Jaub. & Spach [2–16]

*Atraphaxisspinosa* L. [14, 15]

*Atraphaxisvirgata* (Regel) Krassn. [7, 10, 12, 14,–16]

*Bistortaelliptica* (Willd.) V.V.Petrovsky [1, 2, 3, 6, 7]

SE*Calligonumebinuricum* Ivanova [14, 15]

*Calligonumjunceum* (Fisch. & C.A.Mey.) Litv. [14, 15]

*Calligonumlitwinowi* Drobow [= *Calligonumgobicum* Losinsk.] [14, 15]

*Calligonummongolicum* Turcz. [7, 10–16]

*Fallopiaconvolvulus* (L.) Á.Löve [≡ *Polygonumconvolvulus* L.] [2–5, 8–10, 12, 15]

*Fallopiadumetorum* (L.) Holub [5]

Knorringiasibirica(Laxm.)Tzvelevsubsp.sibirica [1–16]

Knorringiasibiricasubsp.ubsunurica Tzvelev [10]

*Koenigiaislandica* L. [1–3, 6, 7, 10, 13]

*Oxyriadigyna* Hill [1, 2, 3, 6, 7, 13]

*Persicariaalpina* Gross. [1–4, 6, 7, 8, 14]

*Persicariaamphibia* (L.) Delarbre [1, 3–12, 14]

*Persicariabistorta* Samp. [2]

*Persicariabungeana* Nakai [1–3, 5, 7–10, 13]

*Persicariahydropiper* (L.) Delarbre [≡ *Polygonumhydropiper* L.] [2–4, 6–8, 10, 13, 14]

*Persicarialapathifolia* (L.) Delarbre [≡ *Polygonumochreatum* L.] [1–16]

*Persicaria longiseta var. rotundata* (A.J.Li) B.Li [≡ Polygonumlongisetumvar.rotundatum A.J.Li] [2, 4, 8]

*Persicariaminor* (Huds.) Opiz [10, 14]

*Persicariasagittata* (L.) H.Gross [2, 3, 4, 5, 8, 9]

*Persicariavivipara* (L.) Ronse Decr. [1–4, 6–8, 10, 13, 14]

*Polygonumabbreviatum* Kom. [1, 2, 7]

*Polygonumalopecuroides* Turcz. [1–6, 8]

*Polygonumangustifolium* Pall. [1–5, 7–9, 11, 13]

*Polygonumarenastrum* Boreau [1, 3, 7, 10, 14]

*Polygonumargyrocoleon* Steud. [7, 10, 11, 13–15]

*Polygonumaviculare* L. [1–5, 7–14, 16]

*Polygonumcognatum* Meisn. [3, 4, 6, 7, 8, 10]

*Polygonumdivaricatum* L. [1–5, 8, 9]

*Polygonumellipticum* Willd. [1, 2, 3, 6, 7]

*Polygonumhumifusum* C.Merck [3]

*Polygonumintramongolicum* Borodina [12, 13]

*Polygonumnovoascanicum* Klokov [14]

*Polygonumpatulum* M.Bieb. [3, 7, 9, 10, 14, 15]

*Polygonumpolycnemoides* Jaub. & Spach [7, 14]

*Polygonumsericeum* Pall. [2, 3, 4, 8, 9]

*Polygonumtenuissimum* A.I.Baranov & Skvortsov [9]

*Polygonumvalerii* A.K.Skvortsov [2, 4, 5, 8]

*Polygonumvolchovense* Tzvelev [7]

*Rheumcompactum* L. [1–4, 6, 7, 12–14]

*Rheumnanum* Siev. [7, 8, 10–16]

*Rheumrhabarbarum* L. [= *Rheumundulatum* L.] [1–5, 7–9, 12–14]

SE*Rheumuninerve* Maxim. [13]

*Rumexacetosa* L. [1, 2, 3, 6, 7]

*Rumexacetosella* L. [1–5, 8, 9]

*Rumexaquaticus* L. [1–10, 14]

*Rumexcrispus* L. [1, 7, 9, 10, 14]

*Rumexgmelinii* Turcz. [2, 3, 4, 5, 8, 9]

*Rumexmaritimus* L. [2–5, 8–11, 14]

*Rumexmarschallianus* Rchb. [6, 8, 9, 10, 11]

*Rumexpatientia* L. [7, 9, 13, 14]

*Rumexpopovii* Pachom. [10, 13]

*Rumexpseudonatronatus* (Borbás) Murb. [11, 13]

*Rumexsimilans* Rech.f. [2–4, 6, 7, 10–12, 14, 15]

*Rumexstenophyllus* Ledeb. [2–4, 6, 7, 9, 10, 14, 15]

*Rumexthyrsiflorus* Fingerh. [1–14]

**90. Potamogetonaceae** Bercht. & J.Presl (3 genera and 18 taxa)

*Potamogetonangustifolius* Bercht. & J.Presl [10]

Potamogetonalpinussubsp.tenuifolius (Raf.) Hulten [3, 4, 9, 10, 12]

*Potamogetonberchtoldii* Fieber [3, 6, 7, 9]

*Potamogetoncompressus* L. [1, 8, 9, 10, 14]

*Potamogetoncrispus* L. [1, 8, 10]

*Potamogetonfriesii* Rupr. [5, 8, 9]

*Potamogetongramineus* L. [1–10, 14]

*Potamogetonlucens* L. [1, 8, 10]

*Potamogetonmandschuriensis* A.Benn. [5]

*Potamogetonnatans* L. [1, 5–7, 10, 11]

*Potamogetonobtusifolius* Mert. & W.D.J.Koch [1, 3, 5]

*Potamogetonperfoliatus* L. [1–12, 14, 16]

*Potamogetonpraelongus* F.Muell. [1–5, 8, 9]

*Potamogetonpusillus* L. [1–6, 8–11, 13, 14]

*Stuckeniafiliformis* (Pers.) Börner [≡ *Potamogetonfiliformis* Pers.] [1, 3, 6–11, 13, 15]

*Stuckeniapectinata* (L.) Börner [≡ *Potamogetonpectinatus* L.] [1–10, 11]

*Stuckeniavaginata* Holub [1, 3–8, 10, 11, 13]

*Zannichelliapalustris* L. [= Zannichelliapalustrissubsp.pedicellata (Rosén & Wahlenb.) Hook.f.] [3, 4, 7–11, 15]

**91. Primulaceae** Batsch (3 genera and 28 taxa)

Note: The genus *Primula* L. was recently revised by Baasanmunkh et al. (2020a).

*Androsacefedtschenkoi* Ovcz. [1, 6, 7, 13]

*Androsacefiliformis* Retz. [1, 2, 3, 4, 5, 9]

*Androsacegmelinii* Gaertn. [2, 3, 9, 11]

*Androsaceincana* Lam. [1–9, 13]

*Androsacelactiflora* Fisch. [= *Androsaceamurensis* Prob.] [1–4, 6, 7, 9, 14]

*Androsacelehmanniana* Spreng. [= *Androsacebungeana* Schischk. & Bobrov] [1, 2, 3, 6, 7, 9]

*Androsacelongifolia* Turcz. [5, 9]

*Androsacemaxima* L. [2–4, 6–10, 13–15]

*Androsaceovczinnikovii* Schischk. & Bobrov [3, 6, 7]

*Androsaceseptentrionalis* L. [1–9, 12–14]

AndrosacevillosaL.var.dasyphylla (Bunge) Kar. & Kir. [≡ *Androsacedasyphylla* Bunge] [1, 2, 3, 6, 7, 13]

*Lysimachiadavurica* Ledeb. [2, 3, 4, 5, 9, 14]

*Lysimachiaeuropaea* (L.) U.Manns & Anderb. [≡ *Trientaliseuropaea* L.] [1, 2, 3, 4, 5]

*Lysimachiamaritima* (L.) Galasso [≡ *Glauxmaritima* L.] [1–16]

*Lysimachiathyrsiflora* L. [≡ *Naumburgiathyrsiflora* (L.) Rchb.] [2–5, 9, 10, 14]

*Primulaalgida* Adams [1–3, 6, 7, 11, 13]

*Primulabukukunica* Kovt. [7, 11, 13]

*Primulacortusoides* L. [3]

*Primulafarinosa* L. [1–4, 6, 7, 10, 13, 15]

*Primulalongiscapa* Ledeb. [3, 6, 7, 10, 13, 14]

Primulamatthiolisubsp.altaica (Losinsk.) Kovt. [≡ *Cortusaaltaica* Losinsk.] [1, 2, 3, 7, 13, 14]

Primulamatthiolisubsp.brotheri (Pax) Kovt. [≡ Cortusamatthiolif.brotheri Pax] [7]

*Primulamaximowiczii* Regel [5]

Primulanivalissubsp.nivalis Pall. [1, 2, 3, 6, 7, 10]

Primulanivalissubsp.turkestanica (J.N.Haage & E.Schmidt) Kovt. [1, 3]

Primulanivalissubsp.xanthobasis (Fed.) Halda [1, 2, 3]

*Primulanutans* Georgi [1–7, 9, 10, 11]

*Primulaserrata* Georgi [1–5, 7–10, 13]

**92. Ranunculaceae** Juss. (20 genera and 156 taxa)

Note: In regards to the phylogeny and position of *Actea* and *Cimcifuga*, we follow Compton et al. (1998). *Anemone* is considered here according to Hoot et al.(2012) with some changes leaving *Pulsatilla* at generic level (Sramkó et al. 2019). The taxonomy of the tribe Ranunculae is given according to Emadzade et al.(2010) and Wang et al. (2014). The taxonomical positions of some taxa have been changed according to taxonomic works (Solovjev 1998; Erst 2007). Previously, four species of *Batrachium* were recorded in Mongolia which are treated as a synonym of *Ranunculus* by Wiegleb et al. (2017). Taxonomic revision of the genus *Aquilegia* L. was carried out by Erst et al. (2016).

*Aconitumambiguum* Rchb. [1, 2, 3, 4, 10]

*Aconitumanthoroideum* DC. [3, 7]

*Aconitumbaicalense* Turcz. [≡ AconitumambiguumRchb.subsp.baicalense (Turcz.) Vorosch.] [2, 3, 4, 5, 9]

*Aconitumbarbatum* Patr. [1–4, 6–8, 10, 11, 13]

*Aconitumbiflorum* Fisch. [3, 7]

*Aconitumcoreanum* (H.Lév.) Rapaics [5]

*Aconitumdecipiens* Vorosch. & Anfalov [3, 7]

*Aconitumglandulosum* Rapaics [=*Aconitumaltaicum* Steinb. = *Aconitumsmirnovii* Steinb.] [1–4, 6, 7, 13, 14]

E *Aconitumgubanovii* Luferov & Vorosch. [7, 14]

E *Aconitumkamelinii* A.A.Solovjev [= *Aconitumchasmanthum* Stapf] [3, 13]

SE*Aconitumkhanminthunii* A.A.Solovjev & Shmakov [3, 6, 7, 11, 13]

*Aconitumkusnezoffii* Rchb. [= *Aconitumbirobidshanicum* Vorosch.] [5, 9]

*Aconitumleucostomum* Vorosch. [6, 7, 8, 10]

*Aconitummacrorhynchum* Turcz. [4]

SE*Aconitumpaskoi* Vorosch. [2, 3]

*Aconitumranunculoides* Turcz. [4]

SE*Aconitumrubicundum* (Ser.) Fisch. [≡ Aconitumseptentrionalesubsp.rubicundum (Ser.) Vorosch.] [1]

*Aconitumseptentrionale* Koelle [1–4, 6, 7, 8]

SE*Aconitumturczaninowii* Vorosch. [2, 3, 4, 5, 9]

*Aconitumvolubile* Koelle [2, 3, 7]

*Actaeacimicifuga* L. [≡ *Cimicifugafoetida* L.] [1, 2, 3, 4]

*Actaeadahurica* (Turcz.) Franch. [≡ *Cimicifugadahurica* (Turcz.) Maxim.] [2, 5, 9]

*Actaeaerythrocarpa* (Fisch.) Kom. [1, 2, 3, 4]

*Actaeasimplex* Prantl [5]

*Adonisapennina* L. [= *Adonissibirica* Patrin] [1, 2, 3, 4, 6, 8]

E *Adonismongolica* Simonovich [1, 2, 3, 4, 8]

*Anemonastrumcrinitum* (Juz.) Holub [≡ Anemonenarcissiflorasubsp.crinita (Juz.) Kitag.] [1, 2, 3, 4, 6, 7]

*Anemonastrumdichotomum* (L.) Mosyakin [≡ *Anemonedichotoma* L.] [2, 3, 4, 5, 9]

*Anemonastrumobtusilobum* (D.Don) Mosyakin [≡ *Anemoneobtusiloba* Lindl.] [3]

*Anemonastrumsibiricum* (L.) Holub [≡ *Anemonesibirica* L.] [1, 2, 4]

*Anemonereflexa* Steph. [2, 3, 4]

*Anemonesylvestris* L. [≡ *Anemonoidessylvestris* (L.) Galasso] [1–7, 9]

*Aquilegiaamurensis* Kom. [2]

*Aquilegiaaradanica* Shaulo & Erst [4]

E *Aquilegiadaingolica* Erst & Shaulo [7]

*Aquilegiaganboldii* Kamelin & Gubanov [5]

*Aquilegiaglandulosa* Fisch. [1, 4, 6, 7]

E *Aquilegiagrubovii* Erst [1, 2, 3, 4]

*Aquilegiajucunda* Fisch. & Lallem. [1]

*Aquilegiasibirica* Lam. [1, 2, 3, 4, 6, 7]

*Aquilegiaviridiflora* Pall. [2–5, 7–10, 12, 13]

*Aquilegiaxinjiangensis* Erst [7]

*Callianthemumangustifolium* Witasek [7]

*Callianthemumisopyroides* Witasek [1, 2, 3]

*Callianthemumsajanense* Witasek [1, 3, 7]

*Calthamembranacea* (Turcz.) Schipcz. [5]

*Calthanatans* (Pall.) Deyl & Sojak [1–5, 8, 10, 11]

*Calthapalustris* L. [1–5, 9–11]

*Ceratocephalatesticulata* (Crantz) Besser [7]

*Clematisaethusifolia* Turcz. [9]

*Clematisbrevicaudata* DC. [5, 9]

SE*Clematisfruticosa* Turcz. [11–13, 15, 16]

*Clematisglauca* Willd. [3, 7, 10, 14, 15]

*Clematishexapetala* Pall. [2, 4, 5, 8, 9]

*Clematisintricata* Bunge [7–14, 16]

*Clematismacropetala* Ledeb. [9]

*Clematisochotensis* (Pall.) Poir. & Lam. [4]

*Clematisorientalis* L. [15]

*Clematissibirica* (L.) Mill. [1–4, 6–8, 10, 13]

*Clematissongarica* Siev. [7, 11–16]

SEClematistanguticasubsp.mongolica Grey-Wilson [2]

ClematistanguticaKorsh.subsp.tangutica [2–4, 7, 8, 10, 13–15]

*Delphiniumaltaicum* Nevski [6, 7, 13, 14]

*Delphiniumbarlykense* Lomon. & Khanm. [1, 6, 7]

E *Delphiniumchangaicum* N.Friesen [3, 13]

*Delphiniumcheilanthum* Fisch. [1–4, 6, 7, 8, 13]

*Delphiniumcrassifolium* Schrad. [1, 2, 3, 5, 6, 7]

*Delphiniumdictyocarpum* DC. [7]

SE*Delphiniumdissectum* Huth [1, 3, 4, 8]

*Delphiniumelatum* L. [1, 3, 6, 7]

*Delphiniumgrandiflorum* L. [1–5, 9, 13]

E *Delphiniumgubanovii* N.Friesen [7]

*Delphiniumiliense* Huth [14]

DelphiniuminconspicuumSerg.subsp.inconspicuum [3, 6, 7, 14]

E Delphiniuminconspicuumsubsp.mongolicum A.L.Ebel [7]

SE*Delphiniummalyschevii* N.Friesen [1]

*Delphiniummirabile* Serg. [6, 7]

SE*Delphiniumsajanense* Jurtzev [1]

*Delphiniumtriste* Fisch. [1, 2, 3, 4, 8, 13]

*Delphiniumukokense* Serg. [6, 7]

*Halerpestessalsuginosa* Greene [1–4, 6–15]

*Halerpestessarmentosa* (Adams) Kom. & Klob.-Alis [3, 4, 6–16]

*Isopyrumanemonoides* Kar. & Kir. [7]

*Leptopyrumfumarioides* Rchb. [1–4, 6–9, 13]

*Oxygraphisglacialis* (Fisch.) Bunge [1, 2, 3, 6, 7, 13]

*Paraquilegiaanemonoides* Ulbr. [1, 6, 7]

*Pulsatillaambigua* Turcz. [1–4, 6, 7, 13]

SE*Pulsatillabungeana* C.A.Mey. [= Pulsatillabungeanavar.astragalifolia (Pobed.) Grubov] [1–11, 13]

*Pulsatillacampanella* Fisch. [1, 3, 6, 7, 14]

*Pulsatilladahurica* (Fisch.) Spreng. [3, 9]

*Pulsatillamultifida* (G.Pritz.) Juz. [≡ Pulsatillapatenssubsp.multifida (G.Pritz.) Zämelis] [3, 4, 6, 7]

Pulsatillapatens(L.)Mill.subsp.flavescens (Zucc.) Zämelis [≡ *Pulsatillaflavescens* (Zucc.) Juz.] [1, 2, 3, 4, 5, 7]

*Pulsatillatenuiloba* (Hayek) Juz. [2, 3, 4, 9]

*Pulsatillaturczaninovii* Krylov & Serg. [1–6, 8, 9]

*Ranunculusacris* L. [1–5, 7, 8, 9, 10]

*Ranunculusaltaicus* Laxm. [1, 2, 3, 6, 7]

*Ranunculusaquatilis* L. [3, 5, 9]

E *Ranunculusarschantynicus* Kamelin, Shmakov & S.V.Smirn. [7, 14]

*Ranunculuschinensis* Bunge [2, 3, 4, 6, 10]

*Ranunculuscircinatus* Sibth. [3, 4, 5, 9, 10]

*Ranunculusconfervoides* (Fr.) Fr. [= Ranunculustrichophyllussubsp.eradicatus (Laest.) C.D.K.Cook] [2, 5–10]

*Ranunculusgmelinii* DC. [1, 2, 4, 5, 9]

*Ranunculusgobicus* Maxim. [13]

*Ranunculusgrandifolius* C.A.Mey. [3]

*Ranunculuskauffmannii* Clerc [≡ *Batrachiumkauffmannii* (Clerc) Krecz.] [2, 7]

*Ranunculuslapponicus* L. [1, 2, 3, 6, 7, 13]

SE*Ranunculuslasiocarpus* C.A.Mey. [1, 3, 6, 7]

*Ranunculuslingua* L. [14]

*Ranunculuslongicaulis* C.A.Mey. [1–3, 6, 7, 11, 14]

*Ranunculusmongolicus* (Krylov) Serg. [≡ *Batrachiummongolicum* Serg.] [3, 6, 7, 10, 11]

*Ranunculusmonophyllus* Ovcz. [1–7]

*Ranunculusnatans* C.A.Mey. [1–4, 6–10, 13, 15]

*Ranunculuspedatifidus* Sm. [= *Ranunculusrigescens* Turcz.] [1–7, 9, 13, 14]

*Ranunculuspolyanthemos* L. [6]

RanunculuspropinquusC.A.Mey.subsp.propinquus [1–4, 6–8, 10]

Ranunculuspropinquusvar.subborealis (Tzvel.) Luferov [1, 2, 3, 4]

*Ranunculuspseudohirculus* Schrenk [1–3, 6, 7, 13, 14]

SE*Ranunculuspseudomonophyllus* Timokhina [1, 2]

*Ranunculuspulchellus* C.A.Mey. [1–4, 6–10, 13]

*Ranunculusradicans* C.A.Mey. [1–8, 10, 13]

*Ranunculusrepens* L. [2–9]

*Ranunculusreptans* L. [1, 2, 4, 7, 10]

E *Ranunculussapozhnikovii* Schegol. [7]

*Ranunculussceleratus* L. [1–9, 11, 13, 14]

SE*Ranunculusschmakovii* Erst [7]

*Ranunculussmirnovii* Ovcz. [2]

Ranunculussulphureussubsp.exaltatus Erst [7]

*Ranunculustanguticus* (Maxim) Ovcz. [3, 6]

*Ranunculustrautvetterianus* Regel [7]

*Ranunculustrichophyllus* Chaix [≡ *Batrachiumtrichophyllum* (Chaix) Bosch = *Batrachiumdivaricatum* (Schrank) Schur] [1–11, 14]

SE*Ranunculusturczaninovii* (Luferov) Vorosch. [2]

SE*Ranunculustuvinicus* Erst [7]

*Thalictrumalpinum* L. [1–4, 6, 7, 13]

*Thalictrumbaicalense* Turcz. [2, 5]

*Thalictrumcontortum* L. [= Thalictrumaquilegiifoliumvar.sibiricum Regel & Tiling] [5, 9]

*Thalictrumfoetidum* L. [1–4, 6–10, 13, 14]

*Thalictrumisopyroides* C.A.Mey. [7, 14]

SEThalictrumminussubsp.appendiculatum (C.A.Mey.) Gubanov [≡ *Thalictrumappendiculatum* C.A.Mey.] [3]

Thalictrumminussubsp.elatum (Jacq.) Stoj. & Stef. [= Thalictrumminussubsp.kemense (Fries) Cajander] [3]

ThalictrumminusL.subsp.minus [1–10, 13, 14]

*Thalictrumpetaloideum* L. [1, 2, 3, 4, 5, 9]

SE*Thalictrumschischkinii* N.Friesen [= *Thalictrumaltaicum* (Schischk.) Serg.] [7]

*Thalictrumsimplex* L. [1–10, 13, 14]

*Thalictrumsquarrosum* Steph. [2, 3, 4, 5, 8, 9]

*Trolliusaltaicus* C.A.Mey. [6, 7, 14]

*Trolliusasiaticus* L. [1–4, 6, 7, 9, 13]

*Trolliusaustrosibiricus* Erst & Luferov [7]

*Trolliuschinensis* Bunge [7]

*Trolliusdschungaricus* Regel [14]

*Trolliusledebourii* Rchb. [2, 3, 4, 5, 9]

*Trolliuslilacinus* Bunge [1, 2, 6, 7]

*Trolliussajanensis* (Malyschev) Sipliv. [1]

*Trolliussibiricus* Schipcz. [5]

*Trolliusvicarius* Sipliv. [5]

**93. Rhamnaceae** Juss. (1 genus and 5 species)

*Rhamnusdavurica* Pall. [2, 4]

*Rhamnuserythroxylon* Pall. [2–4, 8, 9, 12, 13]

*Rhamnusmaximovicziana* J.J.Vassil. [13, 16]

*Rhamnusparvifolia* Bunge [2, 4, 5, 9]

*Rhamnusutilis* Decne. [4, 5, 9]

**94. Rosaceae** Juss. (28 genera and 168 taxa)

*Agrimoniapilosa* Ledeb. [1–6, 9]

*Alchemillaargutiserrata* H.Lindb. [7]

E *Alchemillachangaica* V.N.Tikhom. [1, 3]

*Alchemillacircularis* Juz. [7]

*Alchemillacyrtopleura* Juz. [3, 7]

*Alchemillaflavescens* Buser [3]

*Alchemillagracilis* Pax [3]

*Alchemillagubanovii* V.N.Tikhom. [2, 3]

*Alchemillahebescens* Juz. [2, 3, 7]

*Alchemillakrylovii* Juz. [7]

*Alchemillamurbeckiana* Buser [7]

SE*Alchemillapavlovii* Juz. [2, 3, 6]

*anserina* (L.) Rydb. [≡ *Potentillaanserina* L.] [1–11, 13–15]

*Aruncussylvester* Kostel. [5]

*Chamaerhodosaltaica* Bunge [1–4, 6–8, 10, 11, 13]

SE*Chamaerhodoscorymbosa* Murav. [5, 9]

*Chamaerhodoserecta* (L.) Bunge [1–13]

*Chamaerhodosgrandiflora* Ledeb. [5]

*Chamaerhodossabulosa* Bunge [3, 6–16]

*Chamaerhodostrifida* Ledeb. [4, 5, 8, 9, 12, 13]

*Coluriageoides* (Pall.) Ledeb. [≡ *Dryasgeoides* Pall.] [3, 6]

*Comarumpalustre* L. [1, 2, 3, 4, 6]

*Cotoneastermegalocarpus* Popov [7]

*Cotoneastermelanocarpus* Lodd. [1–10, 13, 14]

*Cotoneastermongolicus* Pojark. [2–5, 7–9, 12, 13]

*Cotoneasterneopopovii* Czerep. [4]

*Cotoneasteruniflorus* Bunge [1, 2, 3, 7, 8, 13]

*Crataegusdahurica* Koehne [2, 4, 5, 9]

*Crataegusmaximowiczii* C.K.Schneid. [5]

*Crataegussanguinea* Pall. [2, 3, 4, 5, 9]

*Dasiphorafruticosa* (L.) Rydb. [≡ *Potentillafruticosa* L.] [1–9, 11, 13]

*Dasiphoraparvifolia* (Fisch.) Juz. [≡ *Potentillaparvifolia* Fisch.] [2, 3, 4, 6, 8]

*Dryasgrandis* Juz. [1, 7]

*Dryasincisa* Juz. [1]

*Dryasoxyodonta* Juz. [1, 2, 3, 4, 6, 7]

*Dryaspunctata* Juz. [1, 3]

SE*Dryassumneviczii* Serg. [1]

*Farinopsissalesoviana* (Steph.) Chrtek & Soják [≡ *Comarumsalesovianum* (Steph.) Ledeb] [6, 7, 10, 11, 13, 14]

*Filipendulaangustiloba* Maxim. [5, 9]

*Filipendulapalmata* Maxim. [2, 3, 4, 5, 9]

*Filipendulaulmaria* (L.) Maxim. [2, 3, 4]

*Fragariaorientalis* Losinsk. [2, 3, 4, 5]

*Fragariaviridis* Weston [2]

*Geumaleppicum* Jacq. [2, 3, 4, 5, 9]

*Geumrivale* L. [7]

*Malusbaccata* (L.) Borkh. [2, 3, 4, 5, 8, 9]

SE*Potaniniamongolica* Maxim. [11, 12, 13, 16]

*Potentillaacaulis* L. [1–11, 13]

*Potentillaacervata* Soják [= *Potentillachenteica* Soják] [2–5, 8, 9, 13]

*Potentillaagrimonioides* M.Bieb. [= *Potentillalydiae* Kurbatski] [7, 14]

*Potentillaaltaica* Bunge [= PotentillaniveaL.var.pinnatifida Lehm.] [7]

*Potentillaangustiloba* T.T.Yu & C.L.Li [7, 14]

*Potentillaaphanes* Soják [3, 6, 7, 10, 13, 14]

*Potentillaarenosa* (Turcz.) Juz. [≡ Potentillaniveavar.arenosa Turcz.] [2, 3, 4, 6]

*Potentillaasiatica* (Th.Wolf) Juz. [7]

*Potentillaastragalifolia* Bunge [3, 6, 7, 10, 11]

SEPotentilla×burjatica Soják [2]

*Potentillachalchorum* Soják [2, 3, 7, 9]

SEPotentilla×chamaeleo Soják [6, 7, 14]

*Potentillachinensis* Ser. [4, 5, 9]

*Potentillachionea* Soják [1–4, 10, 13, 14]

*Potentillachrysantha* Trevir. [7, 14]

*Potentillaconferta* Bunge [1–9, 12, 13, 14]

E *Potentillacoriacea* Soják [3]

*Potentillacrantzii* (Crantz) Fritsch [7]

*Potentillacrebridens* Juz. [= Potentillaniveavar.elongata Th.Wolf] [1, 2, 3, 10]

*Potentilladesertorum* Bunge [1, 6, 7, 9, 12–14]

SEPotentilla×drymeja Soják [2, 3, 13]

E *Potentillaekaterinae* Kamelin ex Kechaykin [13]

*Potentillaelegans* Cham. & Schltdl. [1]

*Potentillaelegantissima* Polozhij [3]

*Potentillaevestita* Th.Wolf [1–4, 6, 7, 13, 14]

*Potentillaexuta* Soják [3, 7, 13, 14]

*Potentillaflagellaris* D.F.K.Schltdl. [2, 3, 4, 5, 9]

*Potentillafragarioides* L. [2, 3, 4, 5, 7]

*Potentillagelida* C.A.Mey. [1–3, 6, 7, 9, 13, 14]

E *Potentillagobica* Soják [14]

SE*Potentillagracillima* Kamelin [3, 7, 10]

E *Potentillahilbigii* Soják [3]

E *Potentillahubsugulica* Soják [1]

E *Potentillaikonnikovii* Juz. [7, 13]

E *Potentillainopinata* Soják [6, 7]

*Potentillajenissejensis* Polozhij & W.Smirnova [= PotentillaagrimonioidesM.Bieb.var.kobdoensis Soják] [6, 7, 10]

*Potentillakryloviana* Th.Wolf [3, 7, 14]

E *Potentillalaevipes* Soják [7]

E *Potentillalaevissima* Kamelin [7]

*Potentillaleucophylla* Pall. [= *Potentillabetonicifolia* Poir.] [2, 3, 4, 5, 8, 9]

*Potentillalongifolia* D.F.K.Schltdl. [1–13]

E *Potentillamongolica* Krasch. [3, 8]

*Potentillamulticaulis* Bunge [1, 3]

*Potentillamultifida* L. [= *Potentillatenella* Turcz.] [1–14]

*Potentillanivea* L. [1–4, 6, 7, 13, 14]

*Potentillanorvegica* L. [= *Potentillamonspeliensis* L.] [1, 2, 3, 4]

*Potentillanudicaulis* D.F.K.Schltdl. [= *Potentillastrigosa* Pall.] [12]

SEPotentilla×olchonensis Peschkova [6]

*Potentillaornithopoda* Tausch [1–4, 6, 7, 10, 14]

SE*Potentillaozjorensis* Peschkova [1, 3, 4, 7]

*Potentillapamirica* Th.Wolf [6, 7, 10, 14]

*Potentillapamiroalaica* Juz. [14]

*Potentillapensylvanica* L. [≡ *Pentaphyllumpennsylvanicum* (L.) Lunell] [1–11, 13, 14]

*Potentillaregeliana* Th.Wolf [6]

SEPotentilla×rhipidophylla Soják [3]

SE*Potentillarigidula* Th.Wolf [6, 10]

*Potentillasanguisorba* D.F.K.Schltdl. [1, 2, 3, 4, 12, 13]

E *Potentillaschmakovii* Kechaykin [7, 14]

SE*Potentillasergievskajae* Peschkova [5, 8]

*Potentillasericea* L. [1–4, 6–13, 15]

SE*Potentillaserrata* Soják [3]

SE*Potentillasischanensis* Bunge [4, 9]

*Potentillasongorica* Bunge [10, 14]

SE*Potentillastepposa* Soják [7, 10]

*Potentillasubdigitata* T.T.Yu & C.L.Li [= *Potentillajunatovii* Rudaya & A.L.Ebel] [7]

*Potentillasupina* L. [1–12, 14–16]

*Potentillatanacetifolia* D.F.K.Schltdl. [2–9, 12, 13]

*Potentillatergemina* Soják [2, 3, 4, 5, 9]

SE*Potentillatericholica* Sobolevsk. [6, 7]

*Potentillatetrandra* (Bunge) Hook.f. [≡ *Sibbaldiatetrandra* Bunge] [1, 3, 6, 7]

*Potentillaturczaninowiana* Stschegl. [6, 7, 14]

*Potentillaturkestanica* Soják [7, 14]

E *Potentillatytthantha* (Soják) Kechaykin [6, 7]

E Potentilla×vanzhilii Gundegmaa & Kechaykin [3]

*Potentillaverticillaris* Stephan [2, 3, 4, 5, 8, 9]

*Potentillavirgata* Lehm. [1, 3, 4, 6–15]

*Prunusmongolica* Maxim. [≡ *Amygdalusmongolica* (Maxim.) Ricker] [12, 13, 16]

*Prunuspadus* L. [1, 2, 3, 4, 5, 9]

*Prunuspedunculata* (Pall.) Maxim. [≡ *Amygdaluspedunculata* Pall.] [2, 3, 4, 6–13, 16]

*Prunussibirica* L. [≡ *Armeniacasibirica* (L.) Lam.] [2, 3, 4, 5, 9]

*Rosaacicularis* Lindl. [1–9, 13]

*Rosaalbertii* Regel [7]

E *Rosabaitagensis* Kamelin & Gubanov [14]

*Rosabeggeriana* Schrenk [14]

*Rosadavurica* Pall. [2, 4, 5, 9]

*Rosakokanica* (Regel) Regel [7]

Rosalaxavar.kaschgarica (Rupr.) Y.L.Han [= *Rosakaschgarica* Rupr.] [14, 15]

RosalaxaLindl.var.laxa [6, 7, 13, 14, 15]

*Rosaoxyacantha* M.Bieb. [1, 2, 3, 7]

*Rosaplatyacantha* Schrenk [14]

*Rosaspinosissima* L. [3, 7, 14]

*Rosaxanthina* Lindl. [9]

*Rubusarcticus* L. [1, 2, 3, 4]

*Rubuschamaemorus* L. [2]

*Rubushumilifolius* C.A.Mey. [1, 2]

*Rubussachalinensis* H.Lév. [1, 2, 3, 4, 7, 8]

*Rubussaxatilis* L. [1, 2, 3, 4, 5, 9]

*Sanguisorbaalpina* Bunge [3, 6, 7]

*Sanguisorbaofficinalis* L. [1–11]

*Sanguisorbaparviflora* (Maxim.) Takeda [9]

*Sanguisorbatenuifolia* Fisch. [4, 9]

*Sibbaldiaprocumbens* L. [2, 7]

*Sibbaldiantheadpressa* (Bunge) Juz. [≡ *Sibbaldiaadpressa* Bunge] [1–13, 15, 16]

*Sibbaldianthebifurca* (L.) Kurtto & T.Erikss. [≡ *Potentillabifurca* L.] [1–14]

*Sibbaldiantheimbricata* (Kar. & Kir.) Mosyakin & Shiyan [≡ *Potentillaimbricata* Kar. & Kir.] [6, 7, 10, 14]

*Sibbaldiantheorientalis* (Soják) Mosyakin & Shiyan [= Potentillabifurcavar.major Ledeb.] [7, 8, 9, 14]

*Sibbaldianthesemiglabra* (Soják) Mosyakin & Shiyan [≡ *Potentillasemiglabra* Juz.] [5, 9]

SE*Sibbaldianthesericea* Grubov [7, 8, 12, 13]

*Sibiraealaevigata* (L.) Maxim. [7]

*Sorbariasorbifolia* (L.) A.Braun [4, 5]

SorbusaucupariaL.subsp.glabrata (Wimm. & Grab.) Hedl. [= *Sorbussibirica* Hedl.] [1, 2, 3, 4, 5]

*Spiraeaalpina* Pall. [1, 2, 3, 6, 7]

*Spiraeaaquilegiifolia* Pall. [1–5, 8, 9, 12, 13]

*Spiraeachamaedryfolia* L. [5]

*Spiraeadahurica* (Rupr.) Maxim. [2, 4]

*Spiraeaelegans* Pojark. [4]

*Spiraeaflexuosa* Fisch. [1–6, 8, 9, 13]

*Spiraeahypericifolia* L. [2–4, 6, 7, 9, 10, 12, 14]

SpiraeamediaF.Schmidtsubsp.media [= *Spiraeasericea* Turcz.] [1–8, 13]

*Spiraeapubescens* Turcz. [4, 5, 9]

*Spiraeasalicifolia* L. [2, 3, 4, 5, 9]

**95. Rubiaceae** Juss. (3 genera and 13 taxa)

E *Asperulagobicola* Grubov [= *Asperulasaxicola* Grubov] [13, 16]

*Galiumamblyophyllum* Schrenk [1, 2, 14]

*Galiumboreale* L. [1–10, 13, 14]

*Galiumdahuricum* Turcz. [2]

*Galiumdensiflorum* Ledeb. [3, 6, 7, 14]

*Galiumhumifusum* M.Bieb. [3, 7, 13]

*Galiumsongaricum* Schrenk [1, 2, 3]

*Galiumspurium* L. [1–8, 10, 13, 14]

*Galiumtrifidum* L. [2–5, 7, 10, 14]

*Galiumuliginosum* L. [1, 2, 3, 4, 10]

GaliumverumL.subsp.verum [= *Galiumdensiflorum* Ledeb.] [1–10, 13, 14]

Galiumverumsubsp.wirtgenii (F.W.Schultz) Oborny [7]

*Rubiacordifolia* L. [≡ *Galiumcordifolium* (L.) Kuntze] [2–5, 8, 9, 12, 13]

**96. Ruppiaceae** Horan. (1 genus and 1 species)

*Ruppiamaritima* L. [10]

**97. Rutaceae** Juss. (2 genera and 2 species)

*Haplophyllumdauricum* (L.) G.Don [2–6, 8, 9, 11–14, 16]

*Dictamnusalbus* L. [5, 9]

**98. Salicaceae** Mirb. (2 genera and 47 species)

*Populuseuphratica* Olivier [12–16]

*Populuslaurifolia* Ledeb. [= *Populuspilosa* Rehder] [2–4, 6, 7, 10, 13, 14]

*Populussimonii* Carrière [9]

*Populussuaveolens* Fisch. [1, 2, 3, 4]

*Populustremula* L. [1–5, 7–9, 11]

*Salixabscondita* Laksch. [1, 2, 4, 5, 13]

*Salixalatavica* Kar. [6, 7]

*Salixarctica* Pall. [1, 3, 6, 7]

*Salixbebbiana* Sarg. [1–10, 12, 13]

*Salixberberifolia* Pall. [1, 2, 3, 6, 7, 13]

*Salixbrachypoda* (Trautv. & C.A.Mey.) Kom. [2, 4, 5, 9]

*Salixcaesia* Vill. [1–4, 6, 7, 10, 14]

*Salixdivaricata* Pall. [1–4, 6, 7, 13]

*Salixglauca* L. [1, 2, 3, 6, 7, 13]

*Salixgmelinii* Pall. [= *Salixdasyclados* Wimmer] [2, 4, 6, 7, 8, 10]

*Salixgordejevii* Y.L.Chang & Skvortsov [5, 8, 9]

*Salixhastata* L. [1, 6, 7, 10, 13]

*Salixjenisseensis* (F.Schmidt) Flod. [1, 6, 7]

*Salixkochiana* Trautv. [1–5, 7, 10]

*Salixledebouriana* Trautv. [1–4, 6, 7, 9–15]

*Salixmicrostachya* Turcz. [2–6, 8, 9, 10]

*Salixmiyabeana* Seemen [1–5, 8, 9]

*Salixmyrtilloides* L. [1, 2, 3]

SE*Salixnasarovii* A.K.Skvortsov [1]

*Salixnipponica* Franch. & Sav. [9]

*Salixnummularia* Andersson [2, 3, 6, 7]

*Salixpolaris* Wahlenb. [1]

*Salixpseudopentandra* (Flod.) Flod. [= Salixpentandravar.intermedia Nakai] [1–10, 12, 13]

*Salixpyrolifolia* Ledeb. [1–4, 6, 7, 10]

*Salixrectijulis* Ledeb. [1, 2, 3, 6, 7]

*Salixrecurvigemmata* A.K.Skvortsov [= *Salixrecurvigemmis* A.K.Skvortsov] [1, 3, 6]

*Salixreticulata* L. [1, 3, 6, 7]

*Salixrhamnifolia* Pall. [1–6, 9]

*Salixrorida* Laksch. [2, 3, 4, 5, 9]

*Salixrosmarinifolia* L. [1–4, 6, 10, 14]

*Salixsajanensis* Nasarow [1, 6, 7]

*Salixsaposhnikovii* A.K.Skvortsov [1, 3, 6, 7]

*Salixsaxatilis* Turcz. [1, 2, 3]

*Salixschwerinii* E.L.Wolf [2, 3, 4, 5, 9]

*Salixtaraikensis* Kimura [1–5, 7, 13]

*Salixtenuijulis* Ledeb. [3, 7, 14, 15]

*Salixtriandra* L. [1, 3, 4, 14]

*Salixturanica* Nasarow [6, 7, 10, 14]

*Salixturczaninowii* Laksch. [1, 2, 6, 7]

*Salixudensis* Trautv. & C.A.Mey. [9]

*Salixvestita* Pursh [1, 3, 6, 7]

*Salixviminalis* L. [3, 6, 7, 10, 11, 14]

**99. Santalaceae** R.Br. (1 genus and 6 species)

*Thesiumchinense* Turcz. [9]

*Thesiumlongifolium* Turcz. [2, 3, 4]

*Thesiumrefractum* C.A.Mey. [1–10, 13]

*Thesiumrepens* Ledeb. [1, 2, 3, 4]

SE*Thesiumsaxatile* Turcz. [1, 3–6, 8–10]

SE*Thesiumtuvense* Krasnob. [5, 10]

**100. Saxifragaceae** Juss. (5 genera and 21 taxa)

*Bergeniacrassifolia* (L.) Fritsch [1, 2, 3, 4, 7]

*Chrysospleniumnudicaule* Bunge [6]

SE*Chrysospleniumpeltatum* Turcz. [1, 3]

SE*Chrysospleniumsedakowii* Turcz. [2, 3, 8]

*Chrysospleniumserreanum* Hand.-Mazz. [= Chrysospleniumalternifoliumsubsp.sibiricum (Ser.) Hultén] [1, 2, 4]

*Micranthesdavurica* (Willd.) Small [≡ *Saxifragadavurica* Willd.] [2]

*Micranthesfoliolosa* (R.Br.) Gornall [≡ *Saxifragafoliolosa* R.Br.] [1, 2]

*Micrantheshieraciifolia* (Waldst. & Kit.) Haw. [≡ *Saxifragahieraciifolia* Waldst. & Kit.] [1, 2, 3, 6, 7]

*Micranthesmelaleuca* (Fisch.) Losinsk. [≡ *Saxifragamelaleuca* Fisch.] [1, 2, 6, 7]

Micranthesnelsonianasubsp.aestivalis (Fisch. & C.A.Mey.) Elven & D.F.Murray [≡ *Saxifragaaestivalis* Fisch. & C.A.Mey.] [1, 2, 3, 6]

*Micranthesnivalis* (L.) Small [≡ *Saxifraganivalis* L.] [1]

*Mitellanuda* L. [1, 2, 4]

*Saxifragabronchialis* L. [= *Saxifragacaulescens* Sipliv., = *Saxifragaspinulosa* Adams] [2, 3, 4, 8]

*Saxifragacernua* L. [1–3, 6–9, 13]

*Saxifragahirculus* L. [1–7, 13, 14]

*Saxifragamacrocalyx* Tolm. [= *Saxifragaflagellaris* Willd.] [1, 3, 6, 7, 13, 14]

SaxifragaoppositifoliaL.subsp.oppositifolia [= *Saxifragaasiatica* Hayek] [1, 3, 6, 7]

*Saxifragasetigera* Pursh [1, 2, 3, 6, 7, 13]

*Saxifragasibirica* L. [1–3, 6–8, 10, 13, 14]

*Saxifragaterektensis* Bunge [1, 3, 6, 7]

**101. Scheuchzeriaceae** F.Rudolphi (1 genus and 1 species)

*Scheuchzeriapalustris* L. [2]

**102. Scrophulariaceae** Juss. (3 genera and 6 species)

*Limosellaaquatica* L. [1–4, 6, 7, 9–11, 13, 14]

*Scrophulariaaltaica* Murray [1, 3, 6, 7]

*Scrophulariacanescens* Bong. [= *Scrophulariahilbigii* Jäger] [13, 14]

*Scrophulariaincisa* Weinm. [2–4, 6–15]

*Scrophulariaumbrosa* Dumort. [10]

*Verbascumthapsus* L. [4]

**103. Solanaceae** Juss. (4 genera and 9 taxa)

*Hyoscyamusniger* L. [2–5, 7–10, 12, 13]

*Hyoscyamuspusillus* L. [6, 7, 10, 14, 15]

Lyciumchinensevar.potaninii (Pojark.) A.M.Lu [≡ *Lyciumpotaninii* Pojark.] [16]

*Lyciumruthenicum* Murray [10, 11, 13–16]

*Lyciumtruncatum* Y.C.Wang [10, 12, 15, 16]

E *Physochlainaalbiflora* Grubov [3, 4]

*Physochlainaphysaloides* (L.) G.Don [1, 3–9, 11–13]

*Solanumkitagawae* Schönb.-Tem. [3, 6, 9, 14]

*Solanumseptemlobum* Bunge [4, 8, 9, 12]

**104. Tamaricaceae** Link (3 genera and 13 taxa)

*Myricariabracteata* Royle [1, 7, 11–14, 16]

*Myricarialongifolia* Ehrenb. [2–4, 6, 7, 11]

*Reaumuriasoongarica* Maxim. [3, 6–16]

*Tamarixarceuthoides* Bunge [10, 14, 15]

*Tamarixelongata* Ledeb. [11, 14]

*Tamarixgracilis* Willd. [13, 15, 16]

*Tamarixhispida* Willd. [13]

*Tamarix×karelinii* Bunge [10, 14–16]

*Tamarixkasahorum* Gorschk. [15, 16]

*Tamarixlaxa* Willd. [11, 12, 15]

*Tamarixleptostachya* Bunge [10, 13–16]

*Tamarixramosissima* Ledeb. [10, 12–16]

*Tamarixsmyrnensis* Bunge [12, 14, 16]

**105. Thymelaeaceae** Juss. (2 genera and 3 species)

*Diarthronaltaicum* (Thiéb.-Bern.) Kit Tan [≡ *Stelleraaltaica* Thiéb.-Bern.] [7]

*Diarthronlinifolium* Turcz. [2, 3, 4, 5]

*Stellerachamaejasme* L. [2, 3, 4, 5, 9]

**106. Tofieldiaceae** Takht. (1 genus and 1 species)

*Tofieldiacoccinea* Richardson [1]

**107. Typhaceae** Juss. (2 genera and 12 species)

*Sparganiumemersum* Rehmann [3, 5, 6, 7, 9, 10]

*Sparganiumglomeratum* (Laest.) Beurl. [2, 4]

*Sparganiumnatans* L. [7, 10]

*Sparganiumstoloniferum* (Graebn.) Buch.-Ham. [1, 4, 8, 9, 10]

*Typhaangustifolia* L. [1, 10]

*Typhadomingensis* Pers. [4, 10]

*Typhajoannis* Mavrodiev [9]

*Typhalatifolia* L. [2, 5]

*Typhalaxmannii* Lepech. [3–6, 8, 9, 11]

*Typhaminima* Funck [9, 10]

*Typhaorientalis* C.Presl [5]

*Typhatzvelevii* Mavrodiev [4]

**108. Ulmaceae** Mirb. (1 genus and 3 taxa)

Ulmusdavidianavar.japonica (Rehder) Nakai [4, 5, 8]

*Ulmusmacrocarpa* Hance [2, 4, 5, 8, 9, 12]

*Ulmuspumila* L. [2–5, 7–9, 11, 13, 16]

**109. Urticaceae** Juss. (2 genera and 4 taxa)

*Parietariadebilis* G.Forst. [2–4, 6, 9, 10]

*Urticaangustifolia* Fisch. [1–5, 7, 9, 10]

*Urticacannabina* L. [2–10, 12–14]

UrticadioicaL.subsp.sondenii (Simm.) Hyl. [≡ *Urticasondenii* (Simm.) Avror] [7, 14]

**110. Violaceae** Batsch. (1 genus and 27 taxa)

Note: Recently, Esput (2020) critically revised the genus *Viola* L in the Russian Far East and adjacent territories. In this study, accepted species and nomenclature mostly follow Esput (2020).

*Violaacuminata* Ledeb. [4, 5, 9]

SE*Violaalexandrowiana* (W.Becker) Juz. [4]

*Violaaltaica* Ker Gawl. [3, 7]

*Violaarvensis* Murray [4]

*Violabiflora* L. [1, 2, 3, 7]

*Violabrachyceras* Turcz. [2]

*Violacollina* Besser [5, 7]

*Violadactyloides* Schult. [2, 3, 4, 5, 6]

*Violadisiuncta* W.Becker [7]

*Violadissecta* Ledeb. [1–5, 7, 9, 13]

*Violaepipsiloides* Á.Löve & D.Löve [1, 2]

*Violagmeliniana* Schult. [1, 2, 3, 4, 5]

*Violaincisa* Turcz. [2, 4]

SE*Violaircutiana* Turcz. [2]

*Violamacroceras* Bunge [7]

*Violamauritii* Teplouchow [1, 4, 5, 7, 9, 13]

*Violamirabilis* L. [2, 5]

*Violanemoralis* Kuetz. [2]

*Violapatrinii* Ging. [2, 5, 7, 9, 10, 14]

*Violarudolfii* Vl.V.Nikitin [4, 5]

*Violarupestris* F.W.Schmidt [2, 3, 4, 6, 7]

*Violasacchalinensis* H.Boissieu [2, 4, 5]

Viola×schauloi Vl.V.Nikitin [2, 4]

*Violaselkirkii* Pursh [2]

Violatenuicornissubsp.trichosepala W.Becker [4]

*Violauniflora* L. [1, 2, 3, 4, 7]

*Violavariegata* Fisch. [1, 4, 5]

**111. Zygophyllaceae** R.Br. (2 genera and 13 taxa)

*Tribulusterrestris* L. [3, 5–16]

*Zygophyllumbrachypterum* Kar. & Kir. [6, 10]

*Zygophyllumgobicum* Maxim. [14, 15]

*Zygophyllumkaschgaricum* Boriss. [≡ *Sarcozygiumkaschgaricum* (Boriss.) Y.X.Liou] [12–16]

*Zygophyllummacropterum* C.A.Mey. [= *Zygophyllumpinnatum* Cham. & Schltdl.] [7, 14]

SE*Zygophyllummelongena* Bunge [3, 6, 7, 10, 11, 13, 14]

SE*Zygophyllummucronatum* Maxim. [15, 16]

E *Zygophyllumneglectum* Grubov [10, 13, 14, 16]

*Zygophyllumpotaninii* Maxim. [6, 7, 12–16]

*Zygophyllumpterocarpum* Bunge [6, 7, 10–16]

Zygophyllumrosowiivar.latifolium (Schrenk) Popov [13–16]

ZygophyllumrosowiiBungevar.rosowii [3, 7, 8, 10–16]

*Zygophyllumxanthoxylon* (Bunge) Maxim. [7, 8, 10–16]

## Supplementary materials

Supplementary material 1 Appendix 1 Data type Checklist Explanation note List of new species (marked by red color) and new records to the flora of Mongolia, since Urgamal et al (2014). https://binary.pensoft.net/file/657623This dataset is made available under the Open Database License (http://opendatacommons.org/licenses/odbl/1.0/). The Open Database License (ODbL) is a license agreement intended to allow users to freely share, modify, and use this Dataset while maintaining this same freedom for others, provided that the original source and author(s) are credited.Baasanmunkh et al.

Supplementary material 2 Appendix 2 Data type Checklist Explanation note Changes of accepted names compared with Urgamal et al (2014). https://binary.pensoft.net/file/657624This dataset is made available under the Open Database License (http://opendatacommons.org/licenses/odbl/1.0/). The Open Database License (ODbL) is a license agreement intended to allow users to freely share, modify, and use this Dataset while maintaining this same freedom for others, provided that the original source and author(s) are credited.Baasanmunkh et al.
